# Pd(OAc)_2_-catalyzed dehydrogenative C–H activation: An expedient synthesis of uracil-annulated β-carbolinones

**DOI:** 10.3762/bjoc.11.146

**Published:** 2015-08-04

**Authors:** Biplab Mondal, Somjit Hazra, Tarun K Panda, Brindaban Roy

**Affiliations:** 1 Department of Chemistry, University of Kalyani, Kalyani, Nadia-741235, West Bengal, India; 2 Department of Chemistry, Indian Institute of Technology Hyderabad, Ordnance Factory Estate, Yeddumailaram - 502205, Telangana, India

**Keywords:** β-carbolinones, cyclization, dehydrogenative C–H activation, Pd(OAc)_2_, uracil

## Abstract

An intramolecular dehydrogenative C–H activation enabled an efficient synthesis of an uracil-annulated β-carbolinone ring system. The reaction is simple, efficient and high yielding (85–92%).

## Introduction

**Figure 1:**
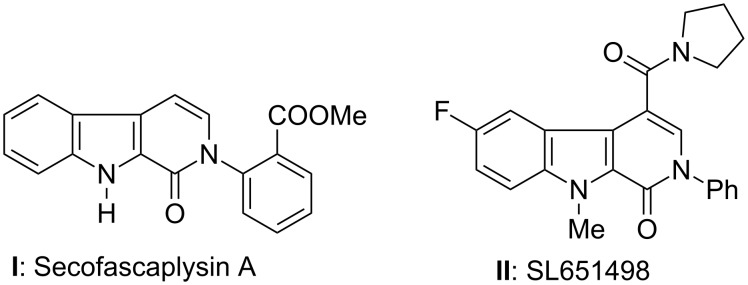
Naturally occurring β-carbolinones.

The presence of a β-carbolinone skeleton in various natural products, such as secofascaplysin A (**I**), SL651498 (**II**) (Figure 1), and their remarkable biological and pharmacological properties make them stand out amongst all the carboline class of compounds [1–12]. For example, SL651498 was documented as a potential drug development candidate in a research program designed to discover subtype-selective GABA_A_ receptor agonists for the treatment of muscle spasms and generalized anxiety disorder [13]. β-Carbolinones such as strychnocarpine, the alkaloid from *Alstonia venenata*, and substituted 1-oxo-13-carbolines were also shown to have serotonin-receptor-binding activity (5-HT receptor) [14]. Moreover the natural and synthetic β-carbolines are also known to show anticancer activity against colon and lung cancers, and some β-carbolinones act as biological control agents for receptor research on bioenzyme inhibitors, such as the inhibition of HLE (human leukocyte elastase) [15–18].

Uracil, on the other hand, is one of the four nucleobases of RNA. It holds immense importance from a pharmaceutical and biological point of view [19]. For example, 5-fluorouracil [20] and other uracil-based molecules [21–23] such as 3’-azido-3’-deoxythymidine (AZT), 2’,3’-dideoxycytidine (DDC), (*E*)-5-[2-(bromovinyl)-2’-deoxyuridine] (BVDU), are active against cancer and the HI virus [24–31].

Diverse approaches towards the synthesis of β-carbolinones have been developed [32–36], such as an intramolecular Heck reaction strategy of 2- and 3-iodoindoles for the synthesis of β- or γ-carbolinones by Beccalli et al. [32], AuCl_3_ and Pd-catalyzed cycloisomerization of indole-2-carboxamides to β-carbolinones [33–35], Pd-catalyzed dehydrogenative annulation of indole-carboxamides with alkynes etc [36]. The development of metal-catalyzed C–H activation reaction has revolutionized the way a synthetic chemist now approaches a traditional C–C bond disconnection [32–44]. Dehydrogenative C–H activation [45–52] is the most elegant alternative in this endeavor as it avoids pre-functionalization of any C–H bond beforehand. But regioselectivity is the main problem in this type of reaction due to the ubiquitous presence of various C–H bonds in a simple organic molecule. However, sometimes the issue of regioselectivity can be resolved by the electronic property of the substrate itself. Pioneering work published by Fagnou et al. shows how a catalyst inverts its selectivity and reactivity between the coupling partners to achieve indole C3-arylation in a cross coupling reaction of an unactivated arene and *N*-acetylindole [53]. Driven by the same logic and guided by our previous work we envisioned that an intramolecular dehydrogenative cross coupling reaction could be achievable between the electron deficient uracil C6–H bond adjacent to the nitogen atom and the electron rich indole C3–H bond for the synthesis of uracil annulated β-carbolinones [54–56]. Herein, we report our novel approach towards the synthesis of uracil annulated β-carbolinones via an intramolecular dehydrogenative coupling reaction of indole-2-carboxamides.

## Results and Discussion

**Scheme 1:**
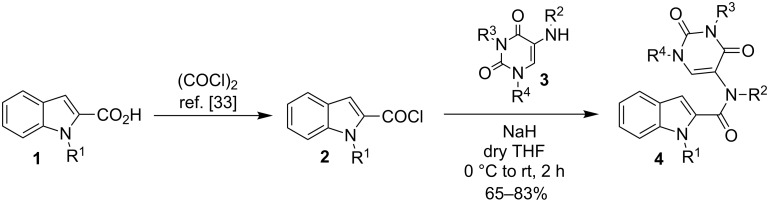
Preparation of starting substrate.

We started our investigation with the preparation of amide precursors **4** from *N*-substituted indole-2-carboxylic acids **1** (Scheme 1). The acids were treated with oxalyl chloride at room temperature [33] and the resulting acid chloride obtained was transformed to the amide by the reaction with amine **3** in dry THF using NaH as a base.

The amide precursor was then subjected to a series of reactions in pursuit of the best reaction conditions for the dehydrogenative cross-coupling process. Assuming that the reaction goes through an electrophilic metallation pathway, it was projected that Pd(OAc)_2_ would be an excellent starting point for catalyst screening. The amide **4a** (R^1^ = R^2^ = R^3^ = R^4^ = Me) was used as a model substrate for this dehydrogenative coupling reaction. The reaction was set up in the presence of Pd(OAc)_2_ (10 mol %), Cu(OAc)_2_ (2 equiv) in DMF under open air at 70 °C (Table 1, entry 1). After 8 h we obtained 35% yield of **5a** with 52% conversion of starting material. Increasing the temperature to 90 °C (Table 1, entry 2) afforded 63% yield of **5a** with 80% conversion of **4a**. Then different oxidants [Cu(OTf)_2_, PhI(OAc)_2_, K_2_S_2_O_8,_ (NH_4_)_2_S_2_O_8, _*p*-benzoquinone (BQ), oxone, AgOAc, molecular oxygen] were examined under these reaction conditions. With Cu(OTf)_2_ (Table 1, entry 3) and oxone (Table 1, entry 8), total recovery of starting material was observed while PhI(OAc)_2_ (Table 1, entry 4), (NH_4_)_2_S_2_O_8_ (Table 1, entry 6) showed total decomposition of starting material. With BQ the yield was almost the same (65%) as with Cu(OAc)_2_ (Table 1, entry 7). The use of K_2_S_2_O_8_ increased the yield of **5a** to 83% (Table 1, entry 5) with complete conversion of starting material; AgOAc further increased the yield to 91% (Table 1, entry 9). Molecular oxygen also used as an oxidant resulted in a low 30% yield of **5a** (Table 1, entry 10) with 51% conversion of **4a**. Examination of different solvents led to determining that the polar solvents (DMF/DMSO (9:1), DMSO, DMAc, Table 1, entries 11, 12, 13) were far superior compared to non-polar solvent toluene (Table 1, entry 14). But the optimal result was obtained with the use of DMF (Table 1, entry 9). Testing the efficiency of other Pd catalysts for this reaction revealed that Pd_2_dba_3_, i.e., Pd(0) did not show any catalytic activity (Table 1, entry 17). The yield decreased sharply for other Pd catalysts such as PdCl_2_, Pd(CH_3_CN)_2_Cl_2_ and Pd(PPh_3_)_2_Cl_2_ presumably be due to the less electrophilic nature of these catalysts (Table 1, entries 15, 16, 18).

**Table 1:** Optimization of intramolecular dehydrogenative coupling.

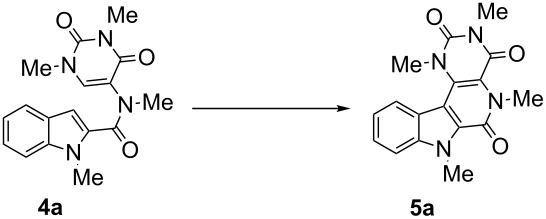						

Entry	Catalyst	Oxidant	Solvent	Temp (°C)	Conversion (%)^a^	Yield (%)^b^

1	Pd(OAc)_2_	Cu(OAc)_2_	DMF	70	52	35
2	Pd(OAc)_2_	Cu(OAc)_2_	DMF	90	80	63
3	Pd(OAc)_2_	Cu(OTf)_2_	DMF	90	0	0
4	Pd(OAc)_2_	PhI(OAc)_2_	DMF	90	0	0
5	Pd(OAc)_2_	K_2_S_2_O_8_	DMF	90	100	83
6	Pd(OAc)_2_	(NH_4_)_2_S_2_O_8_	DMF	90	0	0
7	Pd(OAc)_2_	BQ	DMF	90	78	65
8	Pd(OAc)_2_	oxone	DMF	90	0	0
9	Pd(OAc)_2_	AgOAc	DMF	90	100	91
10	Pd(OAc)_2_	O_2_	DMF	90	51	30
11	Pd(OAc)_2_	AgOAc	DMF/DMSO (9:1)	90	100	82
12	Pd(OAc)_2_	AgOAc	DMSO	90	100	78
13	Pd(OAc)_2_	AgOAc	DMAc	90	100	80
14	Pd(OAc)_2_	AgOAc	toluene	90	40	30
15	PdCl_2_	AgOAc	DMF	90	83	52
16	Pd(CH_3_CN)_2_Cl_2_	AgOAc	DMF	90	70	35
17	Pd_2_(dba)_3_	AgOAc	DMF	90	0	0
18	Pd(PPh_3_)_2_Cl_2_	AgOAc	DMF	90	40	32

All reactions were performed with 1 equiv starting material (**4**), 10 mol % Pd-catalyst, 2 equiv oxidant in 5 mL solvent at mentioned tempetature, 8 h under open air. ^a^Calculation on the basis of isolation of starting material. ^b^Isolated yield.

**Scheme 2:**
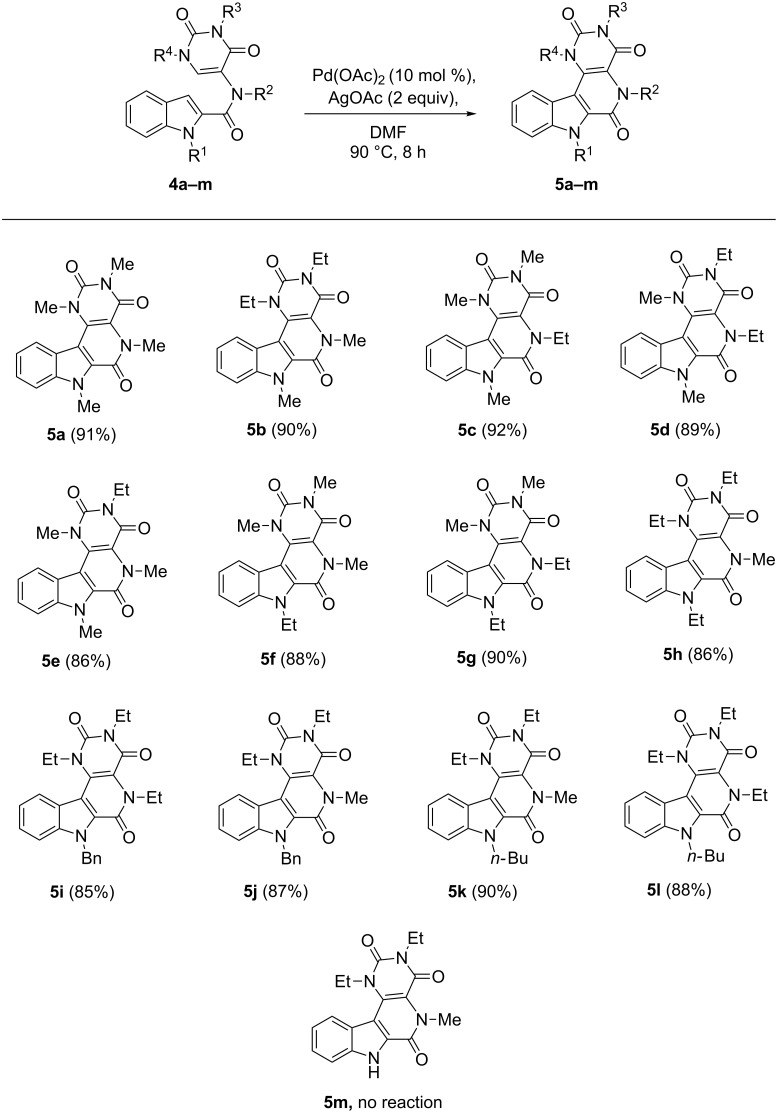
Synthesis of various β-carbolinone derivatives.

With the optimized reaction conditions in hand (10 mol % Pd(OAc)_2_, 2 equiv AgOAc in DMF at 90 °C for 8 h), we further explored the substrate scope of the reaction (Scheme 2).

**Figure 2:**
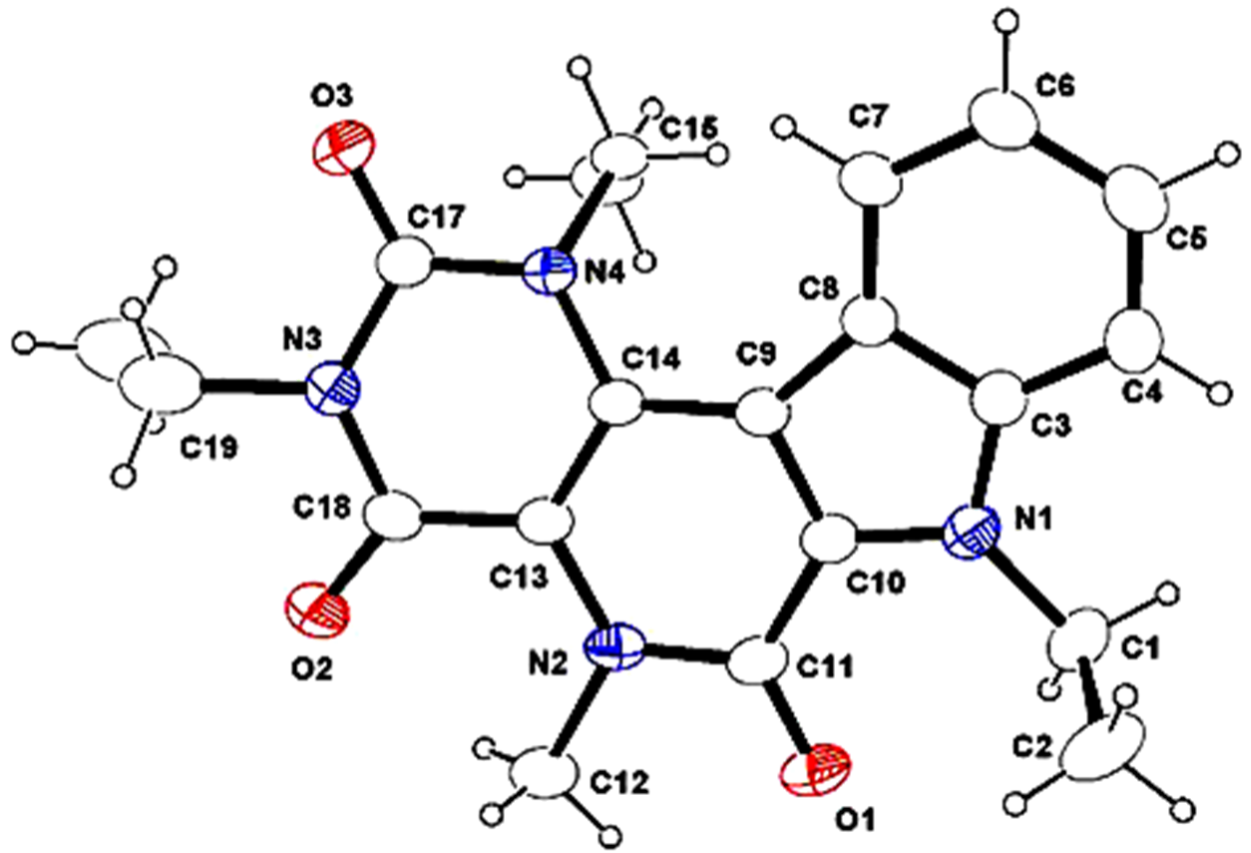
ORTEP diagram of **5h**.

All the reactions went very smoothly giving excellent yields in the range of (85–92%). No distinct steric influence was noticed when the indole-*N*-methyl group was replaced by ethyl, butyl or benzyl groups. However, the reaction did not proceed at all with the unsubstituted indole precursor (R^1^ = H, **4m**), this result may be explained with potential coordination of the Pd catalyst between the indole nitrogen and amide carbonyl oxygen. A representative X-ray crystal structure of β**-**carbolinone derivative **5h** was obtained [57] (Figure 2).

**Scheme 3:**
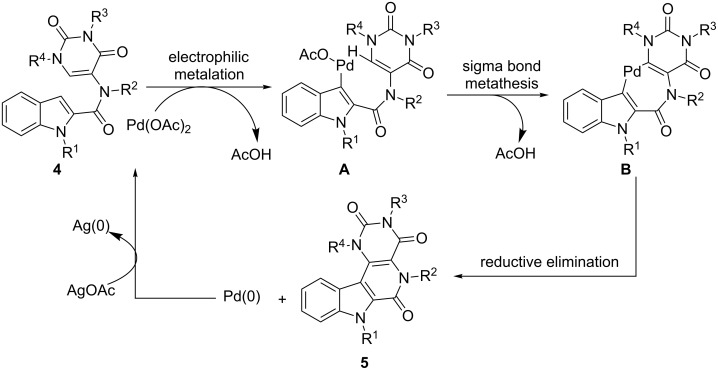
Proposed mechanistic pathway.

The inactivity of Pd(0) (Table 1, entry 17), and inferior reactivity of other less electrophilic Pd catalysts indicates a mechanistic pathway that commence with electrophilic metalation at the indole C3 postion. The nucleophilicity of indole at C3 is well known [58] and a similar kind of electrophilic reaction leads to the intermediate **A** (Scheme 3). We believe this intermediate then undergoes a σ-bond metathesis reaction to form a seven membered palladacycle **B** which in turn produces the product β-carbolinones after reductive elimination from the 7-membered palladacycle **B**. The catalytic cycle is completed by AgOAc.

## Conclusion

In conclusion we have developed an elegant method for the preparation of uracil annulated β-carbolinones via a high yielding dehydrogenative C–H activation process. The key to the success of this reaction is the complementary electronic properties of the indole C3–H bond and the uracil C6–H bond. It is anticipated this efficient and atom economic approach can be emulated for the preparation of other β-carbolinones as well, and further results in this regard will be reported in due course.

## Experimental

Representative procedure for the preparation of uracil annulated β-carbolinones (**5a–m**):

In a flame-dried round bottomed flask equipped with a magnetic bar, a mixture of 1 equiv starting material **4**, 5 mL dry DMF, 2 equiv of AgOAc and 10 mol % Pd(OAc)_2_ was taken and stirred at room temperature for 5 min. Then the reaction mixture was heated in an oil bath fixed at 90 °C for 8 h under air. Completion of the reaction was monitored by checking TLC. The reaction mixture was cooled to room temperature, diluted with water and 50 mL of EtOAc and passed through a pad of celite. The organic layer was washed with H_2_O (2 × 10 mL) and saturated NaCl (aq) (1 × 10 mL). The organic part was dried over Na_2_SO_4_, evaporated and purified by flash chromatography, using ethyl acetate/petroleum ether (2:8) as the eluent to afford product **5**. For details see Supporting Information File 1.

## Supporting Information

File 1 Experimental and analytical data.
