# Supplementary material for: Pd(OAc)2-catalyzed dehydrogenative C–H activation: An expedient synthesis of uracil-annulated β-carbolinones
Source: Beilstein J Org Chem. 2015 Aug 4;11:1360–6. doi: 10.3762/bjoc.11.146 (PMC4578345; doi:10.3762/bjoc.11.146)
Supplement: File 1 — Experimental and analytical data. [file Beilstein_J_Org_Chem-11-1360-s001.pdf]

Supporting Information

for

$Pd(OAc)_2$-catalyzed dehydrogenative C–H activation: An expedient synthesis of uracil-annulated β-carbolinones

Biplab Mondal[1], Somjit Hazra[1], Tarun K. Panda[2] and Brindaban Roy*[1]

Address: [1]Department of Chemistry, University of Kalyani, Kalyani, Nadia-741235, West Bengal, India and [2]Department of Chemistry, Indian Institute of Technology Hyderabad, Ordnance Factory Estate, Yeddumailaram - 502205, Telangana, India

Email: Brindaban Roy* - broybsku@gmail.com

* Corresponding author

Table of contents

(1) General remarks--s2

(2) General procedure for preparation of starting material and final product------------------s2

(3) Characterization data---s4

(4) References---------------s16

(5) ^{1}H and ^{13}C NMR Spectra --s17

(1) General remarks

Melting points were measured in open capillaries and are uncorrected. The ^{1}H and ^{13}C NMR spectroscopic data were recorded in $CDCl_3$ with TMS as the internal standard (chemical shift in *δ*) with a Bruker DPX-400 spectrometer. Data are reported as follows: chemical shifts, multiplicity (s = singlet, d = doublet, t = triplet, q = quartet, quintet = quintet, m = multiplet, brs = broad singlet), coupling constant (Hz). In case of **5f** two peaks are missing at 40.13 ppm and at 139.86 ppm and in case of starting materials (except **4i** and **4j)** one peak is missing in the region ~138 ppm in ^{13}C NMR (assumed from DEPT NMR of **4c)** and equivalent carbons are marked in ^{13}C spectra of **5f** and **4a**. HRMS (ESI) spectra were taken using Waters Xevo G2 QTof mass spectrometer. Silica gel [60-120 mesh (Rankem, India), (230–400 mesh (Spectrochem, India)] was used for the chromatographic separations. Silica gel G and GF 254 (CDH, India) was used for TLC. Petroleum ether refers to the fraction boiling between 60 and 80 °C.

(2) (a) General procedure[1] for the preparation of starting material (4):

To a stirred solution of 1 equiv. of indole-2-carboxylic acid **1** in 10 mL of dry benzene containing 2 drops of *N*, *N*-dimethylformamide, 1.5 equiv. of oxalyl chloride was added. The resulting solution was stirred at rt for 3 h, subsequently, the solvent was removed under reduced pressure and taken up in 5 mL of dry THF. In a separate flask, 1 equiv. of 5-amino uracil derivative **3** dissolved in 3 mL of dry THF was added to a suspension of 1.2 equiv. of NaH in 5 mL of dry THF at 0 °C. The solution was allowed to stir at 0 ^{o}C for 30 min and the preformed acid chloride **2** was added drop wise. The resulting solution was allowed to warm to rt and stirred for 2 h. The solution was quenched with water and extracted with DCM. The combined organic layer was washed with brine, dried over Na_2SO_4 and concentrated under reduced pressure. The crude residue was subjected to flash silica gel chromatography to give the corresponding amide **4**.

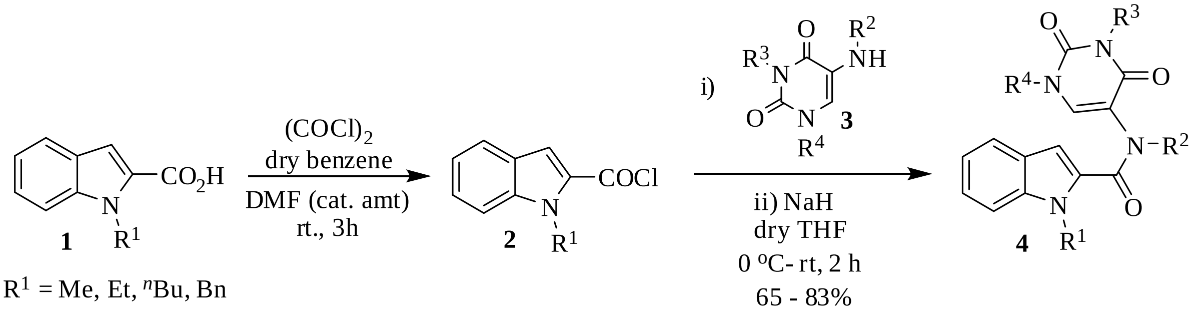

(b) General procedure for the preparation of uracil annulated β-carbolinones (5):

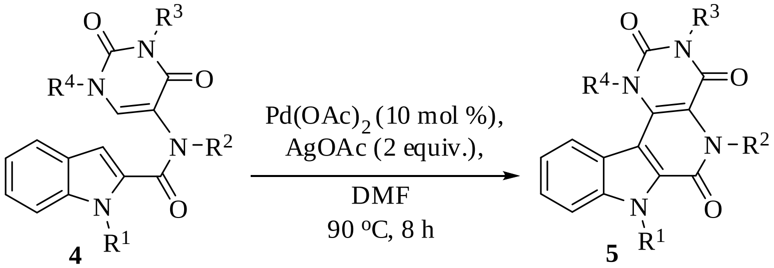

In a flame dried round bottomed flask equipped with a magnetic bar, a mixture of 1 equiv. starting material (**4**), 5 mL dry DMF, 2 equiv. of AgOAc and 10 mol % $Pd(OAc)_2$ was taken and stirred at room temperature for 5 min. Then the reaction mixture was heated in an oil bath fixed at 90 ^{o}C for 8 h under air. Completion of the reaction was monitored by checking TLC. The reaction mixture was cooled to room temperature, diluted with water and 50 mL of EtOAc and passed through a celite pad. The ethyl acetate part was washed with H_2O (2 ×10 mL) and saturated NaCl (aq) (1 ×10 mL). The organic part was dried over Na_2SO_4, evaporated and purified by flash chromatography, using ethyl acetate/petroleum ether (2:8) as the eluent to afford product **5**.

(3) Charcterization data

(a) Characterization data of uracil annulated β-carbolinones (5):

1,3,5,7-tetramethyl-5,7-dihydro-1*H*-pyrimido[4',5':5,6]pyrido[3,4-*b*]indole-2,4,6(3*H*)-trione (5a):

Colourless solid; mp: 190-192 ^{o}C

^{1}H-NMR (400 MHz, $CDCl_3$, ppm): δ 7.98 (d, *J* = 8 Hz, 1H), 7.61 (d, *J* = 3.6 Hz, 2H), 7.39 - 7.43 (m, 1H), 4.46 (s, 3H), 4.12 (s, 3H), 3.81 (s, 3H), 3.48 (s, 3H).

^{13}C-NMR (100 MHz, $CDCl_3$, ppm): δ 157.5, 155.0, 151.7, 139.8, 130.5, 129.4, 126.9, 123.7, 121.9, 120.4, 116.1, 113.6, 111.1, 46.7, 40.1, 37.2, 33.7.

HRMS calcd. for $C_{17}H_{16}N_4O_3$ $[M + H]^+$: 325.1301; found: 325.1298

1,3-Diethyl-5,7-dimethyl-5,7-dihydro-1*H*-pyrimido[4',5':5,6]pyrido[3,4-*b*]indole-2,4,6(3*H*)-trione (5b):

White solid; mp: 242-244 ^{o}C

^{1}H-NMR (400 MHz, $CDCl_3$, ppm): δ 7.94 (d, *J* = 8 Hz, 1H), 7.60 (d, *J* = 3.6 Hz, 2H), 7.39 - 7.42 (m, 1H), 4.55 (q, *J* = 6.8 Hz, 2H), 4.45 (s, 3H), 4.12 (s, 3H), 4.12 (q, *J* = 6.8 Hz, 2H), 1.32 (t, *J* = 7.2 Hz, 3H), 1.03 (t, *J* = 6.8 Hz, 3H).

^{13}C-NMR (100 MHz, $CDCl_3$, ppm): δ 157.5, 155.5, 151.6, 140.9, 131.1, 129.5, 127.0, 123.6, 122.0, 120.2, 116.4, 113.4, 111.0, 46.6, 37.2, 33.7, 31.8, 13.1, 13.0.

HRMS calcd. for $C_{19}H_{20}N_4O_3$ $[M + H]^+$: 353.1614; found: 353.1616

5-Ethyl-1,3,7-trimethyl-5,7-dihydro-1*H*-pyrimido[4',5':5,6]pyrido[3,4-*b*]indole-2,4,6(3*H*)-trione (5c):

White solid; mp: 212-214 °C

^{1}H-NMR (400 MHz, $CDCl_3$, ppm): δ 7.97 (d, *J* = 8 Hz, 1H), 7.60 (d, *J* = 3.6 Hz, 2H), 7.38 - 7.42 (m, 1H), 4.83 (q, *J* = 6.8 Hz, 2H), 4.46 (s, 3H), 3.80 (s, 3H), 3.49 (s, 3H), 1.47 (t, *J* = 7.2 Hz, 3H).

^{13}C-NMR (100 MHz, $CDCl_3$, ppm): δ 157.3, 155.0, 152.3, 140.9, 131.5, 130.8, 126.9, 123.8, 121.9, 120.3, 114.3, 112.9, 111.0, 41.4, 40.2, 31.9, 28.8, 15.2.

HRMS calcd. for $C_{18}H_{18}N_4O_3$ $[M + H]^+$: 339.1457; found:339.1458

3,5-Diethyl-1,7-dimethyl-5,7-dihydro-1*H*-pyrimido[4',5':5,6]pyrido[3,4-*b*]indole-2,4,6(3*H*)-trione (5d):

Colourless solid; mp: 182-184 °C

^{1}H-NMR (400 MHz, $CDCl_3$, ppm): δ 7.93 (d, *J* = 8.4 Hz, 1H), 7.59 (d, *J* = 4 Hz, 2H), 7.38 - 7.42 (m, 1H), 4.83 (q, *J* = 6.8 Hz, 2H), 4.55 (q, *J* = 7.2 Hz, 2H), 4.45 (s, 3H), 3.47 (s, 3H), 1.47 (t, *J* = 6.8 Hz, 3H), 1.02 (t, *J* = 6.8 Hz, 3H).

^{13}C-NMR (100 MHz, $CDCl_3$, ppm): δ 157.5, 155.2, 151.9, 140.9, 131.2, 129.5, 127.0, 123.5, 122.0, 120.2, 115.7, 113.4, 111.1, 46.5, 41.4, 31.9, 28.7, 15.2, 13.2.

HRMS calcd. for $C_{19}H_{20}N_4O_3$ $[M + H]^+$: 353.1614; found: 353.1618

3-Ethyl-1,5,7-trimethyl-5,7-dihydro-1*H*-pyrimido[4',5':5,6]pyrido[3,4-*b*]indole-2,4,6(3*H*)-trione (5e):

Colourless solid; mp: 186-188 °C

^{1}H-NMR (400 MHz, $CDCl_3$, ppm): δ 7.93 (d, *J* = 8.4 Hz, 1H), 7.60 (d, *J* = 4 Hz, 2H), 7.38 - 7.42 (m, 1H), 4.55 (q, *J* = 6.8 Hz, 2H), 4.45 (s, 3H), 4.13 (s, 3H), 3.46 (s, 3H), 1.03 (t, *J* = 6.8 Hz, 3H).

^{13}C-NMR (100 MHz, $CDCl_3$, ppm): δ 158.0, 155.5, 152.0, 140.9, 131.1, 129.4, 127.0, 123.5, 122.0, 120.2, 116.2, 113.4, 111.1, 46.9, 33.6, 31.9, 28.6, 13.1.

HRMS calcd. for $C_{18}H_{18}N_4O_3$ $[M + H]^+$: 339.1457; found: 339.1457

7-Ethyl-1,3,5-trimethyl-5,7-dihydro-1*H*-pyrimido[4',5':5,6]pyrido[3,4-*b*]indole-2,4,6(3*H*)-trione (5f):

White solid; mp: 188-190 °C

^{1}H-NMR (400 MHz, $CDCl_3$, ppm): δ 7.98 (d, *J* = 8.4 Hz, 1H), 7.57 - 7.64 (m, 2H), 7.38 - 7.42 (m, 1H), 5.03 (q, *J* = 7.2 Hz, 2H), 4.12 (s, 3H), 3.81 (s, 3H), 3.49 (s, 3H), 1.52 (t, *J* = 7.2 Hz, 3H).

^{13}C-NMR (100 MHz, $CDCl_3$, ppm): δ 158.2, 155.1, 152.5, 139.9, 130.8, 126.8, 123.9, 121.8, 120.4, 114.9, 113.0, 111.1, 40.1, 33.9, 28.7, 15.9.

HRMS calcd. for $C_{18}H_{18}N_4O_3$ $[M + H]^+$: 339.1457; found: 339.1460

5,7-Diethyl-1,3-dimethyl-5,7-dihydro-1*H*-pyrimido[4',5':5,6]pyrido[3,4-*b*]indole-2,4,6(3*H*)-trione (5g):

White solid; mp: 165-167 °C

^{1}H-NMR (400 MHz, $CDCl_3$, ppm): δ 7.97 (d, *J* = 8 Hz, 1H), 7.56 - 7.64 (m, 2H), 7.37 - 7.41 (m, 1H), 5.02 (q, *J* = 7.2 Hz, 2H), 4.83 (q, *J* = 6.8 Hz, 2H), 3.80 (s, 3H), 3.49 (s, 3H), 1.52 (t, *J* = 7.2 Hz, 3H), 1.48 (t, *J* = 6.8 Hz, 3H).

^{13}C-NMR (100 MHz, $CDCl_3$, ppm): δ 157.3, 154.6, 152.4, 139.8, 130.9, 130.7, 126.8, 123.9, 121.8, 120.4, 114.3, 113.0, 111.1, 41.5, 40.2, 40.1, 28.8, 16.0, 15.2.

HRMS calcd. for $C_{19}H_{20}N_4O_3$ $[M + H]^+$: 353.1614; found: 353.1612

1,3,7-Triethyl-5-methyl-5,7-dihydro-1*H*-pyrimido[4',5':5,6]pyrido[3,4-*b*]indole-2,4,6(3*H*)-trione (5h):

White solid; mp: 172-174 ^oC

^{1}H-NMR (400 MHz, $CDCl_3$, ppm): δ 7.94 (d, *J* = 8 Hz, 1H), 7.56 - 7.63 (m, 2H), 7.37 - 7.41 (m, 1H), 5.01 (q, *J* = 7.2 Hz, 2H), 4.54 (q, *J* = 6.8 Hz, 2H), 4.13 (s, 3H), 4.12 (q, *J* = 6.8 Hz, 2H), 1.52 (t, *J* = 7.2 Hz, 3H), 1.31 (t, *J* = 7.2 Hz, 3H), 1.05 (t, *J* = 6.8 Hz, 3H).

^{13}C-NMR (100 MHz, $CDCl_3$, ppm): δ 157.5, 155.0, 151.7, 139.8, 130.5, 129.4, 126.9, 123.7, 121.9, 120.4, 116.4, 113.6, 111.1, 46.7, 40.7, 37.2, 33.7, 16.0, 13.1, 13.0.

HRMS calcd. for $C_{20}H_{22}N_4O_3$ $[M + H]^+$: 367.1770; found: 367.1768

7-Benzyl-1,3,5-triethyl-5,7-dihydro-1*H*-pyrimido[4',5':5,6]pyrido[3,4-*b*]indole-2,4,6(3*H*)-trione (5i):

White solid; mp: 138-140 ^oC

^{1}H-NMR (400 MHz, $CDCl_3$, ppm): δ 7.88 (d, *J* = 8 Hz, 1H), 7.50 (d, *J* = 8.4 Hz, 1H), 7.44 (t, *J* = 8.4 Hz, 1H), 7.30 (t, *J* = 7.2 Hz, 1H), 7.15 - 7.24 (m, 5H), 6.23 (s, 2H), 4.76 (q, *J* = 6.8 Hz, 2H), 4.47 (q, *J* = 6.8 Hz, 2H), 4.07 (q, *J* = 7.2 Hz, 2H), 1.39 (t, *J* = 6.8 Hz, 3H), 1.25 (t, *J* = 7.2 Hz, 3H), 0.98 (t, *J* = 6.8 Hz, 3H).

^{13}C-NMR (100 MHz, $CDCl_3$, ppm): δ 157.0, 154.9, 151.6, 140.4, 137.4, 130.8, 129.4, 128.7, 127.6, 127.1, 127.0, 123.6, 122.1, 120.5, 116.2, 114.0, 111.9, 48.2, 46.8, 41.6, 37.3, 15.2, 13.1, 13.0.

HRMS calcd. for $C_{26}H_{26}N_4O_3$ $[M + H]^+$: 443.2083; found: 443.2088

7-Benzyl-1,3-diethyl-5-methyl-5,7-dihydro-1*H*-pyrimido[4',5':5,6]pyrido[3,4-*b*]indole-2,4,6(3*H*)-trione (5j):

White solid; mp: 156-158 °C

^{1}H-NMR (400 MHz, $CDCl_3$, ppm): δ 7.98 (d, *J* = 8.4 Hz, 1H), 7.60 (d, *J* = 8 Hz, 1H), 7.54 (t, *J* = 8 Hz, 1H), 7.40 (t, *J* = 7.6 Hz, 1H), 7.24 - 7.42 (m, 5H), 6.27 (s, 2H), 4.57 (q, *J* = 7.2 Hz, 2H), 4.15 (s, 3H), 4.10 - 4.20 (m, 2H), 1.34 (t, *J* = 7.2 Hz, 3H), 1.08 (t, *J* = 6.8 Hz, 3H).

^{13}C-NMR (100 MHz, $CDCl_3$, ppm): δ 157.5, 155.2, 151.6, 140.4, 137.3, 130.6, 129.3, 128.8, 127.6, 127.2, 126.9, 123.7, 122.2, 120.5, 116.7, 114.0, 111.9, 48.2, 46.7, 37.2, 33.8, 13.1, 13.0.

HRMS calcd. for $C_{25}H_{24}N_4O_3$ $[M + H]^+$: 429.1927; found: 429.1930

7-Butyl-1,3-diethyl-5-methyl-5,7-dihydro-1*H*-pyrimido[4',5':5,6]pyrido[3,4-*b*]indole-2,4,6(3*H*)-trione (5k):

White solid; mp: 115-117 °C

^{1}H-NMR (400 MHz, $CDCl_3$, ppm): δ 7.87 (d, *J* = 8.4 Hz, 1H), 7.48-7.55 (m, 2H), 7.31 - 7.33 (m, 1H), 4.86 (t, *J* = 7.6 Hz, 2H), 4.47 (q, *J* = 6.8 Hz, 2H), 4.05 (q, *J* = 6.8 Hz, 2H), 4.05 (s, 3H), 1.83 – 1.84 (m, 2H), 1.36 – 1.42 (m, 2H), 1.24 (t, *J* = 7.2 Hz, 3H), 0.97 (t, *J* = 6.8 Hz, 3H), 0.91 (t, *J* = 7.2 Hz, 3H).

^{13}C-NMR (100 MHz, $CDCl_3$, ppm): δ 157.5, 155.1, 151.7, 140.1, 130.7, 129.4, 126.8, 123.6, 121.8, 120.2, 116.4, 113.5, 111.3, 46.7, 44.9, 37.1, 33.7, 33.0, 20.2, 13.9, 13.1, 13.0.

HRMS calcd. for $C_{22}H_{26}N_4O_3$ $[M + H]^+$: 395.2083; found: 395.2083

7-Butyl-1,3,5-triethyl-5,7-dihydro-1*H*-pyrimido[4',5':5,6]pyrido[3,4-*b*]indole-2,4,6(3*H*)-trione (5l):

White solid; mp: 162-164 ^{o}C

^{1}H-NMR (400 MHz, $CDCl_3$, ppm): δ 7.86 (d, *J* = 8.4 Hz, 1H), 7.47 – 7.54 (m, 2H), 7.28 - 7.32 (m, 1H), 4.86 (t, *J* = 7.6 Hz, 2H), 4.75 (q, *J* = 6.8 Hz, 2H), 4.46 (q, *J* = 6.8 Hz, 2H), 4.06 (q, *J* = 7.2 Hz, 2H), 1.78 – 1.86 (m, 2H), 1.41 (t, *J* = 6.8 Hz, 3H), 1.34-1.43 (m, 2H), 1.24 (t, *J* = 7.2 Hz, 3H), 0.97 (t, *J* = 6.8 Hz, 3H), 0.91 (t, *J* = 7.2 Hz, 3H).

^{13}C-NMR (100 MHz, $CDCl_3$, ppm): δ 157.0, 154.7, 151.6, 140.1, 130.9, 129.5, 126.8, 123.6, 121.8, 120.3, 115.9, 113.5, 111.3, 46.7, 44.9, 41.5, 37.2, 33.0, 20.2, 15.2, 13.9, 13.1, 13.0.

HRMS calcd. for $C_{23}H_{28}N_4O_3$ $[M + H]^+$: 409.2240; found: 409.2238

(b) Experimental characterization data of starting material (4):

***N*-(1,3-Dimethyl-2,4-dioxo-1,2,3,4-tetrahydropyrimidin-5-yl)-*N*,1-dimethyl-1*H*-indole-2-carboxamide (4a):**

Colourless gummy gel

^{1}H-NMR (400 MHz, $CDCl_3$, ppm): δ 7.55 (d, *J* = 7.6 Hz, 1H); 7.26-7.35 (m, 3H, indole C3-H and two ArH); 7.10 (t, *J* = 7.2 Hz, 1H); 6.46 (s, 1H, uracil C6-H); 3.89 (s, 3H); 3.42 (s, 3H), 3.37 (s, 3H); 3.32 (s, 3H).

^{13}C-NMR (100 MHz, CDC_{l3}, ppm): δ 165.4, 160.7, 150.9, 141.1, 138.1, 131.9, 126.0, 123.7, 121.8, 120.2, 110.0, 105.3, 37.8, 37.3, 31.3, 28.5.

HRMS calcd. for $C_{17}H_{18}N_4O_3$ $[M + H]^+$: 327.1457; found: 327.1455

***N*-(1,3-Diethyl-2,4-dioxo-1,2,3,4-tetrahydropyrimidin-5-yl)-*N*,1-dimethyl-1*H*-indole-2-carboxamide (4b):**

White solid; mp: 135-137 °C

^{1}H-NMR (400 MHz, CDCl3, ppm): δ 7.53 (d, *J* = 7.2 Hz, 1H); 7.33 (d, *J* = 8.4 Hz, 1H); 7.25-7.29 (m, 2H, indole C3-H and one ArH); 7.10 (t, *J* = 7.2 Hz, 1H), 6.43 (s, 1H, uracil C6-H); 3.91-3.92 (m, 2H, N$\mathbf{CH_2}$$CH_3$), 3.88 (s, 3H); 3.71-3.77 (m, 2H, N$\mathbf{CH_2}$$CH_3$); 3.35 (s, 3H); 1.23-1.31(m, 3H, $NCH_2$$\mathbf{CH_3}$); 1.08 (s, 3H, $NCH_2$$\mathbf{CH_3}$).

^{13}C-NMR (100 MHz, $CDCl_3$, ppm): δ 165.4, 160.2, 149.9, 139.7, 137.9, 132.1, 126.1, 123.6, 121.6, 120.2, 109.9, 105.0, 45.1, 37.3, 37.1, 31.3, 14.3, 12.6.

HRMS calcd. for $C_{19}H_{22}N_4O_3$ $[M + H]^+$: 355.1770; found: 355.1771

***N*-(1,3-Dimethyl-2,4-dioxo-1,2,3,4-tetrahydropyrimidin-5-yl)-*N*-ethyl-1-methyl-1*H*-indole-2-carboxamide (4c):**

White solid; mp: 154-156 °C

^{1}H-NMR (400 MHz, $CDCl_3$, ppm): δ 7.55 (d, *J* = 7.6 Hz, 1H); 7.34 (d, *J* = 8.0 Hz, 1H); 7.27-7.29 (m, 2H, one ArH and one indole C3-H), 7.10 (t, *J* = 7.2 Hz, 1H); 6.27 (s, 1H, uracil C6-H); 3.89 (s, 3H); 3.79 (brs, 2H, N$\mathbf{CH_2}$$CH_3$); 3.40 (s, 3H); 3.27 (s, 3H); 1.23 (t, *J* = 6.8 Hz, 3H, N$\mathbf{CH_2}$$CH_3$).

^{13}C-NMR (100 MHz, $CDCl_3$, ppm): δ 165.0, 161.0, 150.9, 141.1, 138.0, 132.5, 126.1, 123.5, 121.7, 120.1, 110.0, 104.7, 44.7, 37.4, 31.3, 28.5, 13.1.

HRMS calcd. for $C_{18}H_{20}N_4O_3$ $[M + H]^+$: 341.1614; found: 341.1619

***N*-Ethyl-*N*-(3-ethyl-1-methyl-2,4-dioxo-1,2,3,4-tetrahydropyrimidin-5-yl)-1-methyl-1*H*-indole-2-carboxamide (4d):**

Colourless gummy gel

^{1}H-NMR (400 MHz, $CDCl_3$, ppm): δ 7.53 (d, *J* = 7.6 Hz, 1H); 7.33 (d, *J* = 8 Hz, 1H); 7.25-7.28 (m, 2H, one ArH and one indole C3-H); 7.09 (t, *J* = 7.2 Hz, 1H); 6.40 (s, 1H, uracil C6-H); 3.86-3.91 (m, 4H, one N-Me and 1H of N$\mathbf{CH_2}$$CH_3$); 3.70-3.80 (m, 3H, 1H of N$\mathbf{CH_2}$$CH_3$ and 2H of N$\mathbf{CH_2}$$CH_3$); 3.26 (s, 3H, N-Me); 1.23 (t, *J* = 6.8 Hz, 6H, two $NCH_2$$\mathbf{CH_3}$).

^{13}C-NMR (100 MHz, $CDCl_3$, ppm): δ 165.0, 160.9, 150.4, 140.3, 138.0, 132.5, 126.1, 123.5, 121.6, 120.1, 109.9, 104.8, 45.2, 44.4, 31.3, 28.5, 14.4, 13.2.

HRMS calcd. for $C_{19}H_{22}N_4O_3$ $[M + H]^+$: 355.1770; found: 355.1767

***N*-(3-Ethyl-1-methyl-2,4-dioxo-1,2,3,4-tetrahydropyrimidin-5-yl)-*N*,1-dimethyl-1*H*-indole-2-carboxamide (4e):**

Colourless gummy gel

^{1}H-NMR (400 MHz, $CDCl_3$, ppm): δ 7.53 (d, *J* = 8 Hz, 1H); 7.33 (d, *J* = 8 Hz, 1H); 7.25-7.29 (m, 2H, one ArH and one indole C3-H); 7.10 (t, *J* = 7.6 Hz, 1H); 6.44 (s, 1H, uracil C6-H); 3.77 (s, 3H, N-Me); 3.77 (q, *J* = 7.2 Hz, 2H, N$\mathbf{CH_2}$$CH_3$); 3.33 (s, 3H, N-Me); 3.27 (s, 3H, N-Me); 1.26 (t, *J* = 7.2 Hz, 3H, $NCH_2$$\mathbf{CH_3}$).

^{13}C-NMR (100 MHz, $CDCl_3$, ppm): δ 165.5, 160.6, 150.4, 140.1, 138.0, 132.0, 126.1, 123.6, 121.7, 120.2, 110.0, 105.3, 45.2, 37.5, 31.3, 28.4, 14.3.

HRMS calcd. for $C_{18}H_{20}N_4O_3$ $[M + H]^+$: 341.1614; found: 341.1617

***N*-(1,3-Dimethyl-2,4-dioxo-1,2,3,4-tetrahydropyrimidin-5-yl)-1-ethyl-*N*-methyl-1*H*-indole-2-carboxamide (4f):**

Colourless gummy gel

^{1}H-NMR (400 MHz, $CDCl_3$, ppm): δ 7.57 (d, *J* = 7.6 Hz, 1H); 7.37 (d, *J* = 8.4 Hz, 1H); 7.28 (d, *J* = 7.6 Hz, 1H); 7.26 (s, 1H, indole C3-H); 7.11 (t, *J* = 7.6 Hz, 1H); 6.48 (s, 1H, uracil C6-H); 4.40 (q, *J* = 7.2 Hz, 2H, N$\mathbf{CH_2}$$CH_3$) 3.39 (s, 3H, N-Me); 3.33 (s, 3H, N-Me); 3.31 (s, 3H, N-Me); 1.46 (t, *J* = 7.2 Hz, 3H, N$CH_2$$\mathbf{CH_3}$).

^{13}C-NMR (100 MHz, $CDCl_3$, ppm): δ 165.4, 160.6, 150.9, 141.2, 137.0, 131.0, 126.3, 123.6, 121.9, 120.1, 110.2, 105.5, 39.7, 38.1, 37.4, 28.5, 15.6.

HRMS calcd. for $C_{18}H_{20}N_4O_3$ $[M + H]^+$: 341.1614; found: 341.1611

_N_-(1,3-Dimethyl-2,4-dioxo-1,2,3,4-tetrahydropyrimidin-5-yl)-_N_,1-diethyl-1_H_-indole-2-carboxamide (4g):

Colourless solid mp: 85-87 ^{o}C

^{1}H-NMR (400 MHz, $CDCl_3$, ppm): δ 7.56 (d, *J* = 6.8 Hz, 1H); 7.36 (d, *J* = 8.0 Hz, 1H); 7.22-7.28 (m, 2H, one ArH and one indole C3-H); 7.09 (t, *J* = 7.2 Hz, 1H); 6.45 (s, 1H, uracil C6-H); 4.38 (d, *J* = 6.8 Hz, 2H, N$\mathbf{CH_2}$$CH_3$) 3.70-3.82 (m, 2H, N$\mathbf{CH_2}$$CH_3$); 3.38 (s, 3H, N-Me); 3.30 (s, 3H, N-Me); 1.44-1.48 (m, 3H, N$CH_2$$\mathbf{CH_3}$); 1.20-1.1.26 (m, 3H, N$CH_2$$\mathbf{CH_3}$).

^{13}C-NMR (100 MHz, $CDCl_3$, ppm): δ 165.4, 160.5, 150.4, 140.1, 136.9, 131.1, 126.3, 123.6, 121.8, 120.1, 110.2, 105.5, 45.2, 39.7, 37.2, 28.4, 15.6, 14.3.

HRMS calcd. for $C_{19}H_{22}N_4O_3$ $[M + H]^+$: 355.1770; found: 355.1773

N-(1,3-Diethyl-2,4-dioxo-1,2,3,4-tetrahydropyrimidin-5-yl)-1-ethyl-N-methyl-1H-indole-2-carboxamide (4h):

White solid; mp:126-128 °C

^{1}H-NMR (400 MHz, $CDCl_3$, ppm): δ 7.53 (d, *J* = 7.2 Hz, 1H); 7.36 (d, *J* = 8.0 Hz, 1H); 7.22-7.28 (m, 2H, one ArH and one indole C3-H); 7.09 (t, *J* = 7.2 Hz, 1H); 6.44 (s, 1H, uracil C6-H); 4.39 (q, *J* = 6.8 Hz, 2H, N$\mathbf{CH_2}$$CH_3$); 3.93 (d, *J* = 6.4 Hz, 2H, N$\mathbf{CH_2}$$CH_3$); 3.75 (d, *J* = 6.8 Hz, 2H, N$\mathbf{CH_2}$$CH_3$); 3.34 (s, 3H, N-Me); 1.45 (t, *J* = 7.2 Hz, 3H, $NCH_2$$\mathbf{CH_3}$); 1.22 (s, 3H, $NCH_2$$\mathbf{CH_3}$); 1.12 (s, 3H, $NCH_2$$\mathbf{CH_3}$).

^{13}C-NMR (100 MHz, $CDCl_3$, ppm): δ 165.4, 160.2, 150.0, 139.9, 136.8, 131.2, 126.4, 123.5, 121.8, 120.1, 110.1, 105.2, 45.1, 39.6, 37.3, 37.1, 15.6, 14.3, 12.7.

HRMS calcd. for $C_{20}H_{24}N_4O_3$ $[M + H]^+$: 369.1927; found: 369. 1923

1-Benzyl-N-(1,3-diethyl-2,4-dioxo-1,2,3,4-tetrahydropyrimidin-5-yl)-N-ethyl-1H-indole-2-carboxamide (4i):

White solid; mp:132-134 °C

^{1}H-NMR (400 MHz, $CDCl_3$, ppm): δ 7.51 (d, *J* = 8.4 Hz, 2H); 7.22-7.34 (m, 7H, six ArH and one indole C3-H); 7.11 (t, *J* = 7.6 Hz, 1H); 6.40 (s, 1H, uracil C6-H); 5.43 (s, 2H); 4.18 (s, 1H, N$\mathbf{CH_2}$$CH_3$); 3.99 (q, *J* = 7.2 Hz, 2H, N$\mathbf{CH_2}$$CH_3$); 3.42 (s, 1H, N$\mathbf{CH_2}$$CH_3$); 3.27 (s, 1H, N$\mathbf{CH_2}$$CH_3$); 3.12 (s, 1H, N$\mathbf{CH_2}$$CH_3$); 1.99 (t, *J* = 7.2 Hz, 3H, $NCH_2$$\mathbf{CH_3}$); 1.12 (t, *J* = 7.2 Hz, 3H, $NCH_2$$\mathbf{CH_3}$); 0.88 (s, 3H, $NCH_2$$\mathbf{CH_3}$).

^{13}C-NMR (100 MHz, $CDCl_3$, ppm): δ 163.9, 160.0, 149.3, 140.2, 138.9, 137.1, 130.9, 128.2, 127.9, 127.2, 125.9, 123.3, 121.4, 119.8, 116.8, 109.3, 104.2, 46.4, 44.2, 41.8, 36.5, 13.7, 12.2, 11.9.

HRMS calcd. for $C_{26}H_{29}N_4O_3$ $[M + H]^+$: 444.2161; found: 444.2159

1-Benzyl-*N*-(1,3-diethyl-2,4-dioxo-1,2,3,4-tetrahydropyrimidin-5-yl)-*N*-methyl-1*H*-indole-2-carboxamide (4j):

White solid; mp: 116-118 ^oC

^1H-NMR (400 MHz, $CDCl_3$, ppm): δ 7.54 (d, *J* = 7.6 Hz, 1H); 7.48 (d, *J* = 8.4 Hz, 1H); 7.26-7.35 (m, 7H, six ArH and one indole C3-H); 7.13 (t, *J* = 7.6 Hz, 1H); 6.43 (s, 1H, uracil C6-H); 5.70 (s, 2H); 4.38 (s, 1H, N$\mathbf{CH_2}$$CH_3$); 4.02 (q, *J* = 6.8 Hz, 2H, N$\mathbf{CH_2}$$CH_3$); 3.44 (s, 1H, N$\mathbf{CH_2}$$CH_3$); 3.26 (s, 3H, N$\mathbf{CH_3}$); 1.22 (t, *J* = 6.8 Hz, 3H, $NCH_2$$\mathbf{CH_3}$); 0.93 (s, 3H, $NCH_2$$\mathbf{CH_3}$).

^{13}C-NMR (100 MHz, $CDCl_3$, ppm): δ 164.8, 160.3, 149.8, 140.1, 139.1, 137.6, 131.2, 128.7, 127.9, 127.7, 126.4, 123.9, 121.9, 120.4, 119.4, 110.0, 105.1, 47.1, 44.8, 36.9, 36.2, 14.2, 12.8.

HRMS calcd. for $C_{25}H_{26}N_4O_3$ $[M + H]^+$: 431.2083; found: 431.2082

1-Butyl-*N*-(1,3-diethyl-2,4-dioxo-1,2,3,4-tetrahydropyrimidin-5-yl)-*N*-methyl-1*H*-indole-2-carboxamide (4k):

Colourless gummy liquid

^1H-NMR (400 MHz, $CDCl_3$, ppm): δ 7.55 (d, *J* = 7.2 Hz, 1H); 7.37 (d, *J* = 8.0 Hz, 1H); 7.26 (d, *J* = 8.0 Hz, 1H); 7.20 (s, 1H, C3-H of indole); 7.11(t, *J* = 7.2 Hz, 1H); 6.49 (s, 1H, C6-H of uracil); 4.35 (s, 2H, N$\mathbf{CH_2}$$CH_3$); 3.98 (d, *J* = 6.8 Hz, 2H, may N$\mathbf{CH_2}$$CH_2$ or N$\mathbf{CH_2}$$CH_3$); 3.75 (d, *J* = 6.8 Hz, 2H, may N**CH2**CH_2 or N$\mathbf{CH_2}$$CH_3$); 3.36 (s, 3H, N$\mathbf{CH_3}$); 1.85 (quintet, *J* = 7.6 Hz, 2H, $NCH_2$$\mathbf{CH_2}$$CH_2$); 1.29-1.45 (m, 2H, -$CH_2$$\mathbf{CH_2}$$CH_3$); 1.22 (s, 3H, $NCH_2$$\mathbf{CH_3}$); 1.64 (s, 3H, $NCH_2$$\mathbf{CH_3}$); 0.98 (t, *J* = 7.6 Hz, 3H, -$CH_2$$\mathbf{CH_3}$).

^{13}C-NMR (100 MHz, $CDCl_3$, ppm): δ 165.3, 160.2, 150.0, 139.9, 137.3, 131.2, 126.3, 123.5, 121.8, 120.1, 110.3, 105.3, 45.1, 44.5, 37.2, 37.1, 32.5, 20.3, 14.3, 13.9, 12.7.

HRMS calcd. for $C_{22}H_{28}N_4O_3$ $[M + H]^+$: 397.2240; found: 397.2243

1-Butyl-*N*-(1,3-diethyl-2,4-dioxo-1,2,3,4-tetrahydropyrimidin-5-yl)-*N*-ethyl-1*H*-indole-2-carboxamide (4l):

Colourless solid; mp: 82-84 °C

^{1}H-NMR (400 MHz, $CDCl_3$, ppm): δ 7.45 (d, *J* = 7.2 Hz, 1H); 7.26 (t, *J* = 8.0 Hz, 1H); 7.14-7.19 (m, 1H); 7.01 (t, *J* = 7.2 Hz, 2H); 6.37 (s, 1H, C6-H of uracil); 4.25 (s, 2H, may N**CH₂**CH₂ or N**CH₂**CH₃); 3.89 (d, *J* = 6.4 Hz, 3H, may N**CH₂**CH₂ or N**CH₂**CH₃); 3.67-3.72 (m, 3H, may N**CH₂**CH₂ or N**CH₂**CH₃); 1.76 (quintet, *J* = 7.6 Hz, 2H, NCH₂**CH₂**CH₂); 1.21-1.28 (m, 2H, -CH₂**CH₂**CH₃); 1.16 (t, *J* = 7.2 Hz, 6H, NCH₂**CH₃**); 1.01 (s, 3H, NCH₂**CH₃**); 0.89 (t, *J* = 7.6 Hz, 3H, -CH₂**CH₃**).

^{13}C-NMR (100 MHz, $CDCl_3$, ppm): δ 164.9, 160.5, 150.0, 140.2, 137.2, 131.7, 126.3, 123.3, 121.7, 120.0, 110.2, 104.7, 45.0, 44.5, 44.0, 37.1, 32.5, 20.3, 14.3, 13.9, 13.2, 12.6.

HRMS calcd. for $C_{23}H_{30}N_4O_3$ $[M + H]^+$: 411.2396; found: 411.2391

***N*-(1,3-Diethyl-2,4-dioxo-1,2,3,4-tetrahydropyrimidin-5-yl)-*N*-methyl-1*H*-indole-2-carboxamide (4m):**

White solid; mp: 210-212 °C

^{1}H-NMR (400 MHz, $CDCl_3$, ppm): δ 9.52 (s, 1H, NH); 7.47 (d, *J* = 7.2 Hz, 1H); 7.34 (d, *J* = 8.4 Hz, 1H); 7.28 (s, 1H, C3-H of indole); 7.19 (t, *J* = 8.4 Hz, 1H); 7.02 (t, *J* = 7.2 Hz, 1H); 6.13 (s, 1H, C6-H of uracil); 4.00 (q, *J* = 6.8 Hz, 2H, N**CH₂**CH₃); 3.77(q, *J* = 7.2 Hz, 2H, N**CH₂**CH₃); 3.32 (s, 3H, N**CH₃**); 1.24 (t, *J* = 7.2 Hz, 3H, NCH₂**CH₃**); 1.18 (t, *J* = 6.8 Hz, 3H, NCH₂**CH₃**).

^{13}C-NMR (100 MHz, $CDCl_3$, ppm): δ 163.4, 160.2, 150.3, 141.2, 135.6, 129.1, 127.6, 124.7, 122.1, 120.5, 118.6, 111.9, 105.9, 45.2, 37.8, 37.2, 14.5, 12.8.

HRMS calcd. for $C_{18}H_{20}N_4O_3$ $[M + H]^+$: 341.1614; found: 341.1618

(4) References:

1. D. B. England; A. Padwa *Org. Lett.* **2008,** *10,* 3631.

(5) NMR (^{1}H and ^{13}C) spectra of products (5):

1,3,5,7-Tetramethyl-5,7-dihydro-1*H*-pyrimido[4',5':5,6]pyrido[3,4-*b*]indole-2,4,6(3*H*)-trione (5a):

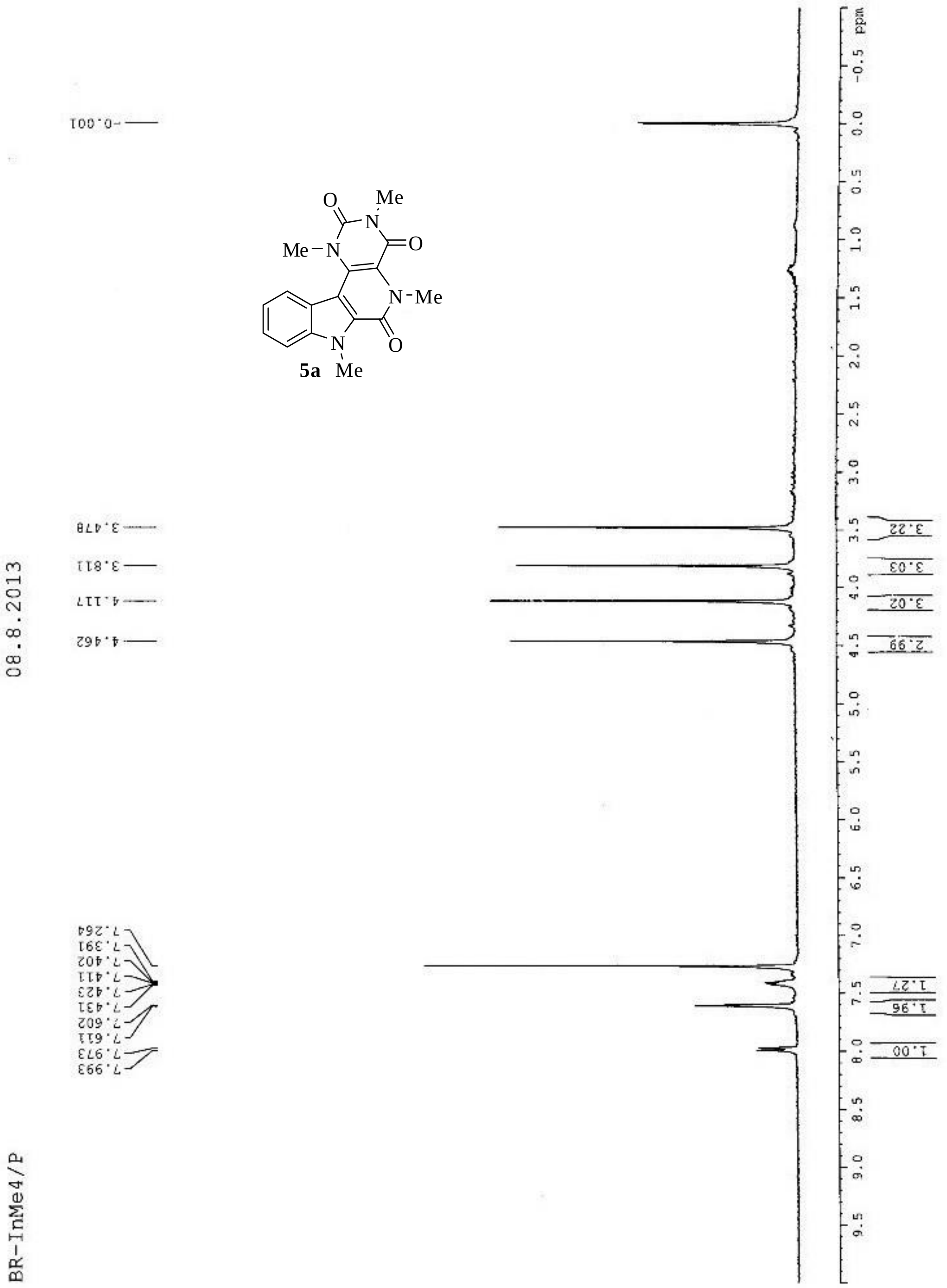

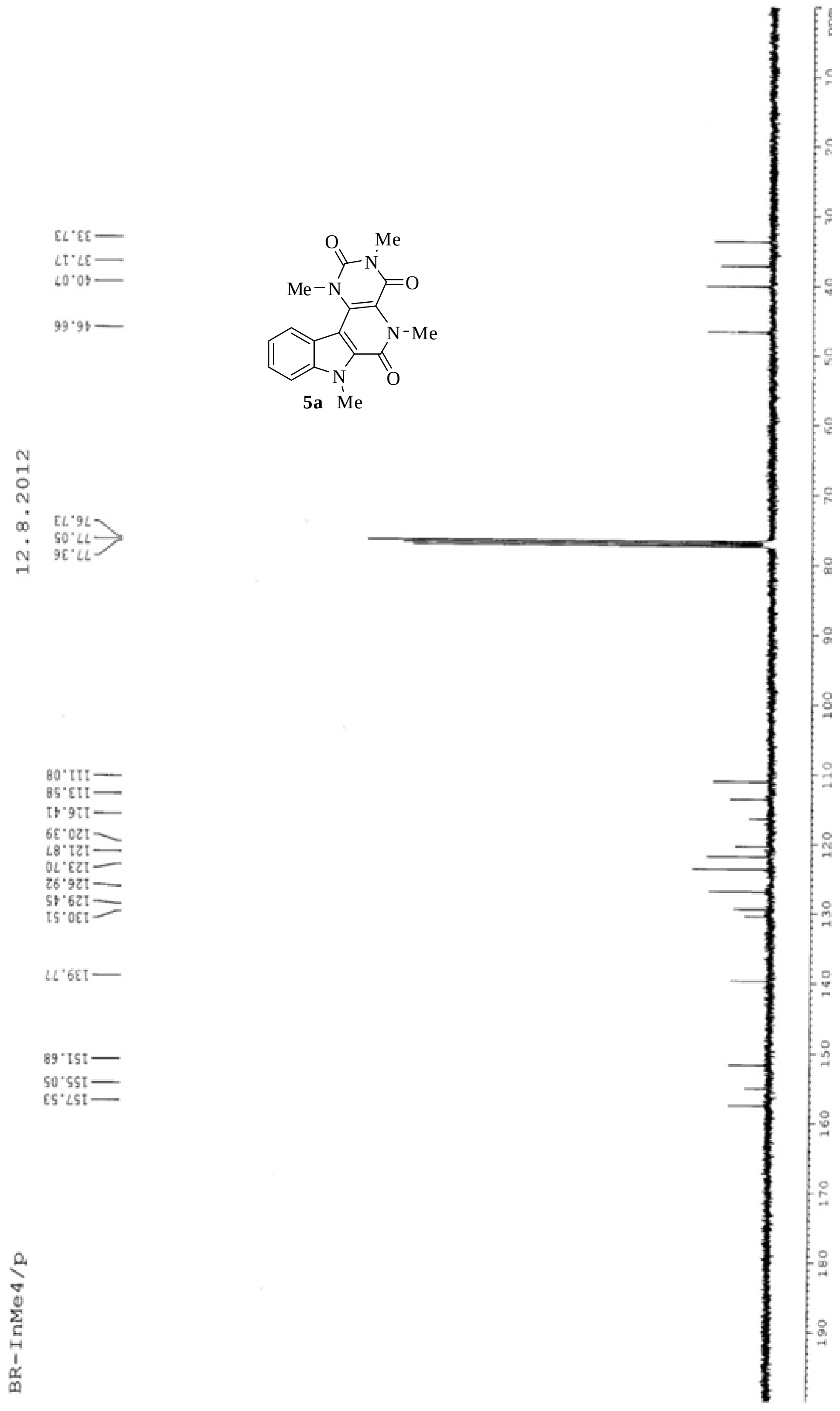
BR-InMe4/p
12.8.2012
157.53
155.05
151.68
139.77
130.51
129.45
126.92
123.70
121.87
120.39
116.41
113.58
111.08
77.36
77.05
76.73
46.66
40.07
37.17
33.73
5a
ppm

1,3-Diethyl-5,7-dimethyl-5,7-dihydro-1*H*-pyrimido[4',5':5,6]pyrido[3,4-*b*]indole-2,4,6(3*H*)-trione (5b):

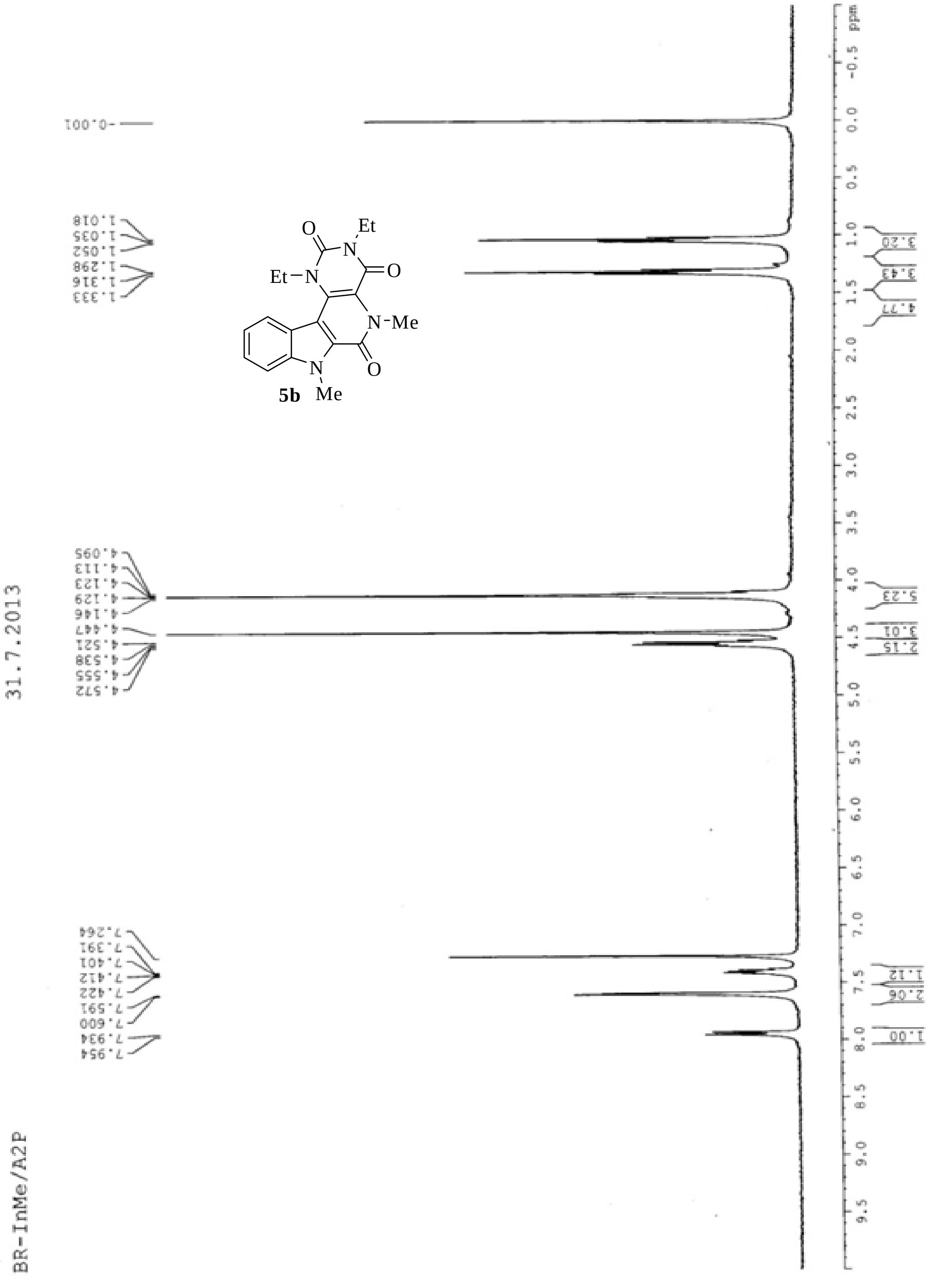

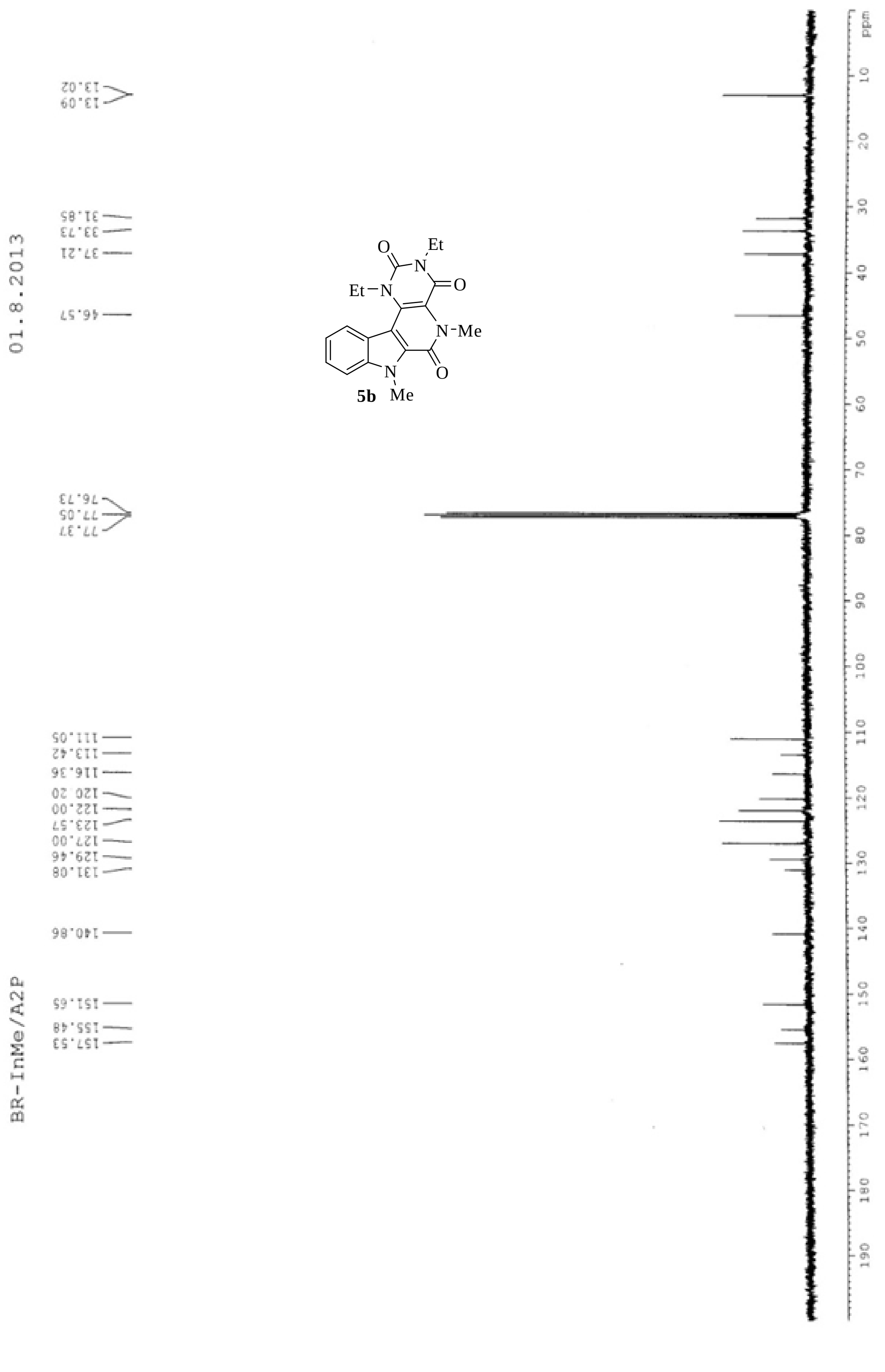
01.8.2013
BR-InMe/A2P
13.02
13.09
31.85
33.73
37.21
46.57
76.73
77.05
77.37
111.05
113.42
116.36
120.20
122.00
123.57
127.00
129.46
131.08
140.86
151.65
155.48
157.53
ppm
5b

5-Ethyl-1,3,7-trimethyl-5,7-dihydro-1*H*-pyrimido[4',5':5,6]pyrido[3,4-*b*]indole-2,4,6(3*H*)-trione (5c):

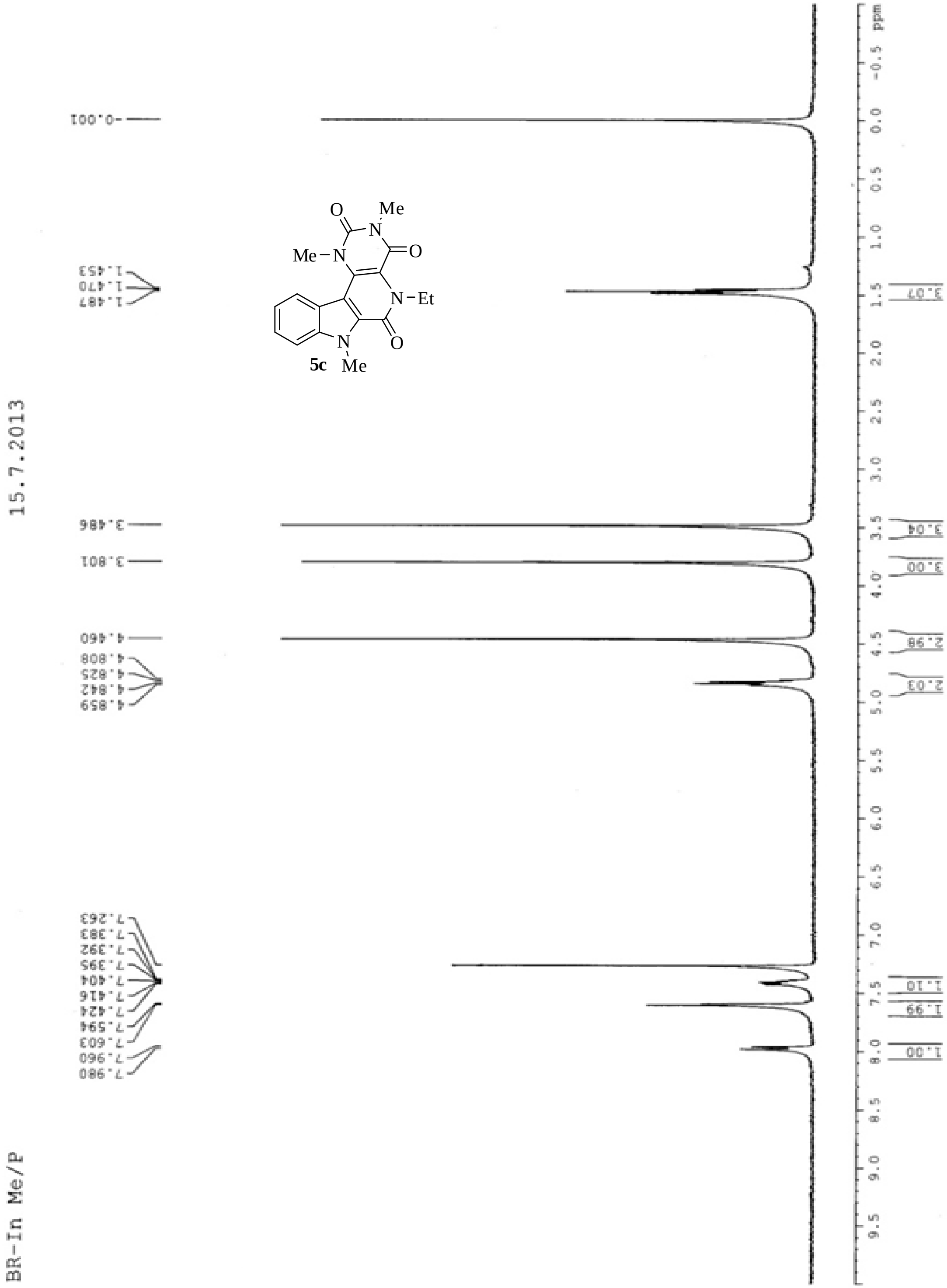

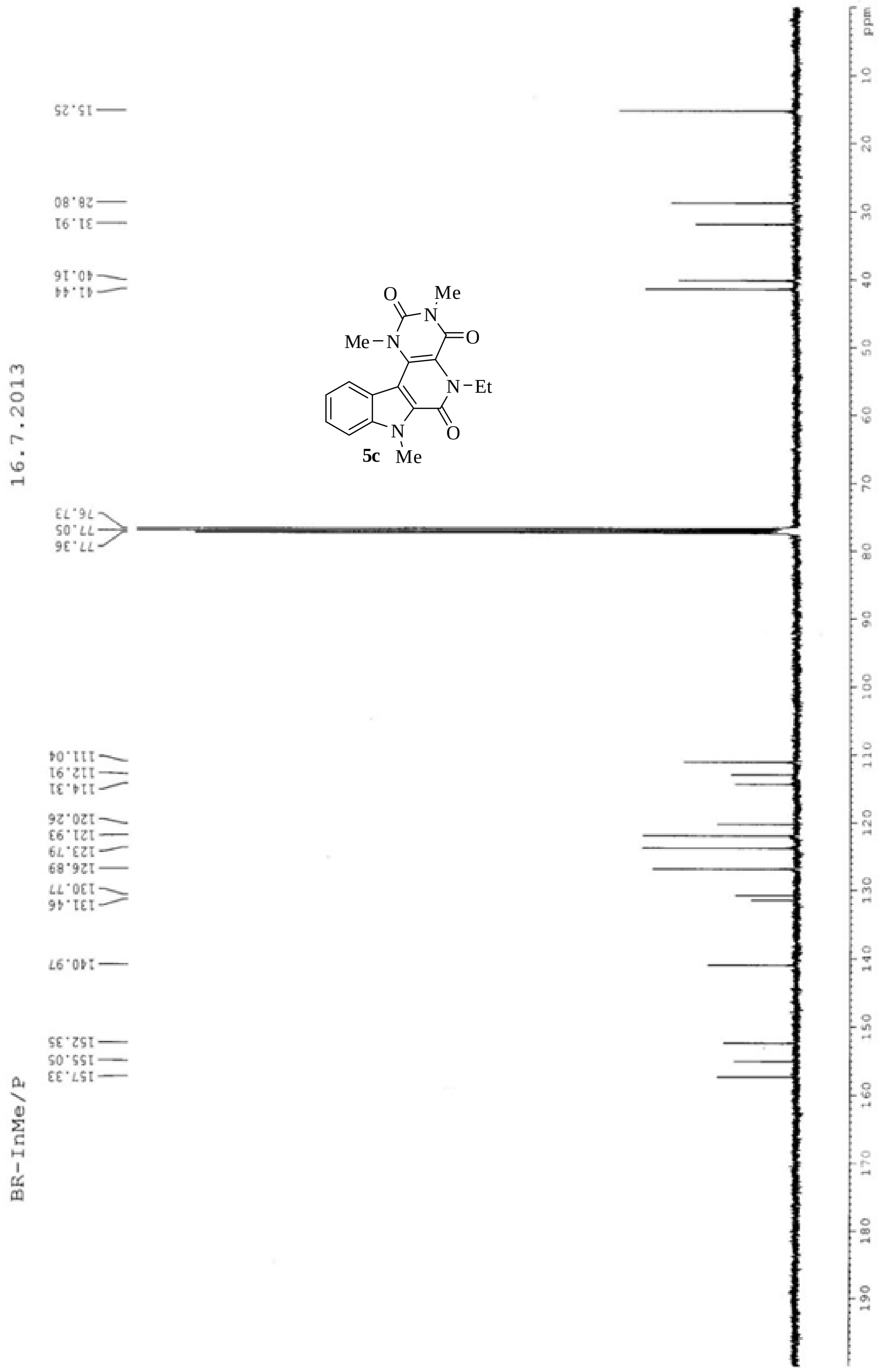
16.7.2013
BR-InMe/P
15.25
28.80
31.91
40.16
41.44
76.73
77.05
77.36
111.04
112.91
114.31
120.26
121.93
123.79
126.89
130.77
131.46
140.97
152.35
155.05
157.33
ppm
5c

3,5-Diethyl-1,7-dimethyl-5,7-dihydro-1*H*-pyrimido[4',5':5,6]pyrido[3,4-*b*]indole-2,4,6(3*H*)-trione (5d):

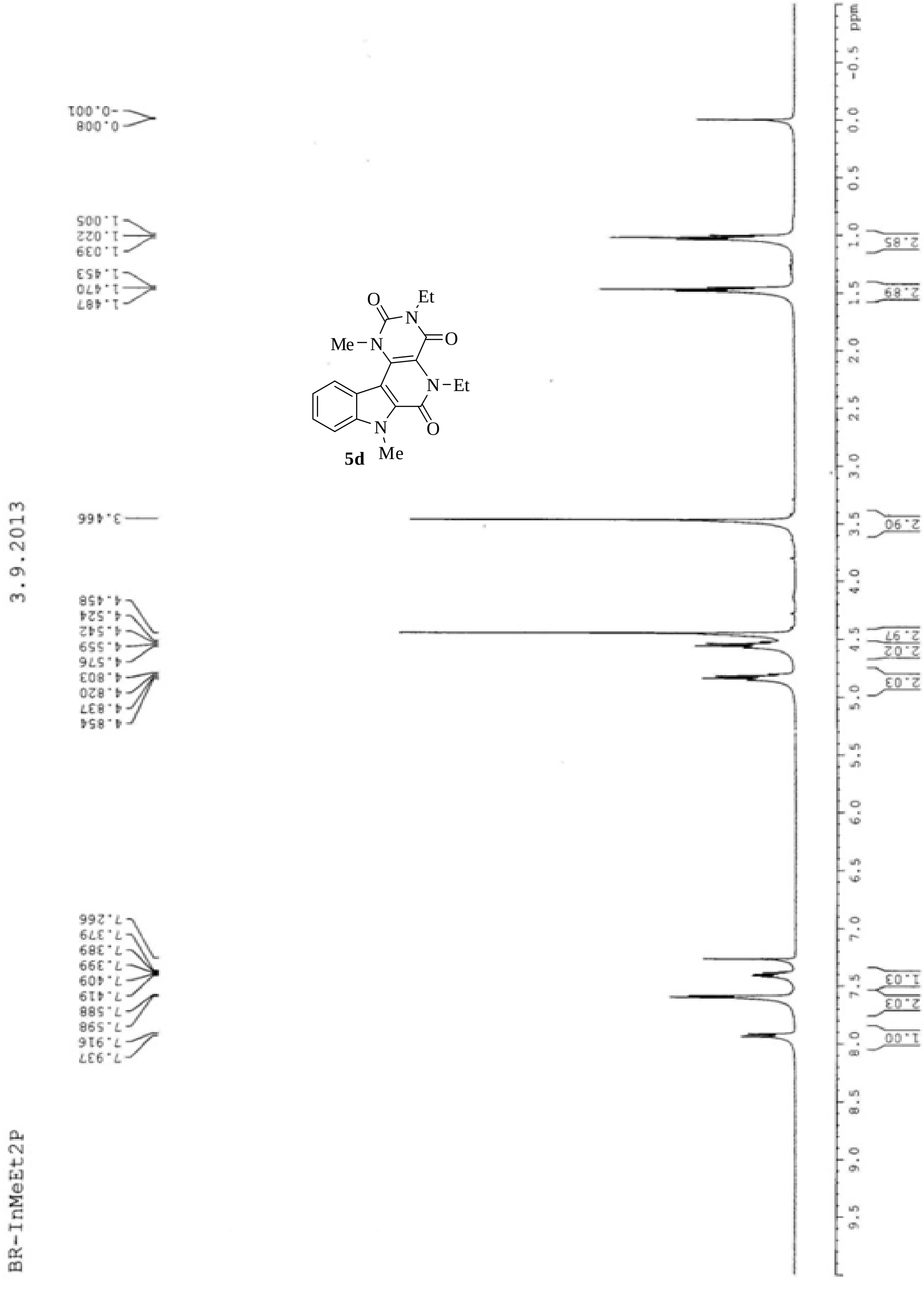

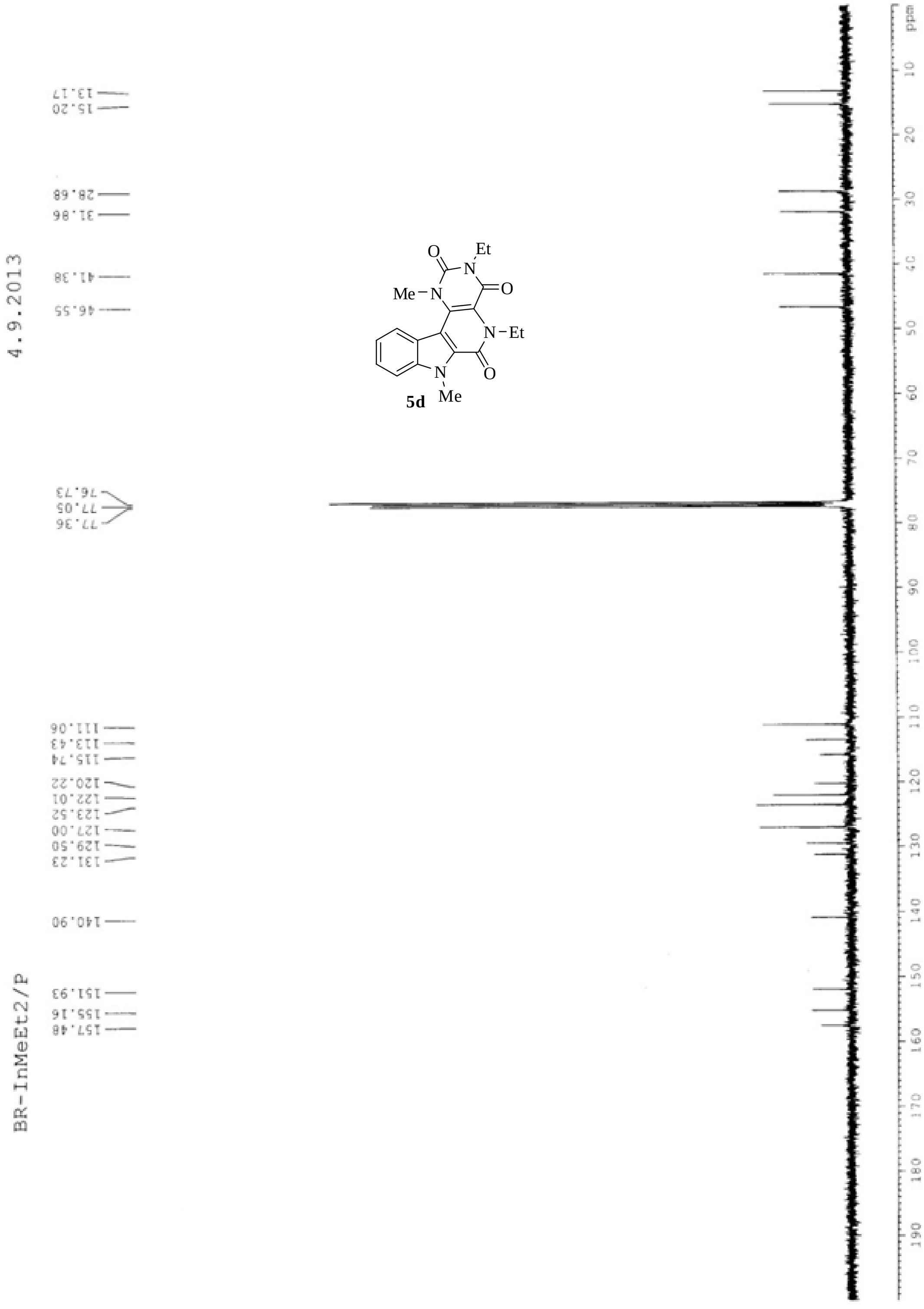
BR-InMeEt2/P
4.9.2013
157.48
155.16
151.93
140.90
131.23
129.50
127.00
123.52
122.01
120.22
115.74
113.43
111.06
77.36
77.05
76.73
46.55
41.38
31.86
28.68
15.20
13.17
5d
ppm
10
20
30
40
50
60
70
80
90
100
110
120
130
140
150
160
170
180
190

3-Ethyl-1,5,7-trimethyl-5,7-dihydro-1*H*-pyrimido[4',5':5,6]pyrido[3,4-*b*]indole-2,4,6(3*H*)-trione (5e):

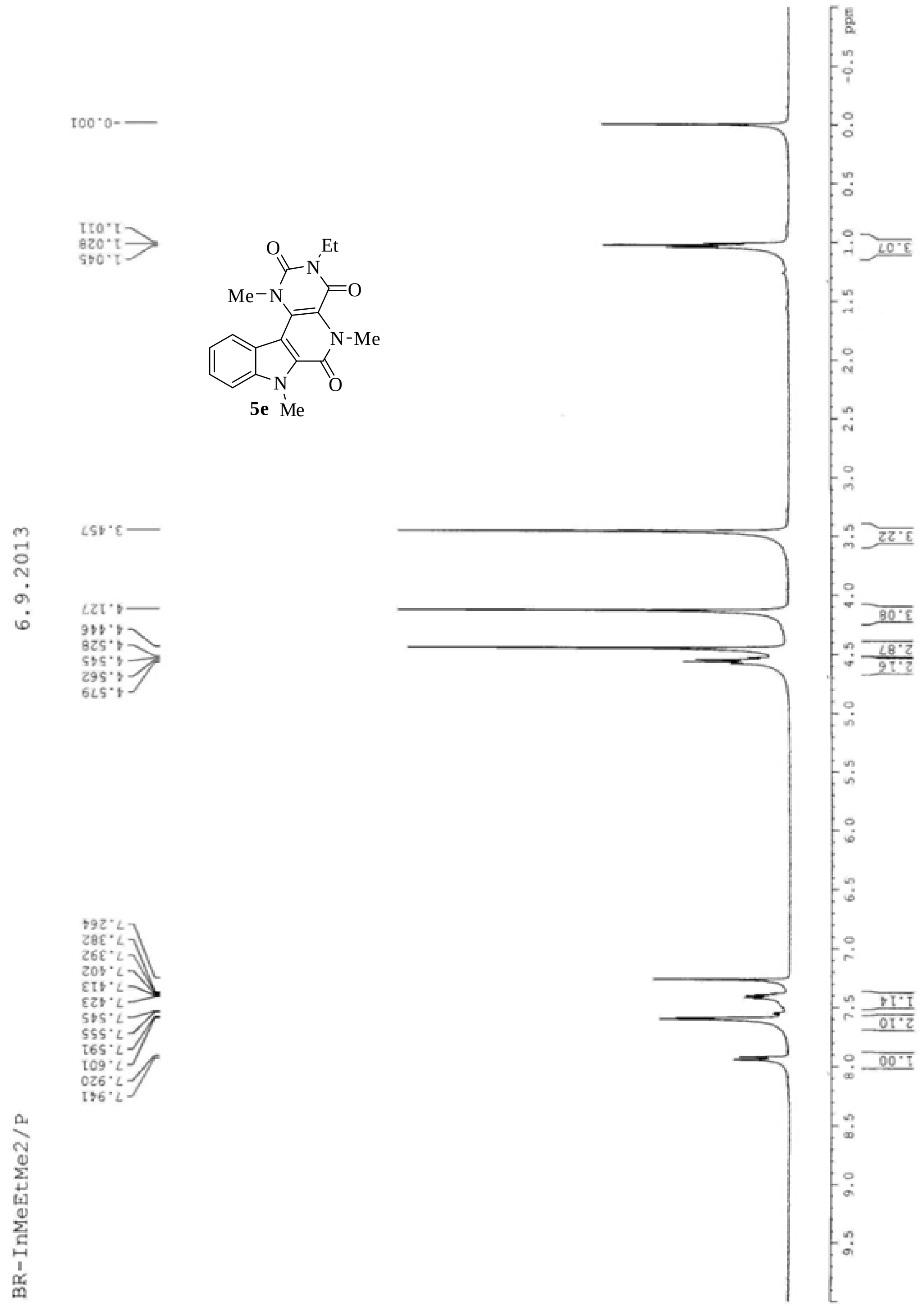

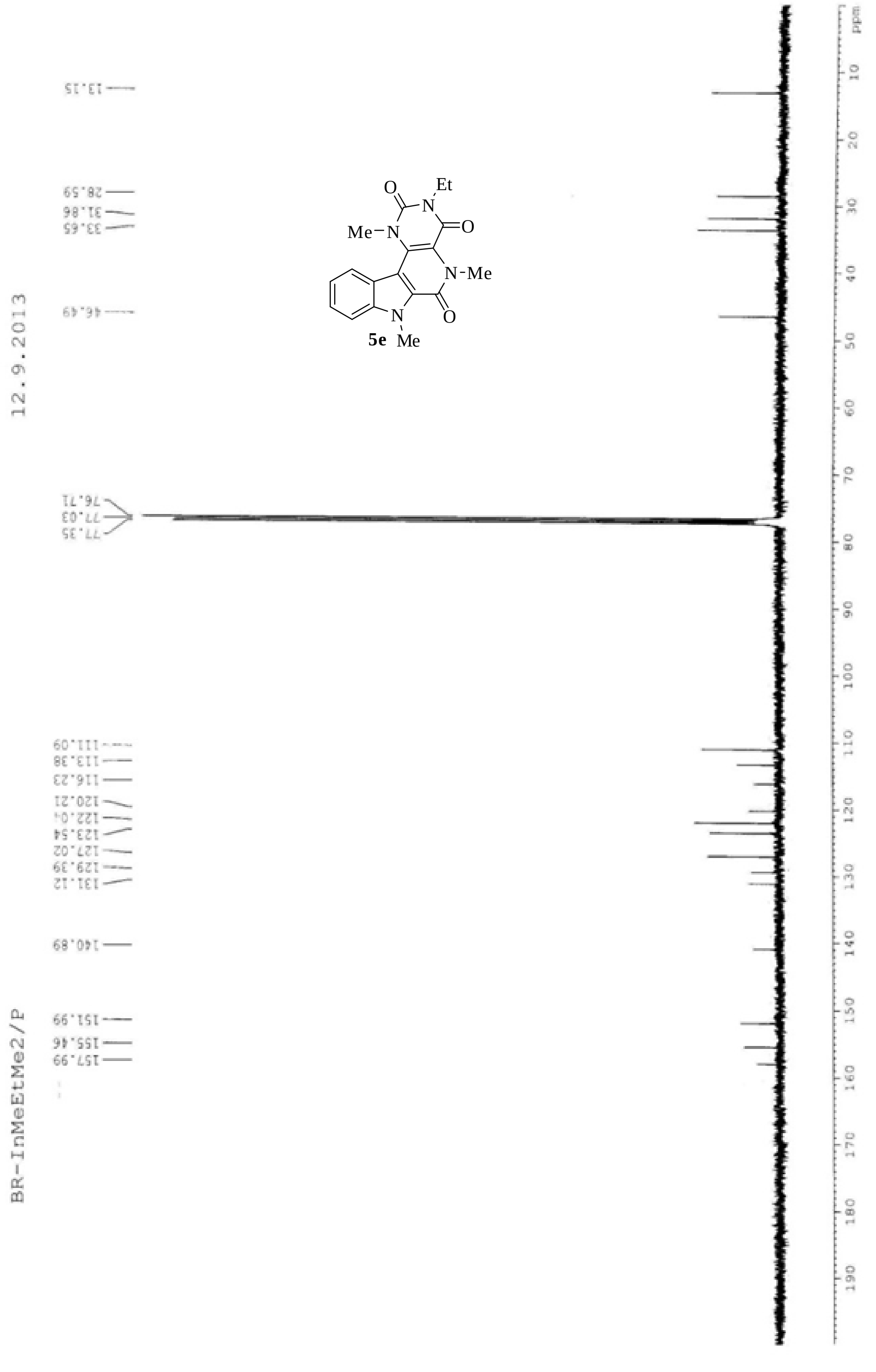
BR-InMeEtMe2/P
12.9.2013
13.15
28.59
31.86
33.65
46.49
76.71
77.03
77.35
111.09
113.38
116.23
120.21
122.04
123.54
127.02
129.39
131.12
140.89
151.99
155.46
157.99
Et
O
N
Me−N
O
N-Me
N
O
5e
Me
ppm
10
20
30
40
50
60
70
80
90
100
110
120
130
140
150
160
170
180
190

7-Ethyl-1,3,5-trimethyl-5,7-dihydro-1*H*-pyrimido[4',5':5,6]pyrido[3,4-*b*]indole-2,4,6(3*H*)-trione (5f):

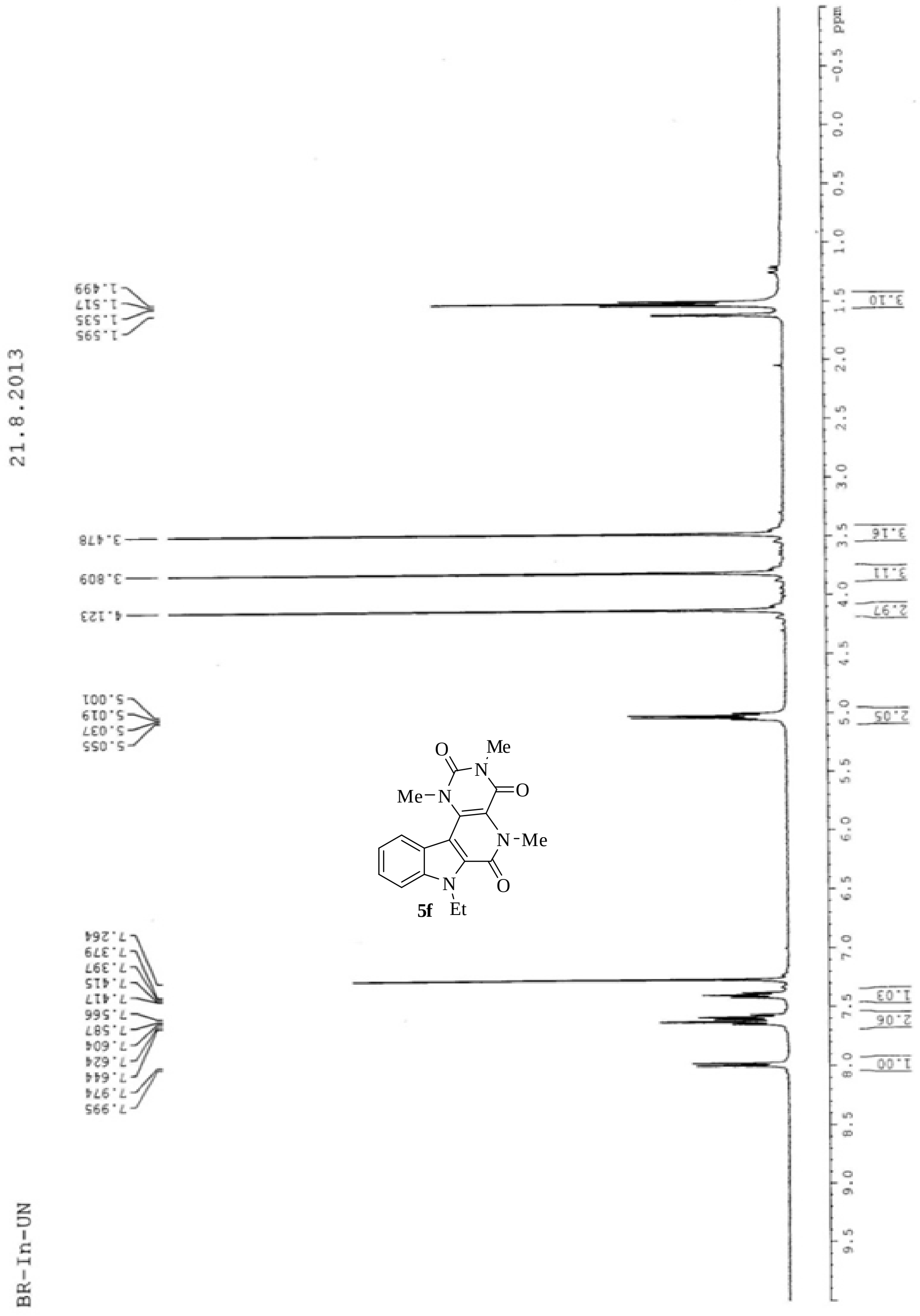

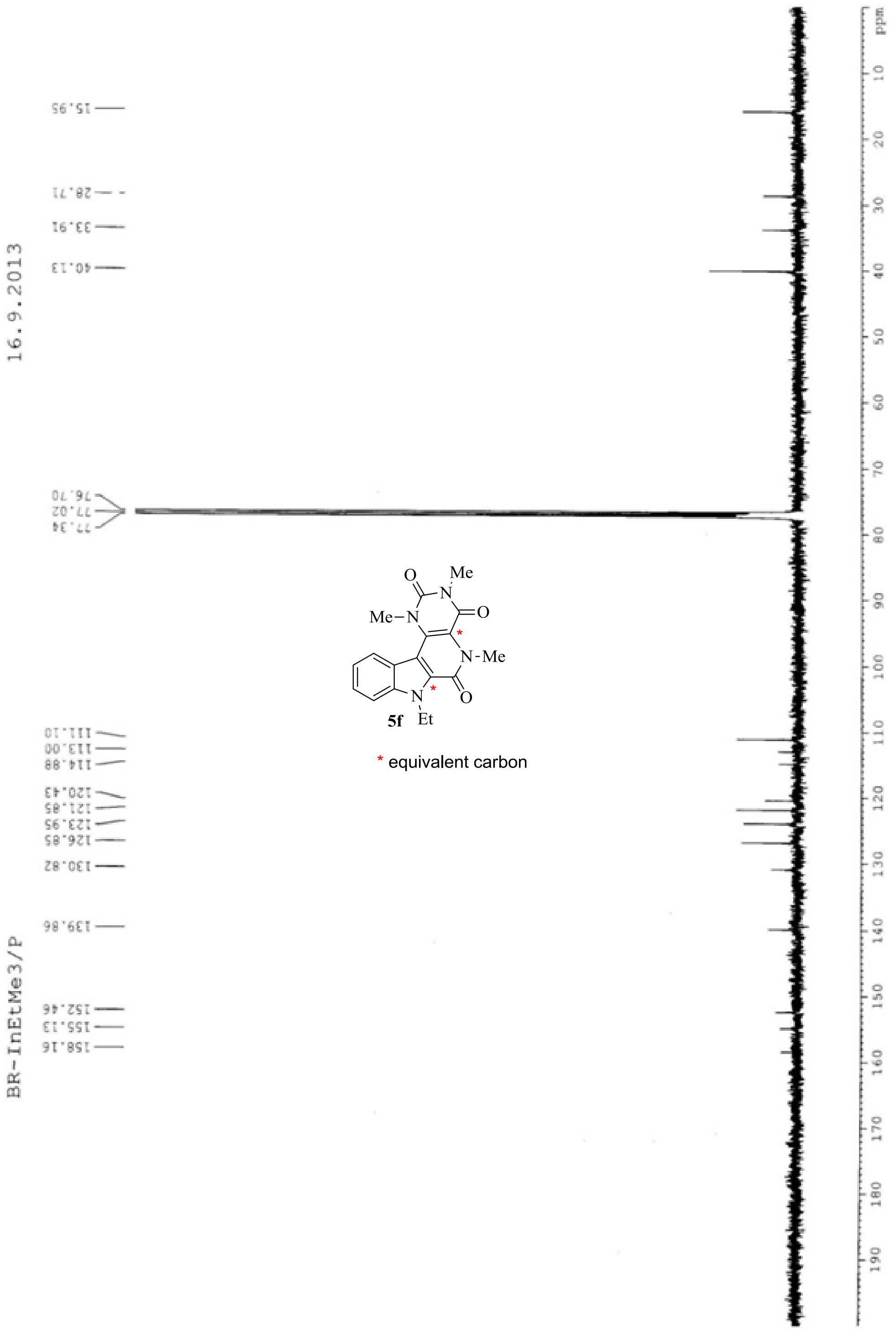
BR-InEtMe3/P
16.9.2013
15.95
28.71
33.91
40.13
76.70
77.02
77.34
111.10
113.00
114.88
120.43
121.85
123.95
126.85
130.82
139.86
152.46
155.13
158.16
ppm
Me
O
N
Me
N
O
N-Me
N
O
Et
5f
* equivalent carbon

5,7-Diethyl-1,3-dimethyl-5,7-dihydro-1*H*-pyrimido[4',5':5,6]pyrido[3,4-*b*]indole-2,4,6(3*H*)-trione (5g):

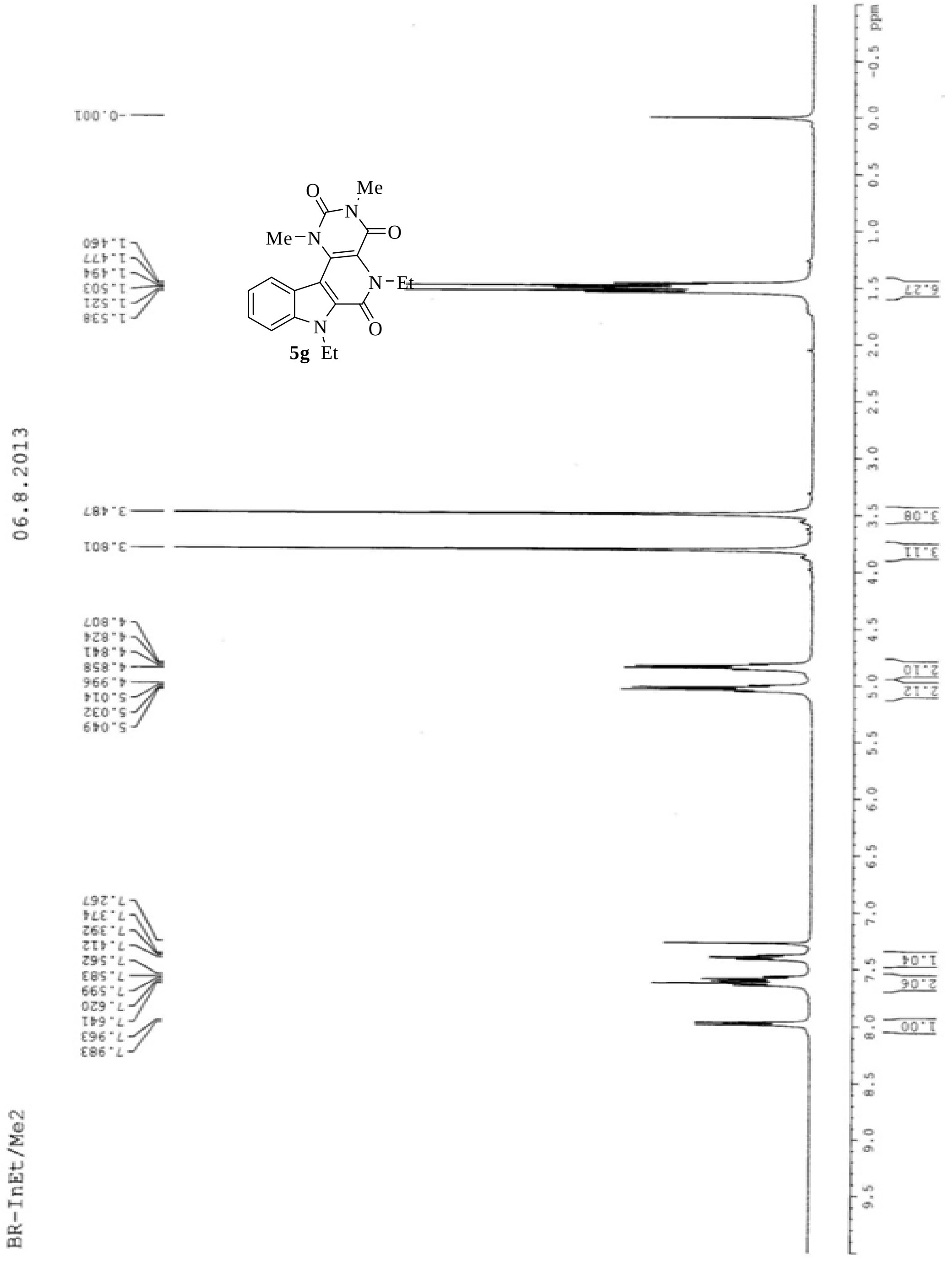

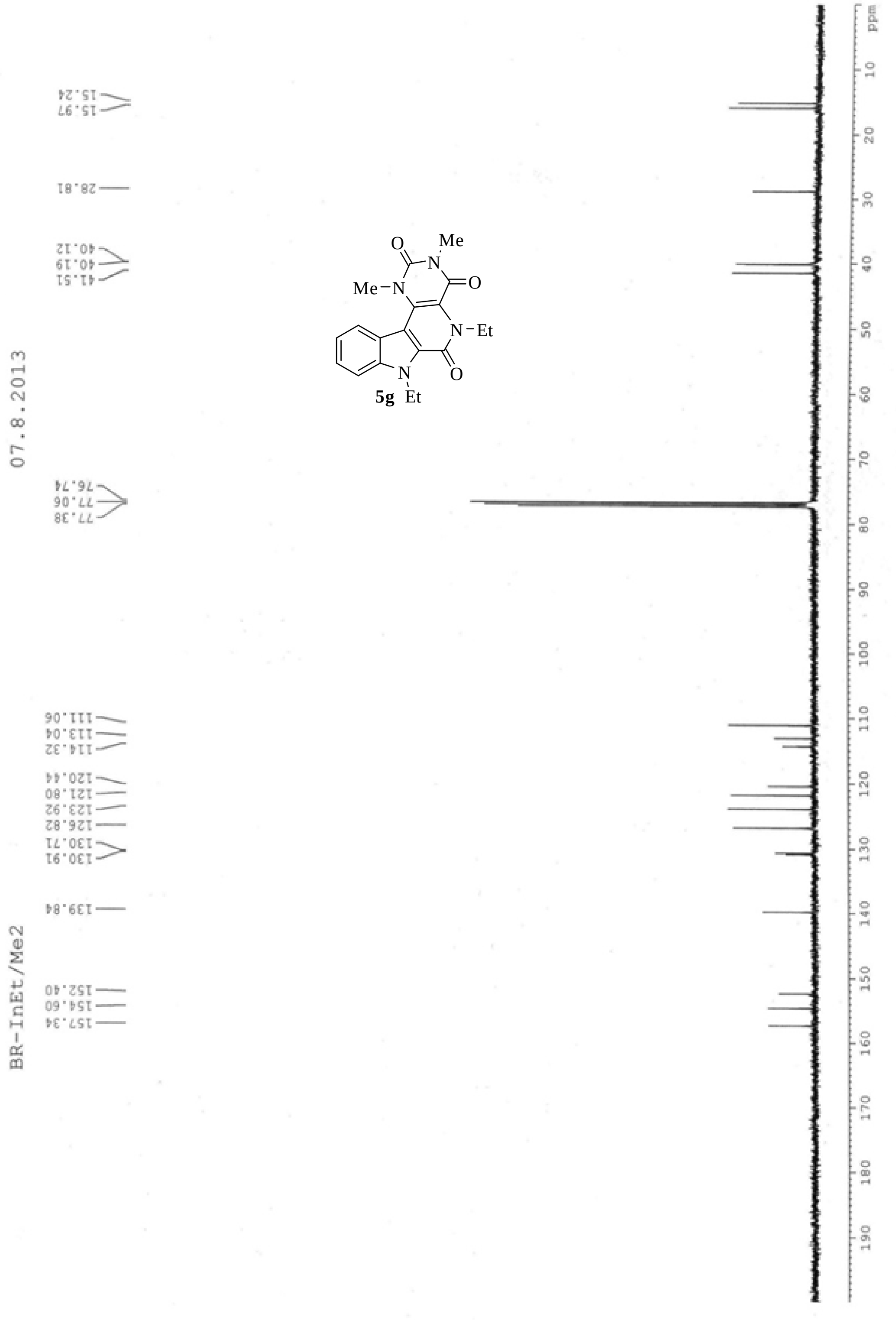
BR-InEt/Me2
07.8.2013
15.24
15.97
28.81
40.12
40.19
41.51
76.74
77.06
77.38
111.06
113.04
114.32
120.44
121.80
123.92
126.82
130.71
130.91
139.84
152.40
154.60
157.34
ppm
5g

1,3,7-Triethyl-5-methyl-5,7-dihydro-1*H*-pyrimido[4',5':5,6]pyrido[3,4-*b*]indole-2,4,6(3*H*)-trione (5h):

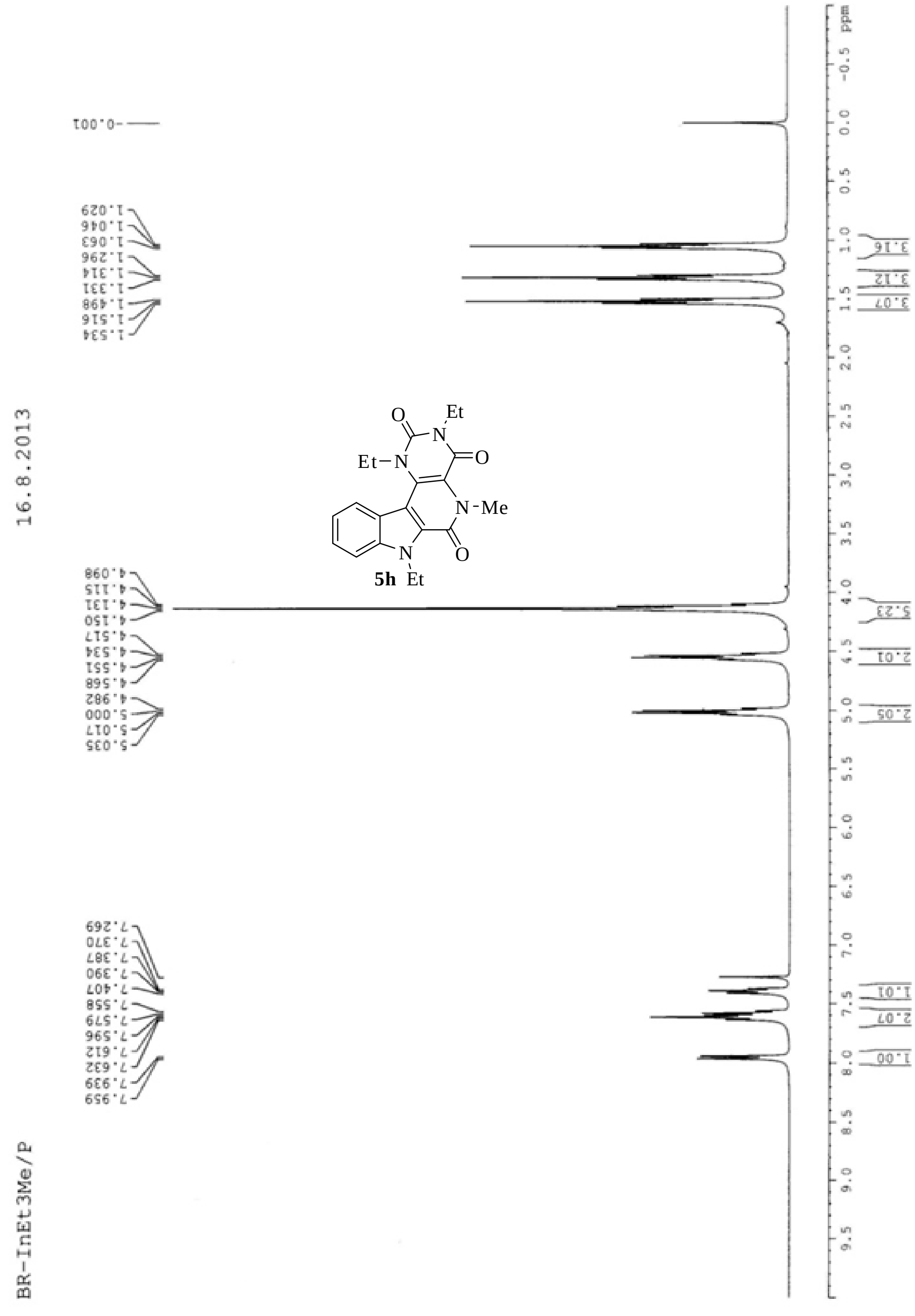

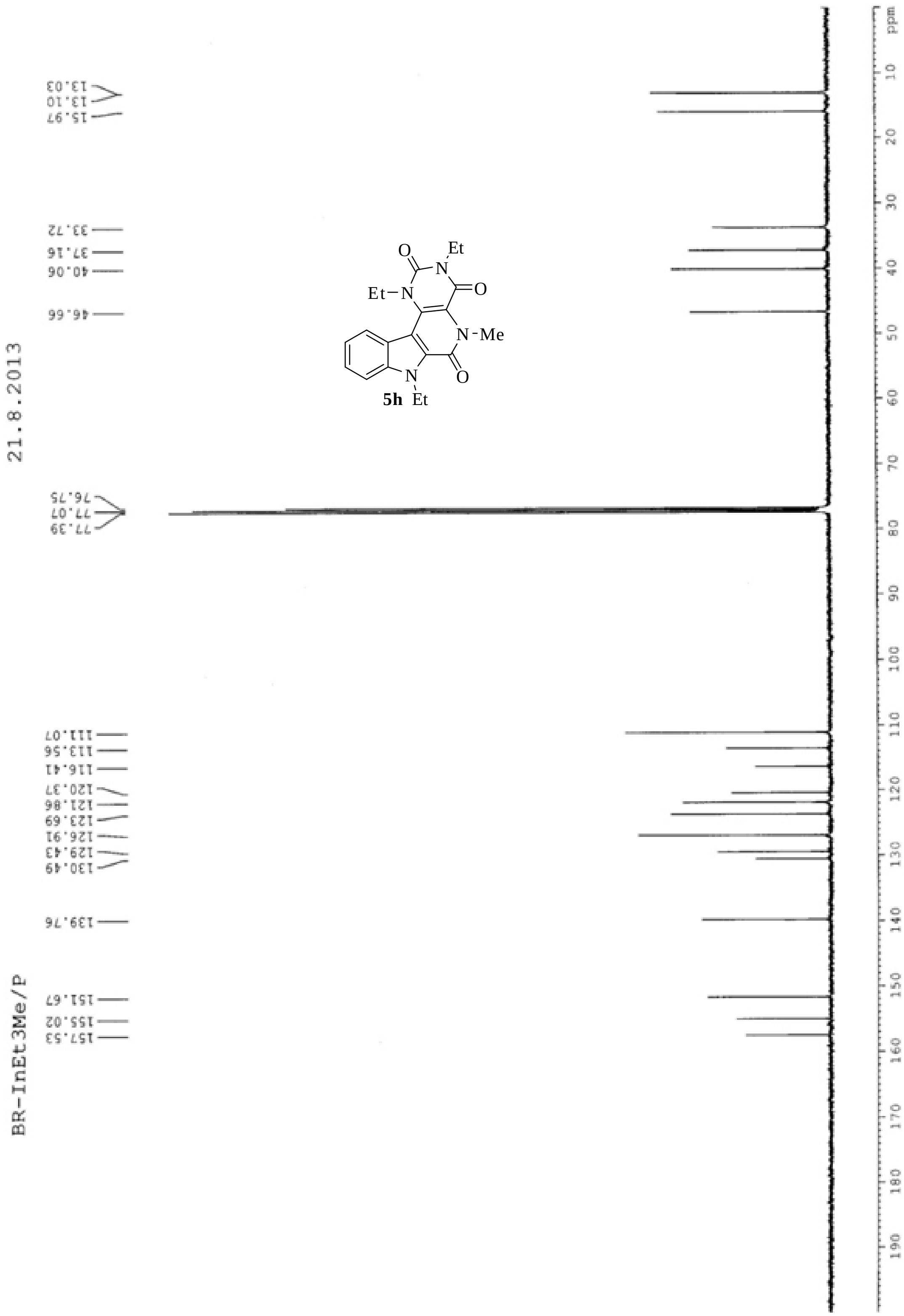

BR-InEt3Me/P
21.8.2013
13.03
13.10
15.97
33.72
37.16
40.06
46.66
76.75
77.07
77.39
111.07
113.56
116.41
120.37
121.86
123.69
126.91
129.43
130.49
139.76
151.67
155.02
157.53
ppm
O
Et
N
Et
N
O
N-Me
N
O
5h
Et

7-Benzyl-1,3,5-triethyl-5,7-dihydro-1*H*-pyrimido[4',5':5,6]pyrido[3,4-b]indole-2,4,6(3*H*)-trione (5i):

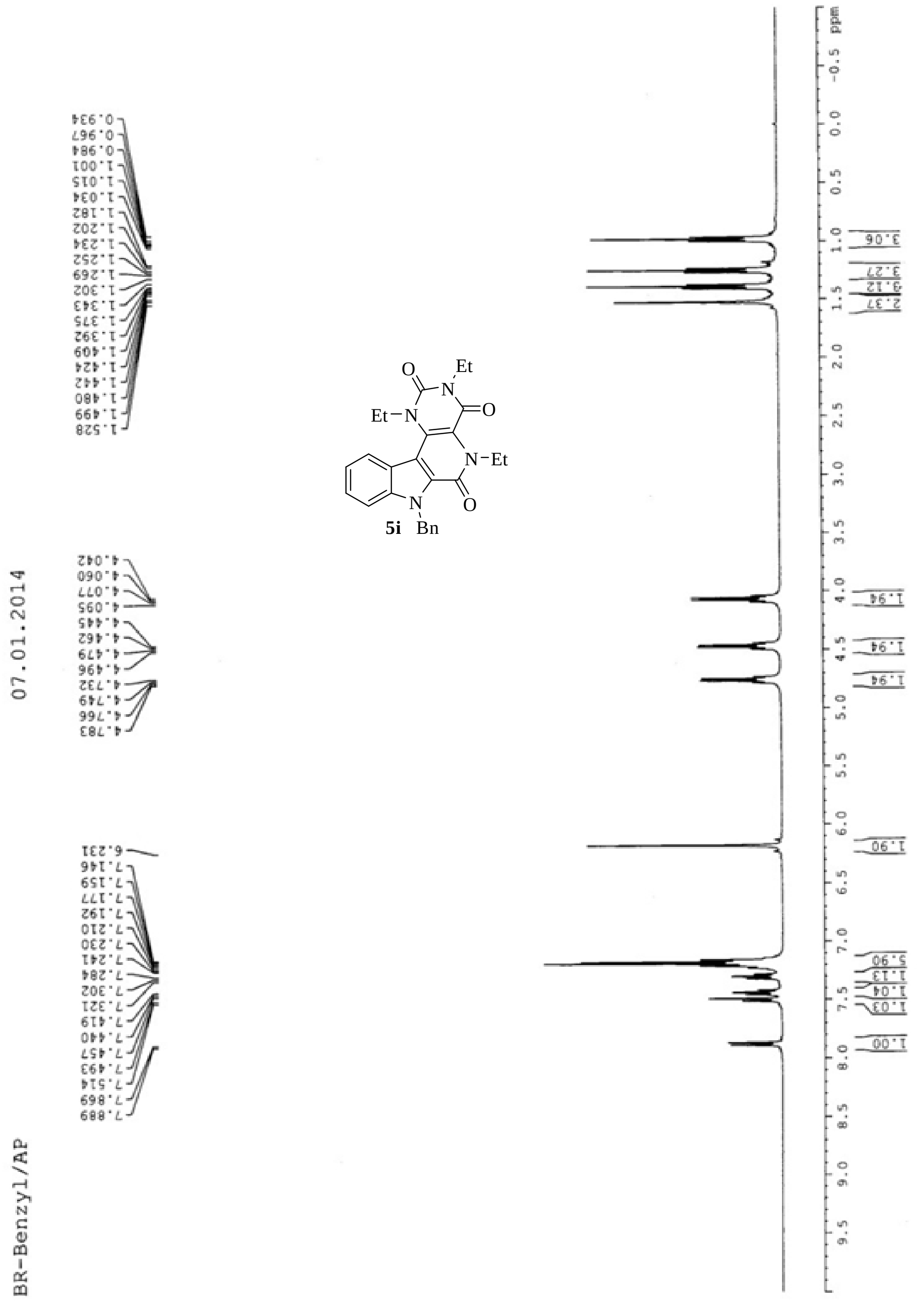

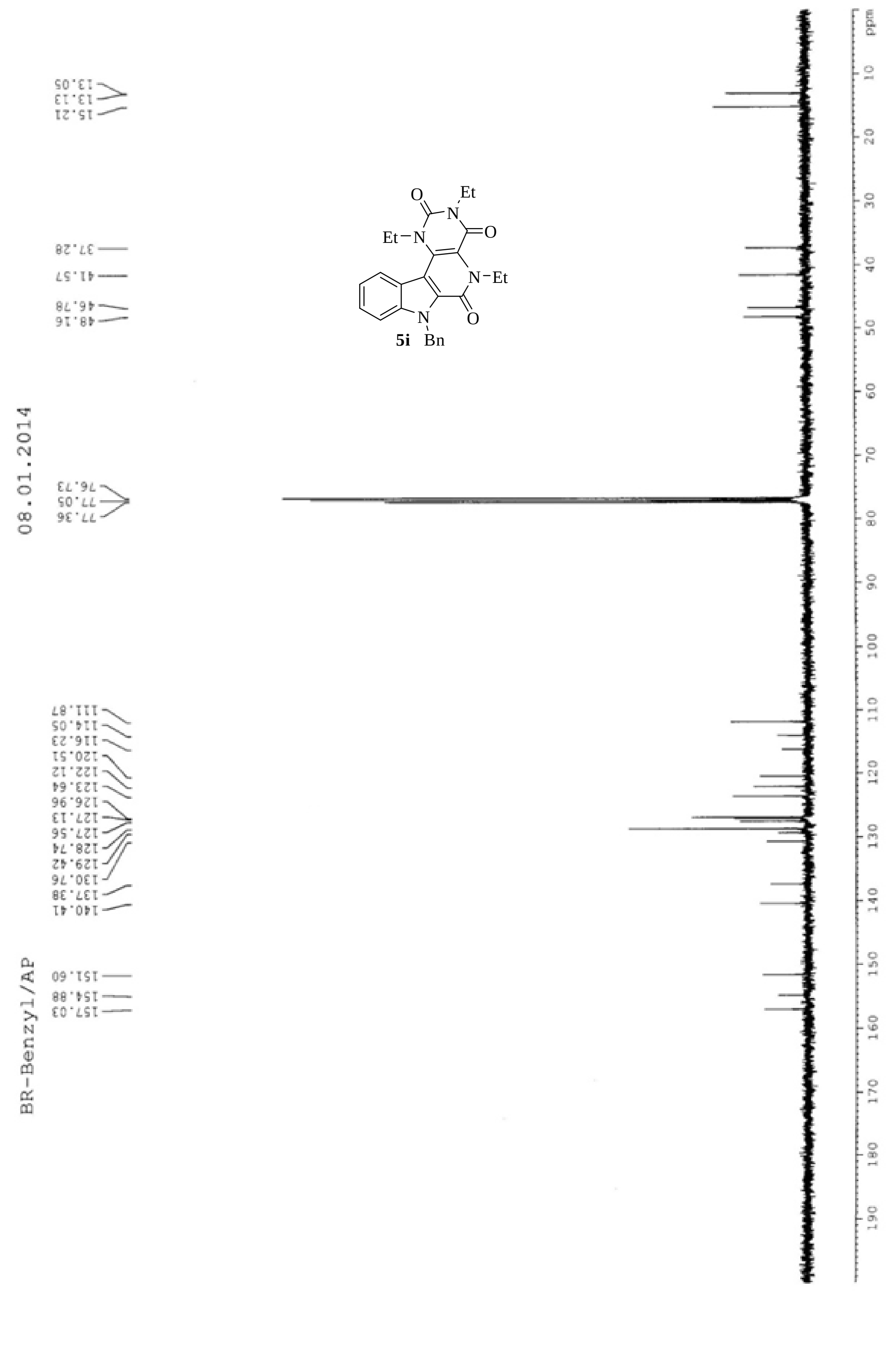
BR-Benzyl/AP
08.01.2014
13.05
13.13
15.21
37.28
41.57
46.78
48.16
76.73
77.05
77.36
111.87
114.05
116.23
120.51
122.12
123.64
126.96
127.13
127.56
128.74
129.42
130.76
137.38
140.41
151.60
154.88
157.03
O
Et
N
Et
O
N-Et
O
Bn
5i
ppm

7-Benzyl-1,3-diethyl-5-methyl-5,7-dihydro-1*H*-pyrimido[4',5':5,6]pyrido[3,4-*b*]indole-2,4,6(3*H*)-trione (5j):

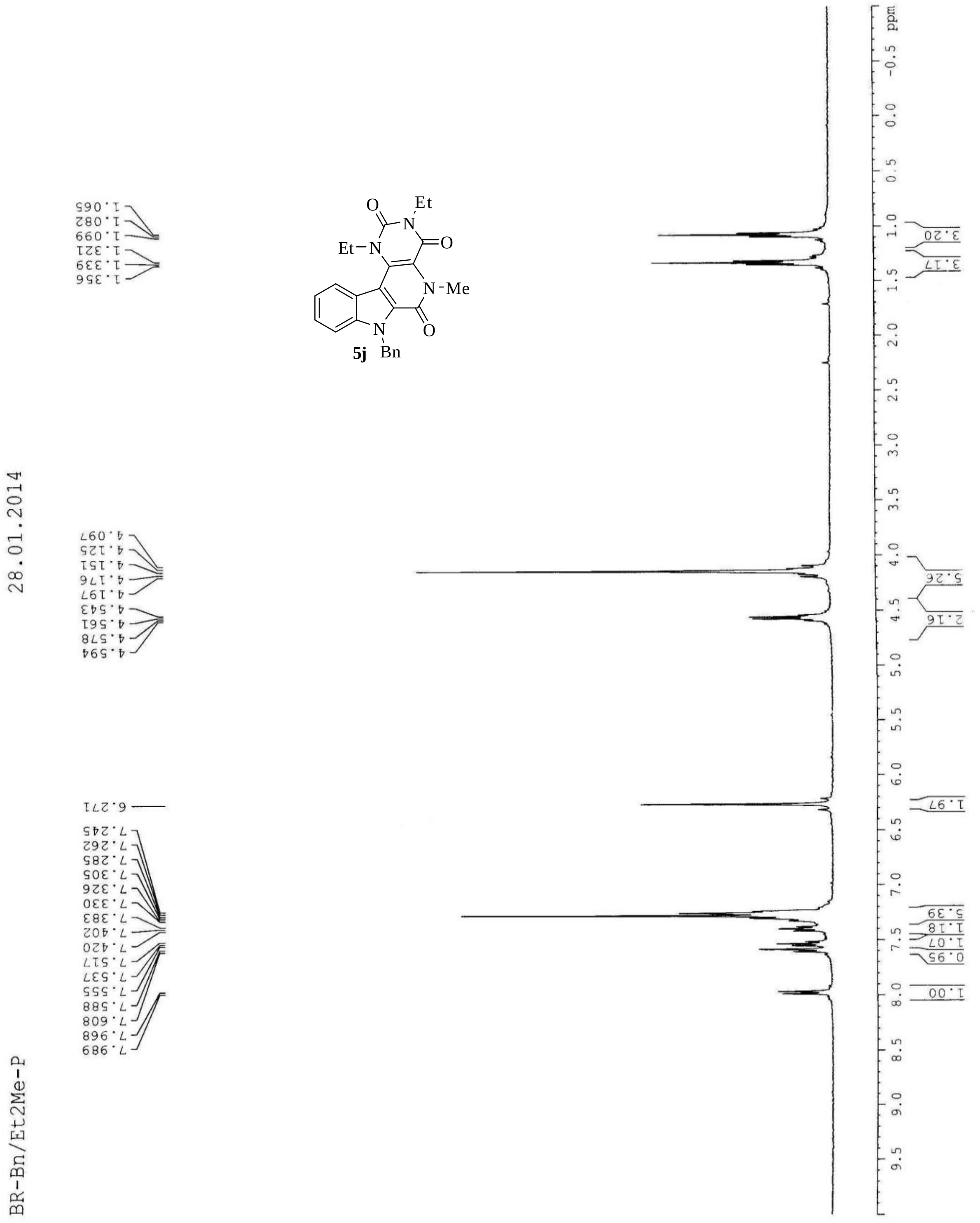

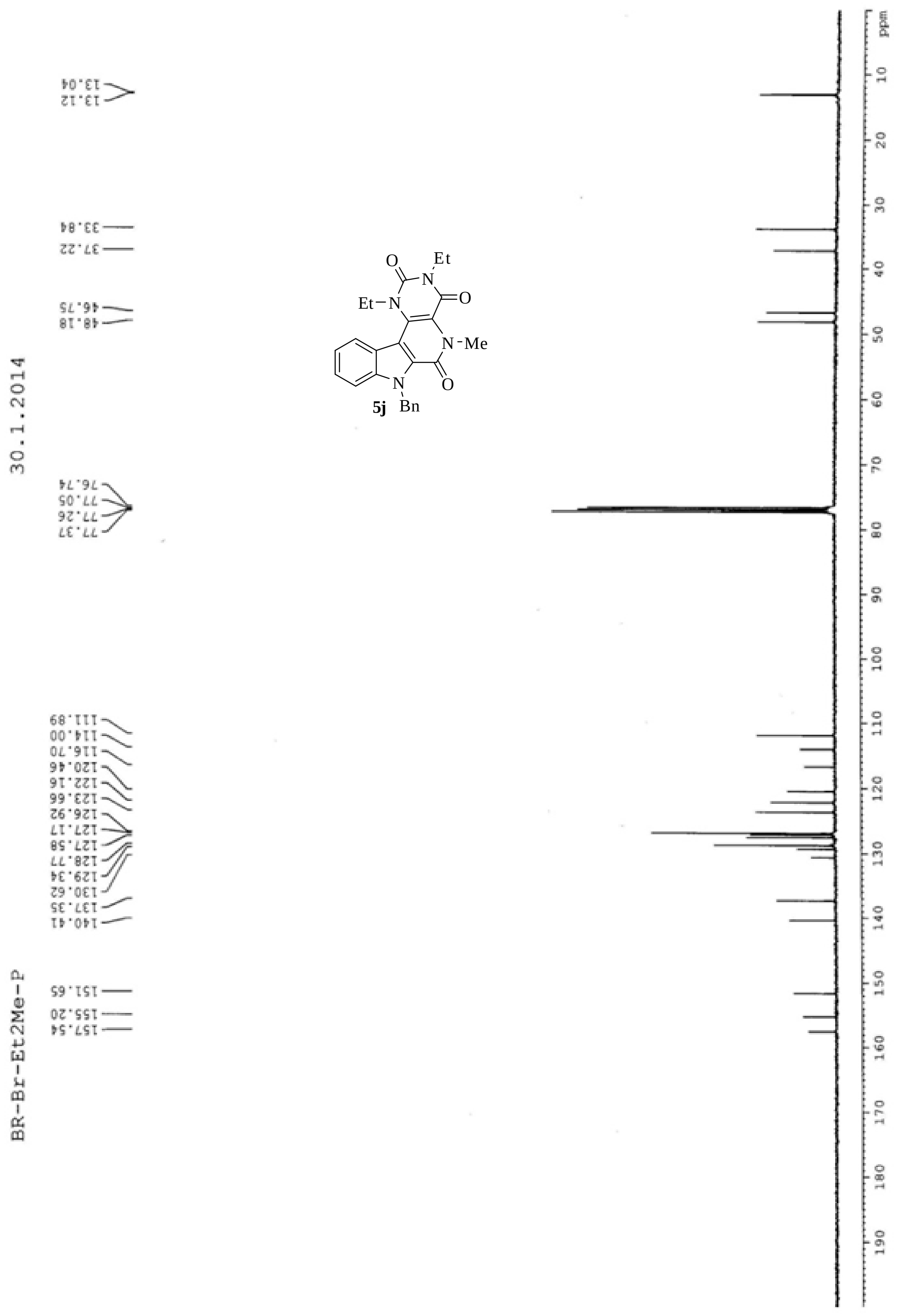
BR-Br-Et2Me-P
30.1.2014
13.04
13.12
33.84
37.22
46.75
48.18
76.74
77.05
77.26
77.37
111.89
114.00
116.70
120.46
122.16
123.66
126.92
127.17
127.58
128.77
129.34
130.62
137.35
140.41
151.65
155.20
157.54
ppm
5j
Et
Et
N
O
Me
Bn

7-Butyl-1,3-diethyl-5-methyl-5,7-dihydro-1*H*-pyrimido[4',5':5,6]pyrido[3,4-*b*]indole-2,4,6(3*H*)-trione (5k):

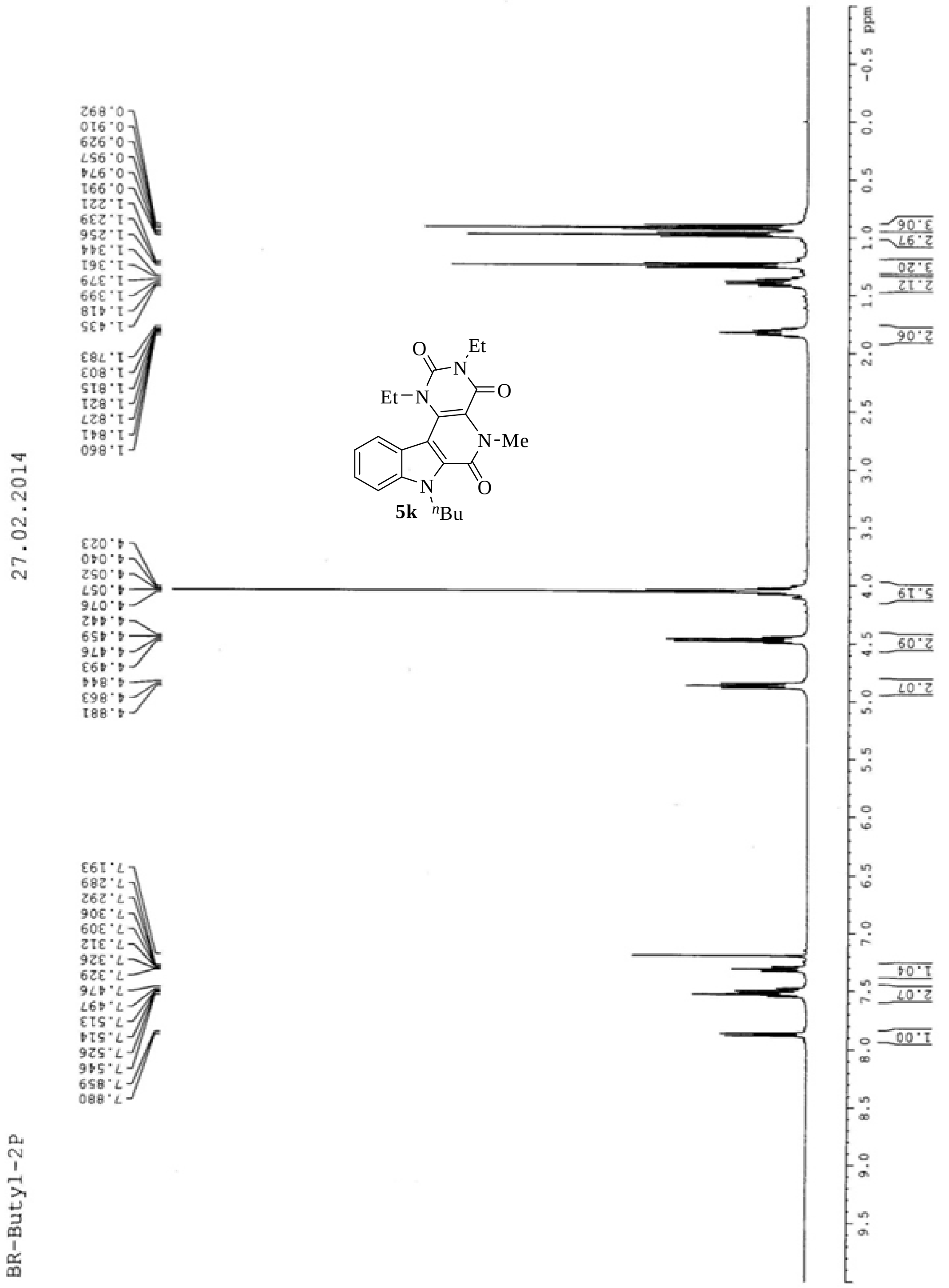

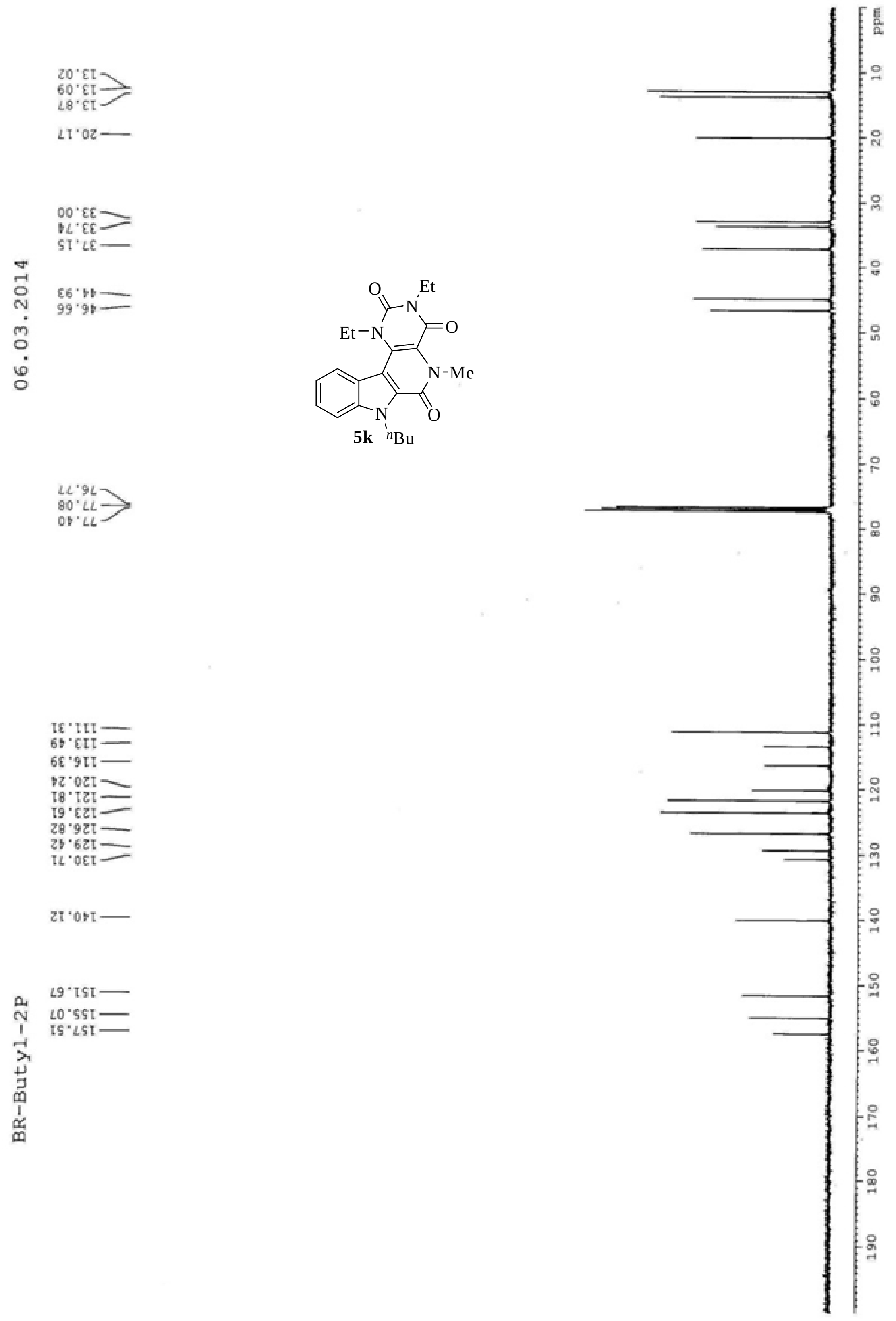
BR-Butyl-2P
06.03.2014
157.51
155.07
151.67
140.12
130.71
129.42
126.82
123.61
121.81
120.24
116.39
113.49
111.31
77.40
77.08
76.77
46.66
44.93
37.15
33.74
33.00
20.17
13.87
13.09
13.02
ppm
5k

7-Butyl-1,3,5-triethyl-5,7-dihydro-1*H*-pyrimido[4',5':5,6]pyrido[3,4-*b*]indole-2,4,6(3*H*)-trione (5l):

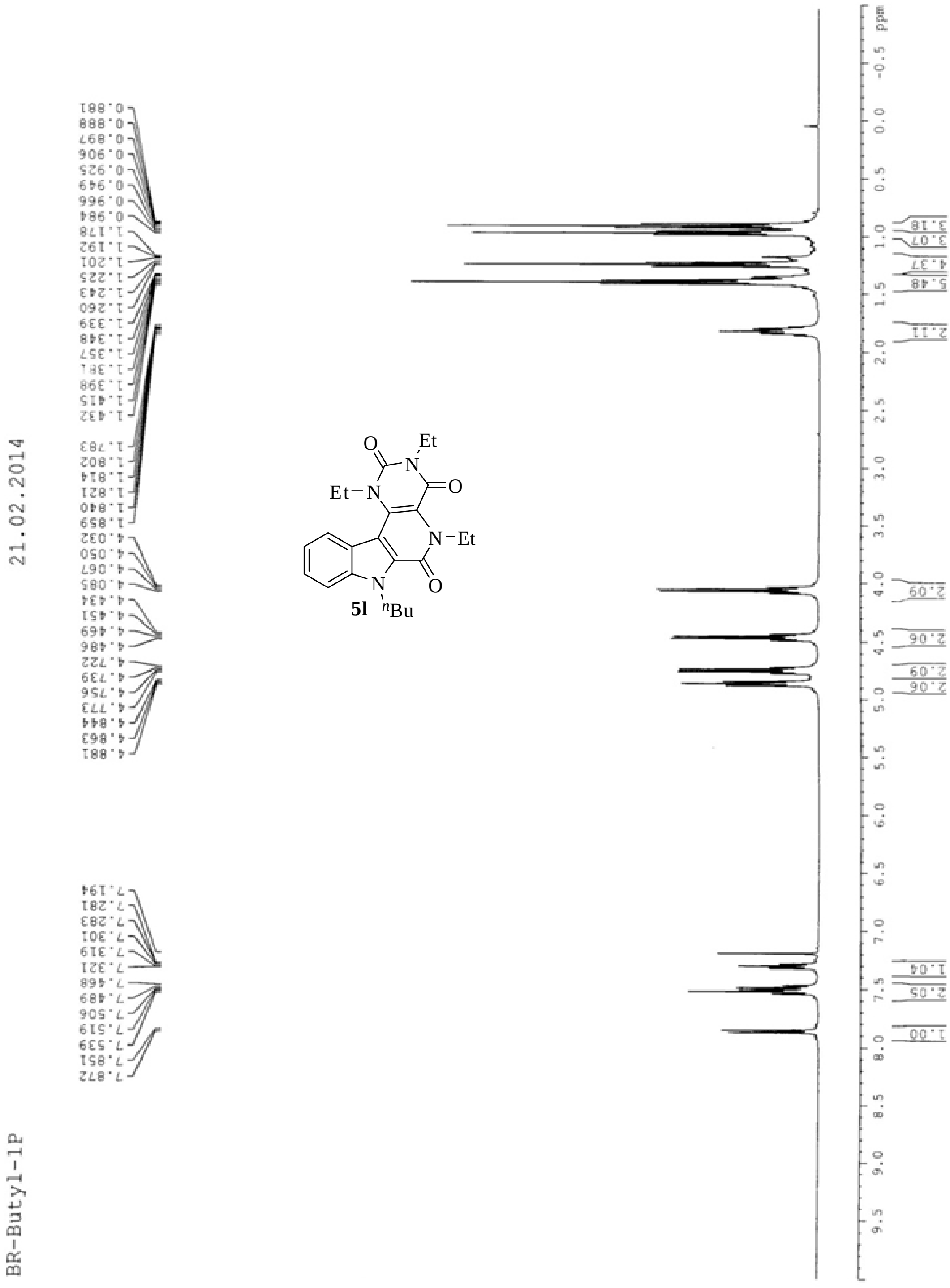

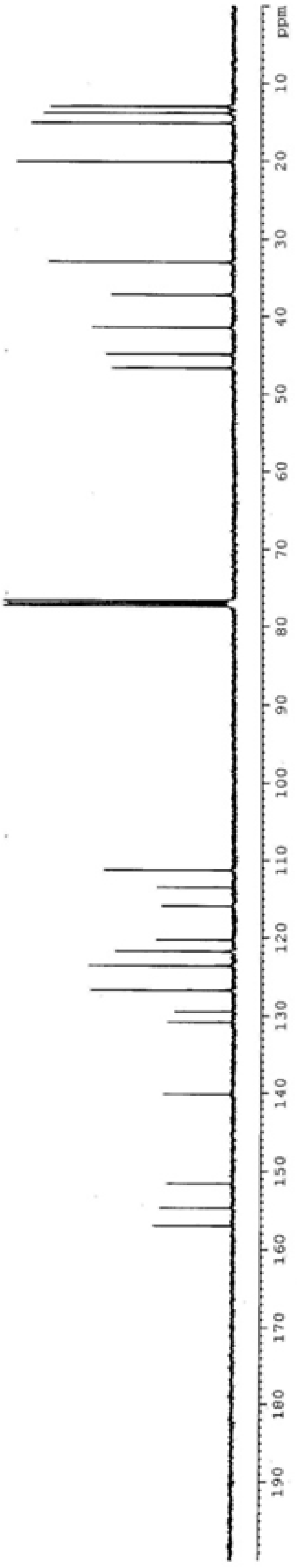

BR-Butyl-1P
26.02.2014
13.03
13.09
13.91
15.20
20.16
33.02
37.21
41.46
44.92
46.70
76.75
77.07
77.39
111.29
113.54
115.93
120.28
121.77
123.60
126.78
129.50
130.86
140.13
151.62
154.74
157.00
ppm

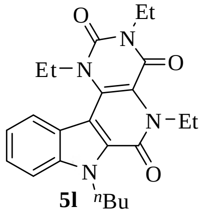

O
Et
N
Et
N
O
N
Et
N
O
nBu

5l

NMR (^{1}H and ^{13}C) spectra of starting materials (4):

***N*-(1,3-Dimethyl-2,4-dioxo-1,2,3,4-tetrahydropyrimidin-5-yl)-*N*,1-dimethyl-1*H*-indole-2-carboxamide (4a):**

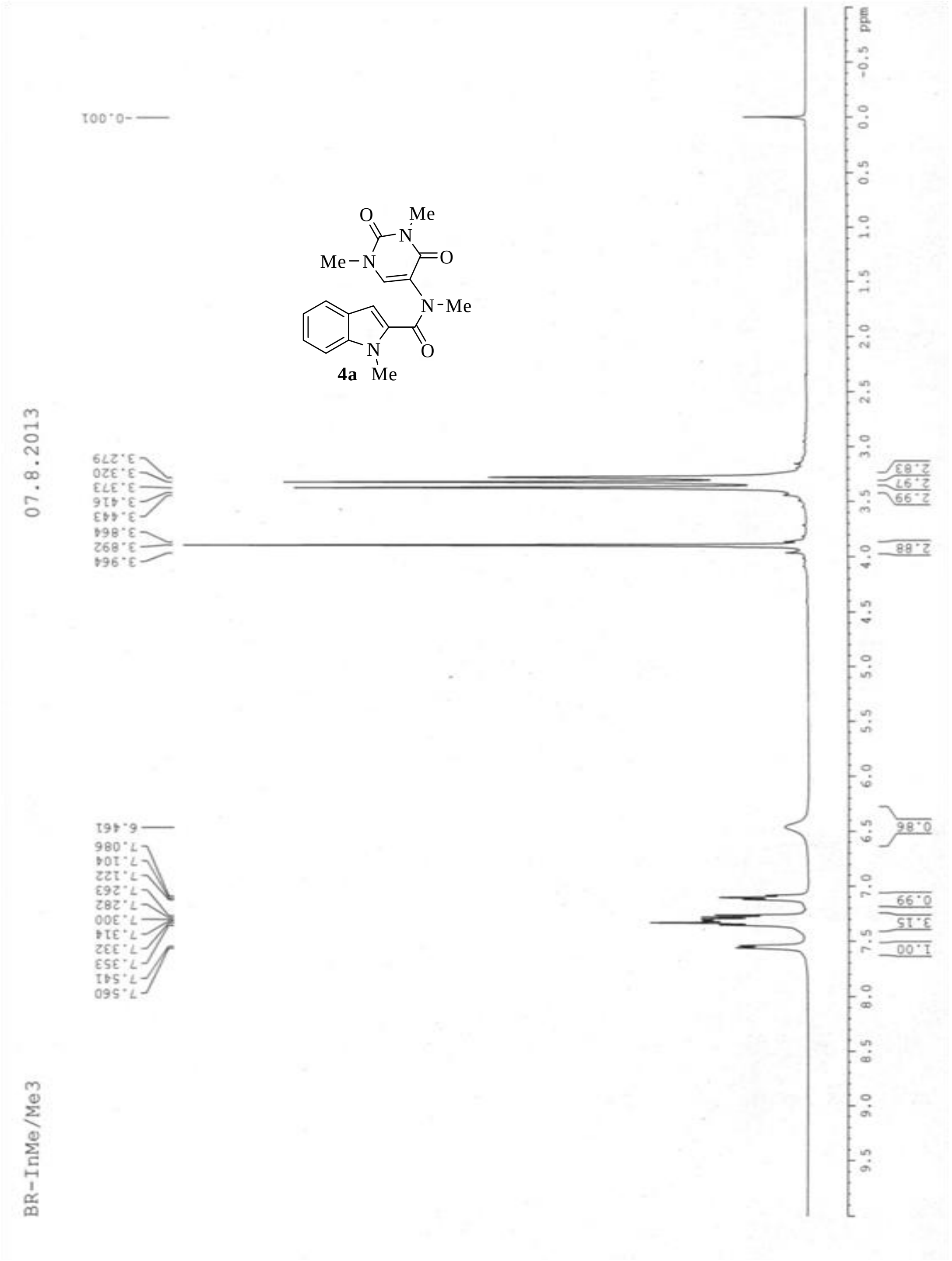

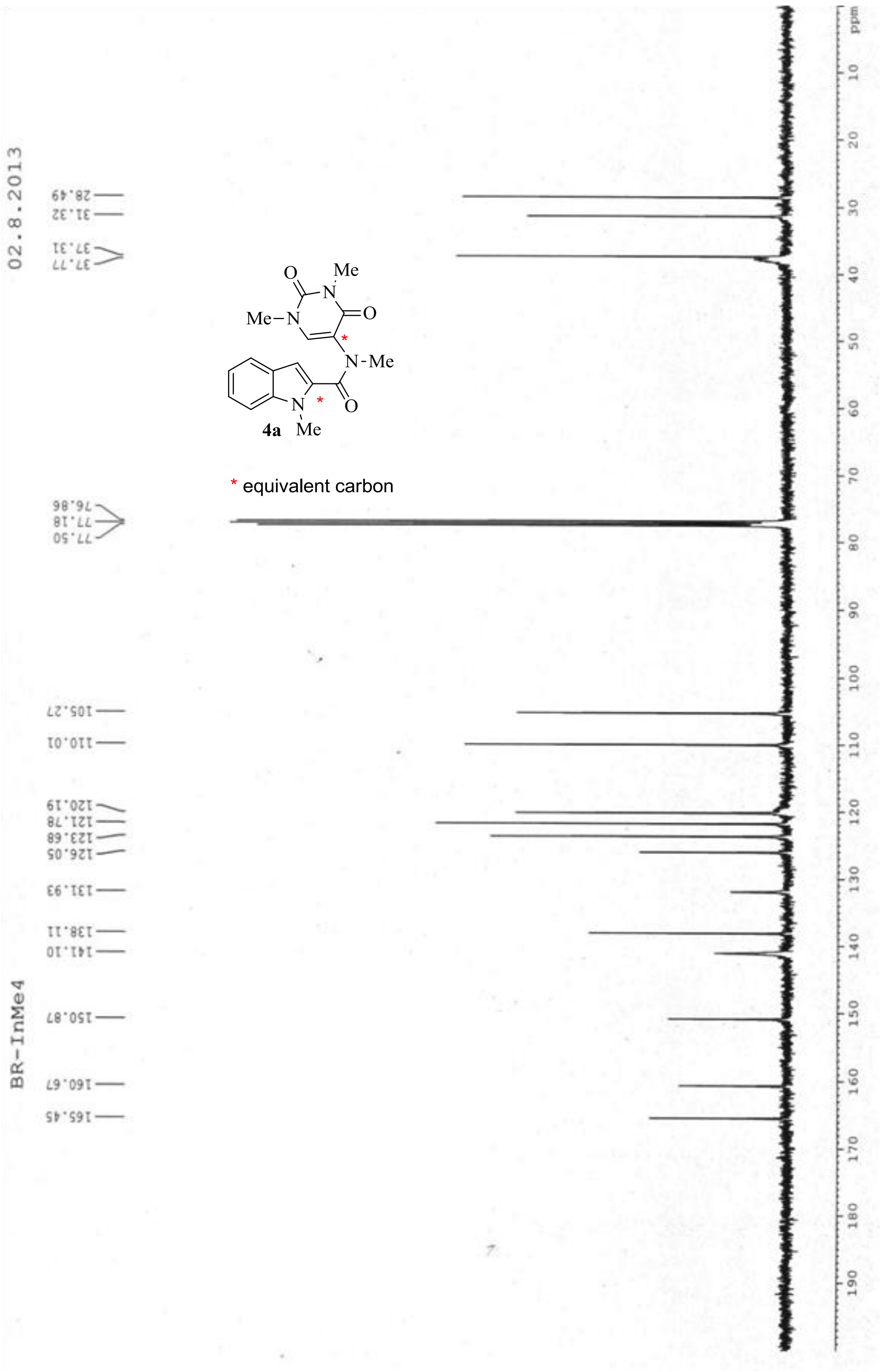
02.8.2013
BR-InMe4
28.49
31.32
37.31
37.77
76.86
77.18
77.50
105.27
110.01
120.19
121.78
123.68
126.05
131.93
138.11
141.10
150.87
160.67
165.45
Me
O
N
Me−N
O
N-Me
N
O
Me
4a
* equivalent carbon
ppm

N-(1,3-Diethyl-2,4-dioxo-1,2,3,4-tetrahydropyrimidin-5-yl)-N,1-dimethyl-1H-indole-2-carboxamide (4b):

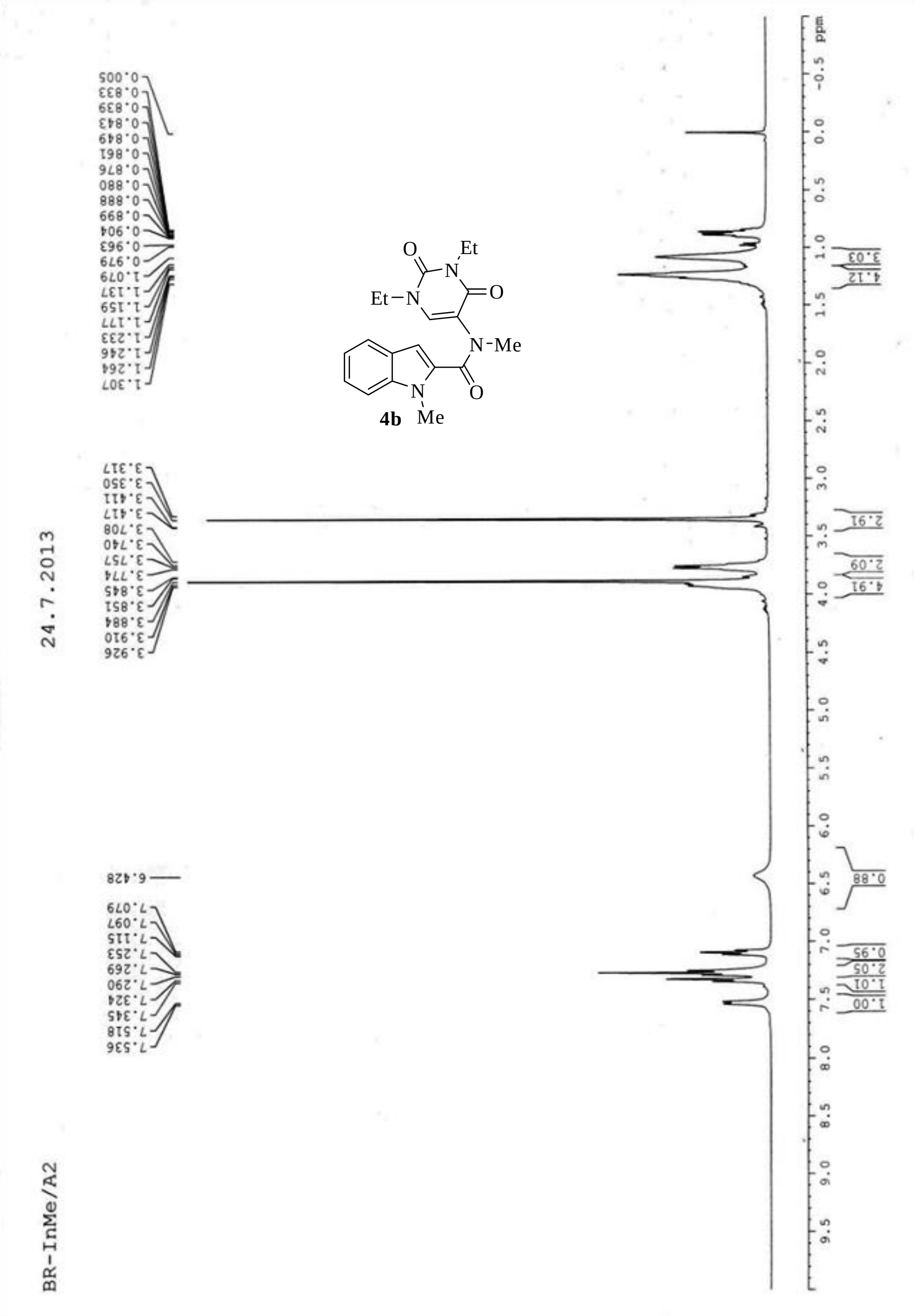

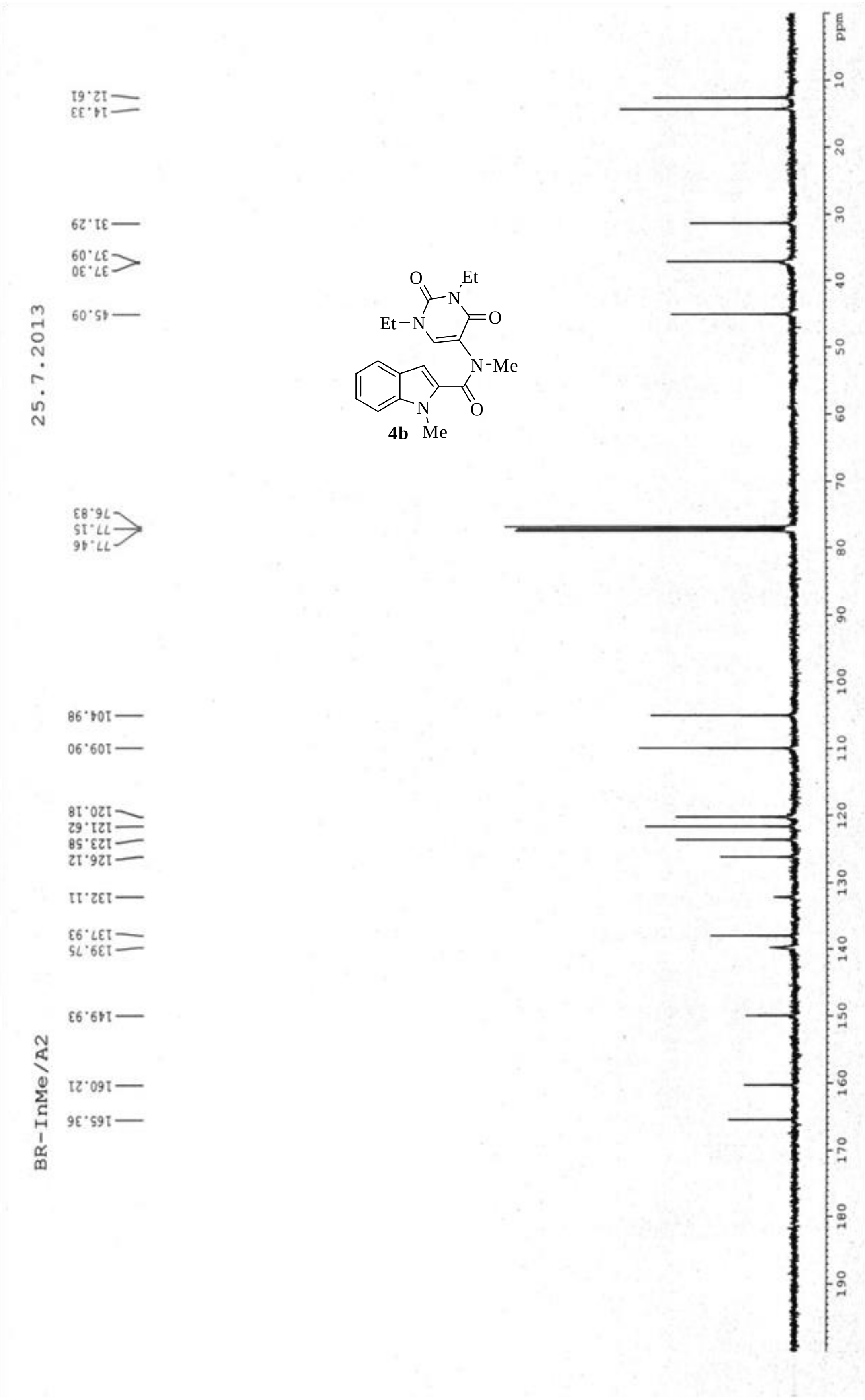
25.7.2013
BR-InMe/A2
12.61
14.33
31.29
37.09
37.30
45.09
76.83
77.15
77.46
104.98
109.90
120.18
121.62
123.58
126.12
132.11
137.93
139.75
149.93
160.21
165.36
ppm
4b

***N*-(1,3-Dimethyl-2,4-dioxo-1,2,3,4-tetrahydropyrimidin-5-yl)-*N*-ethyl-1-methyl-1*H*-indole-2-carboxamide (4c):**

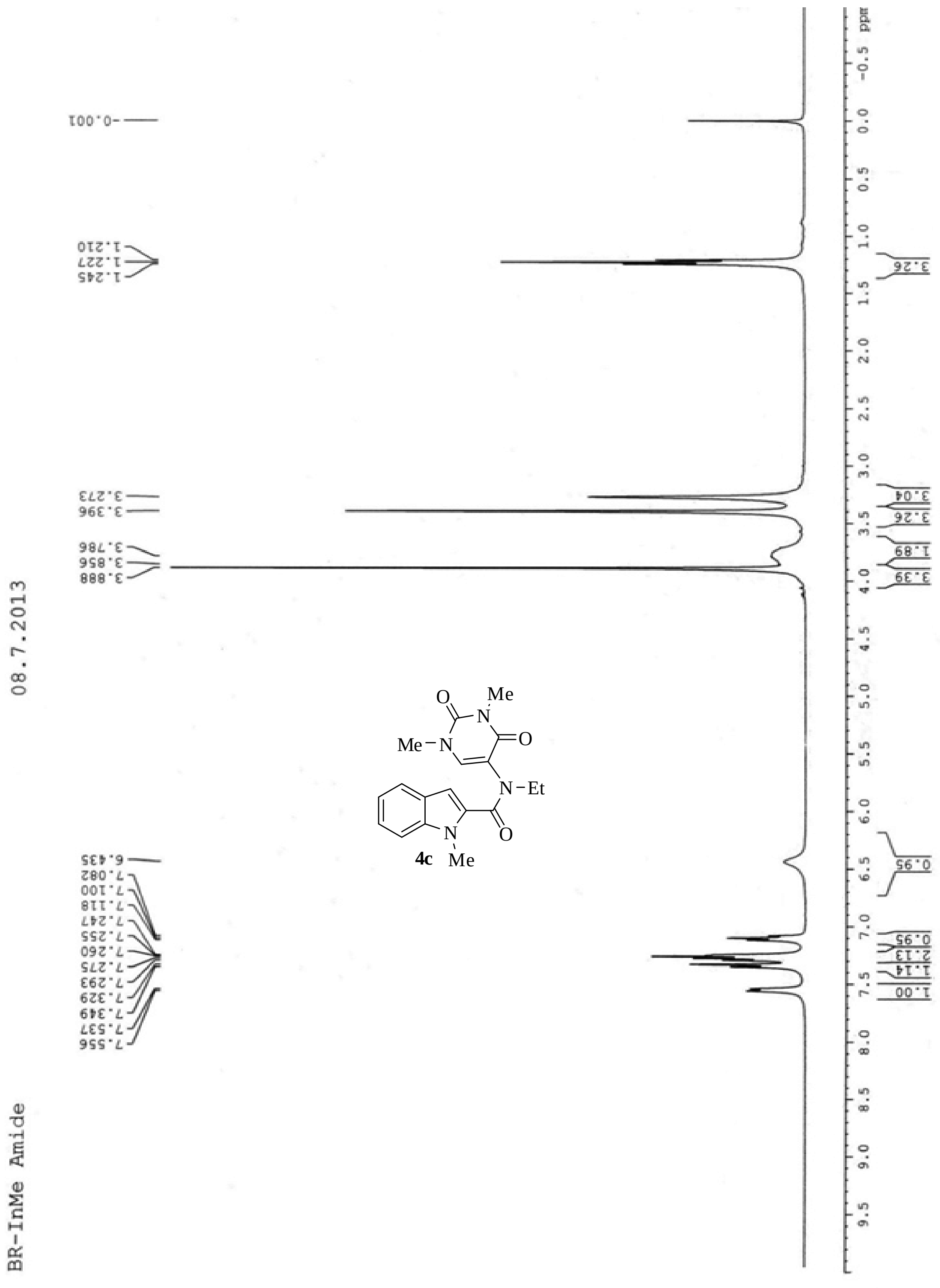

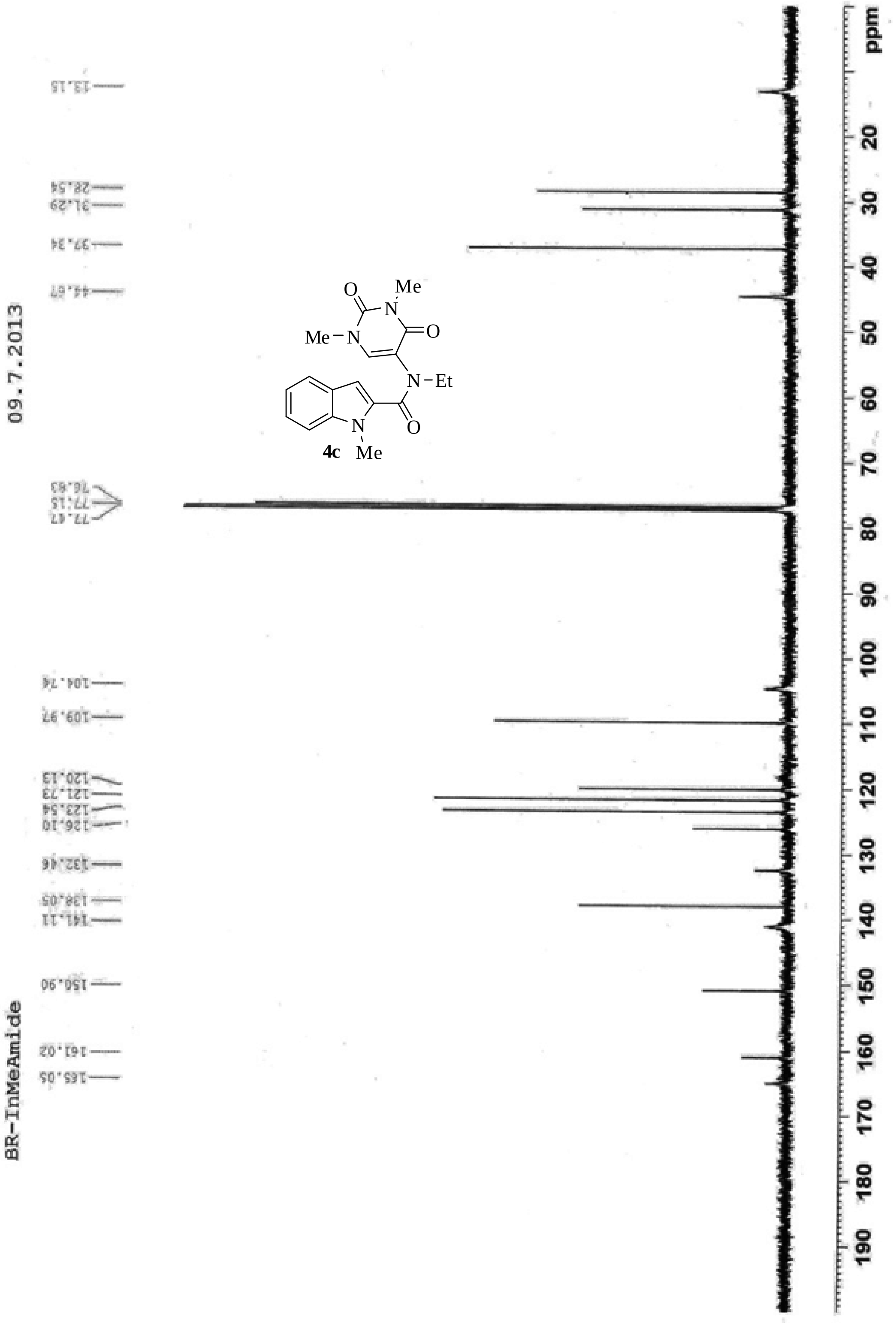

BR-InMeAmide
09.7.2013
165.05
161.02
150.90
141.11
138.05
132.46
126.10
123.54
121.73
120.13
109.97
104.74
77.47
77.15
76.83
44.67
37.34
31.29
28.54
13.15
ppm
O
Me
N
Me
N
O
N
Et
N
O
4c
Me

DEPT spectra of 4c:

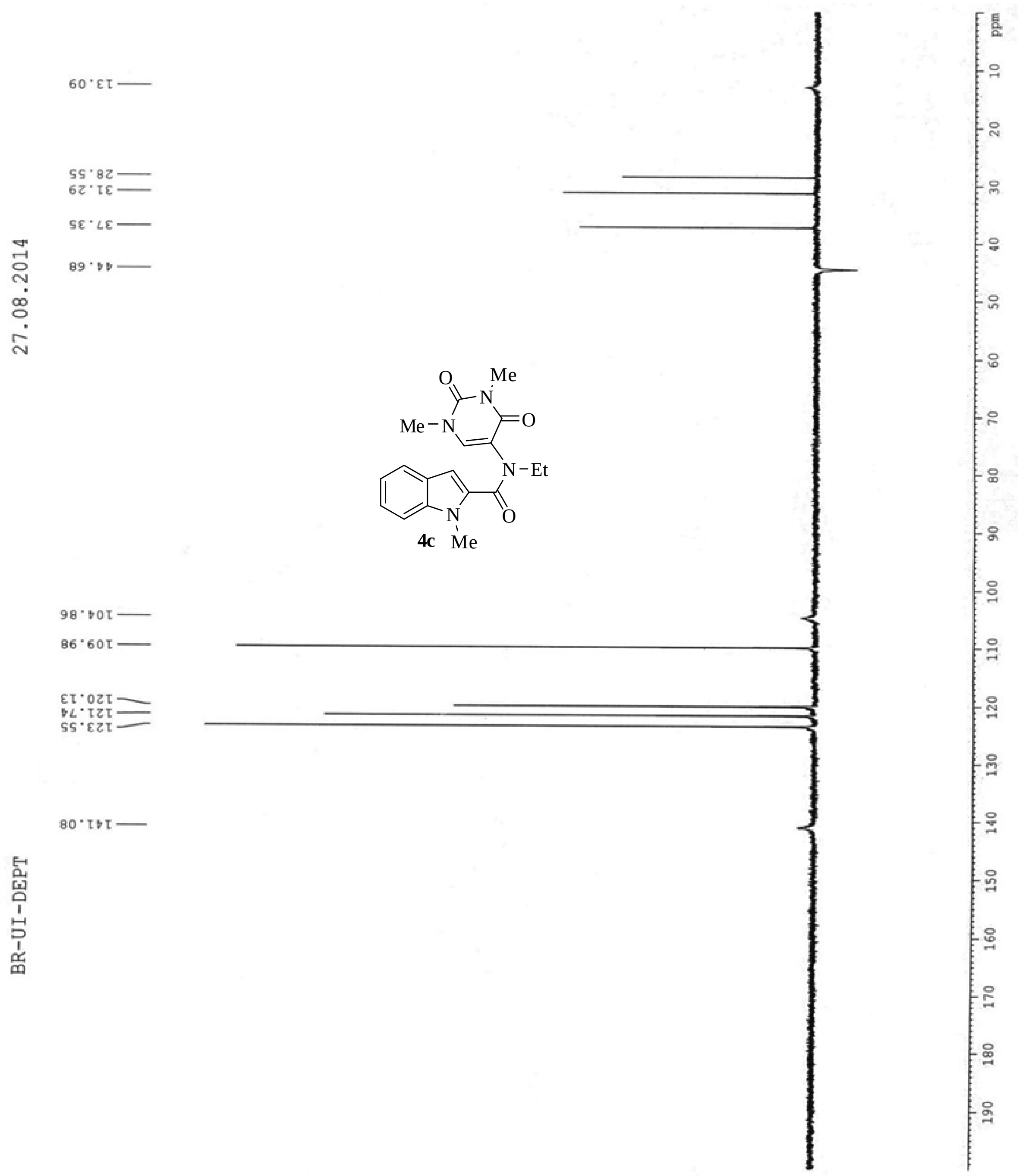

***N*-Ethyl-*N*-(3-ethyl-1-methyl-2,4-dioxo-1,2,3,4-tetrahydropyrimidin-5-yl)-1-methyl-1*H*-indole-2-carboxamide (4d):**

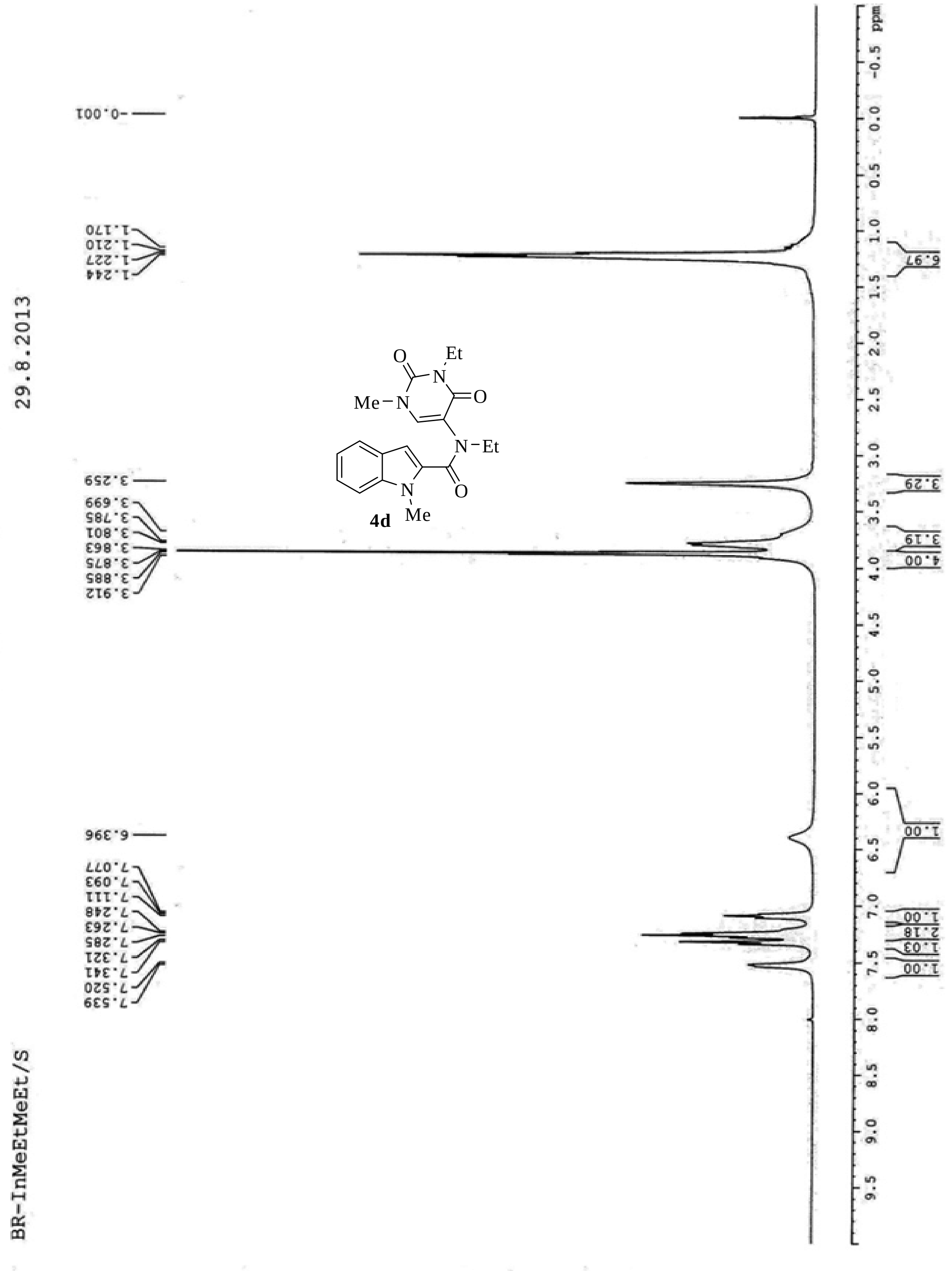

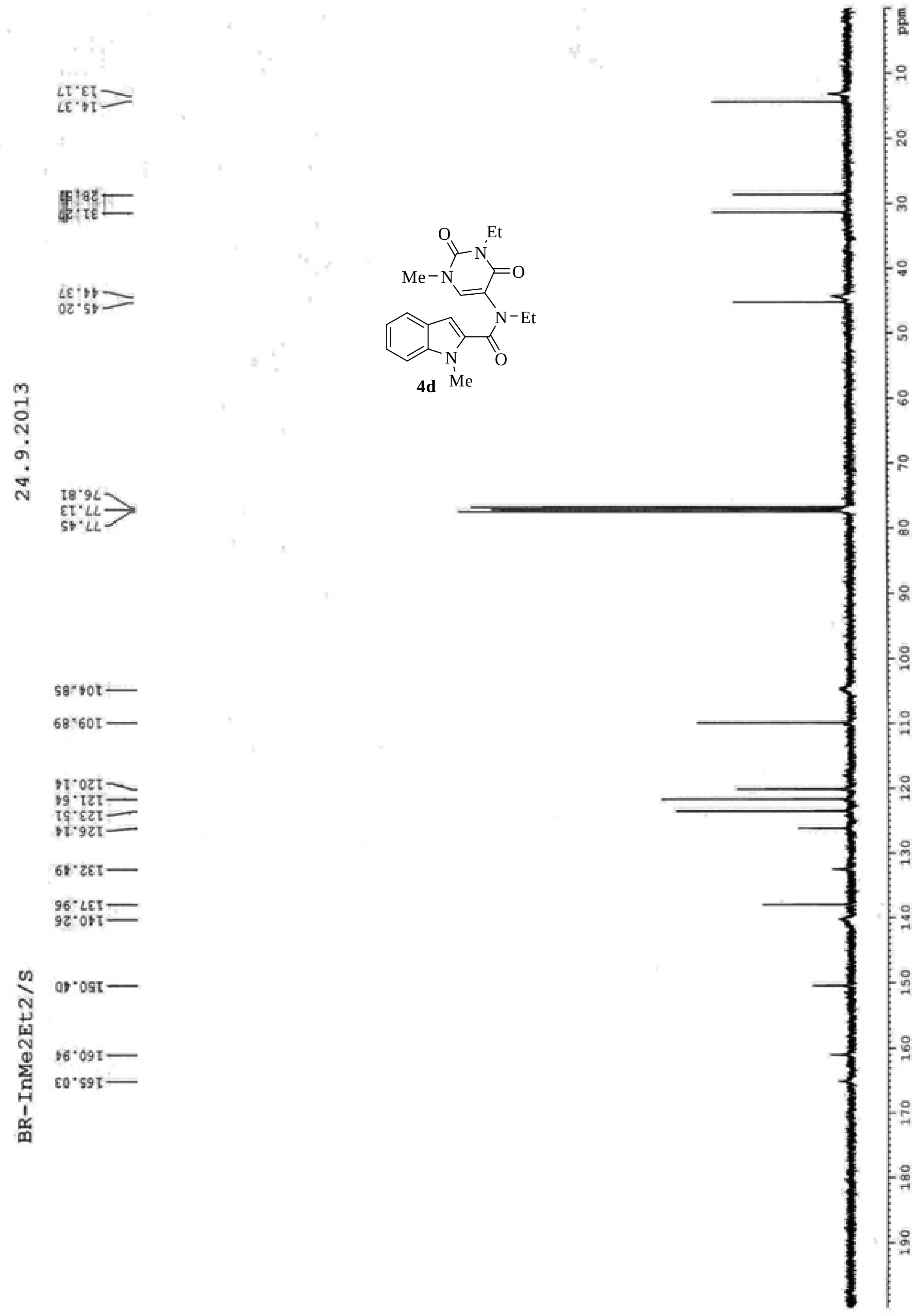
BR-InMe2Et2/S
24.9.2013
13.17
14.37
44.37
45.20
76.81
77.13
77.45
104.85
109.89
120.14
121.64
123.51
126.14
132.49
137.96
140.26
150.40
160.94
165.03
4d
ppm

N-(3-Ethyl-1-methyl-2,4-dioxo-1,2,3,4-tetrahydropyrimidin-5-yl)-N,1-dimethyl-1H-indole-2-carboxamide (4e):

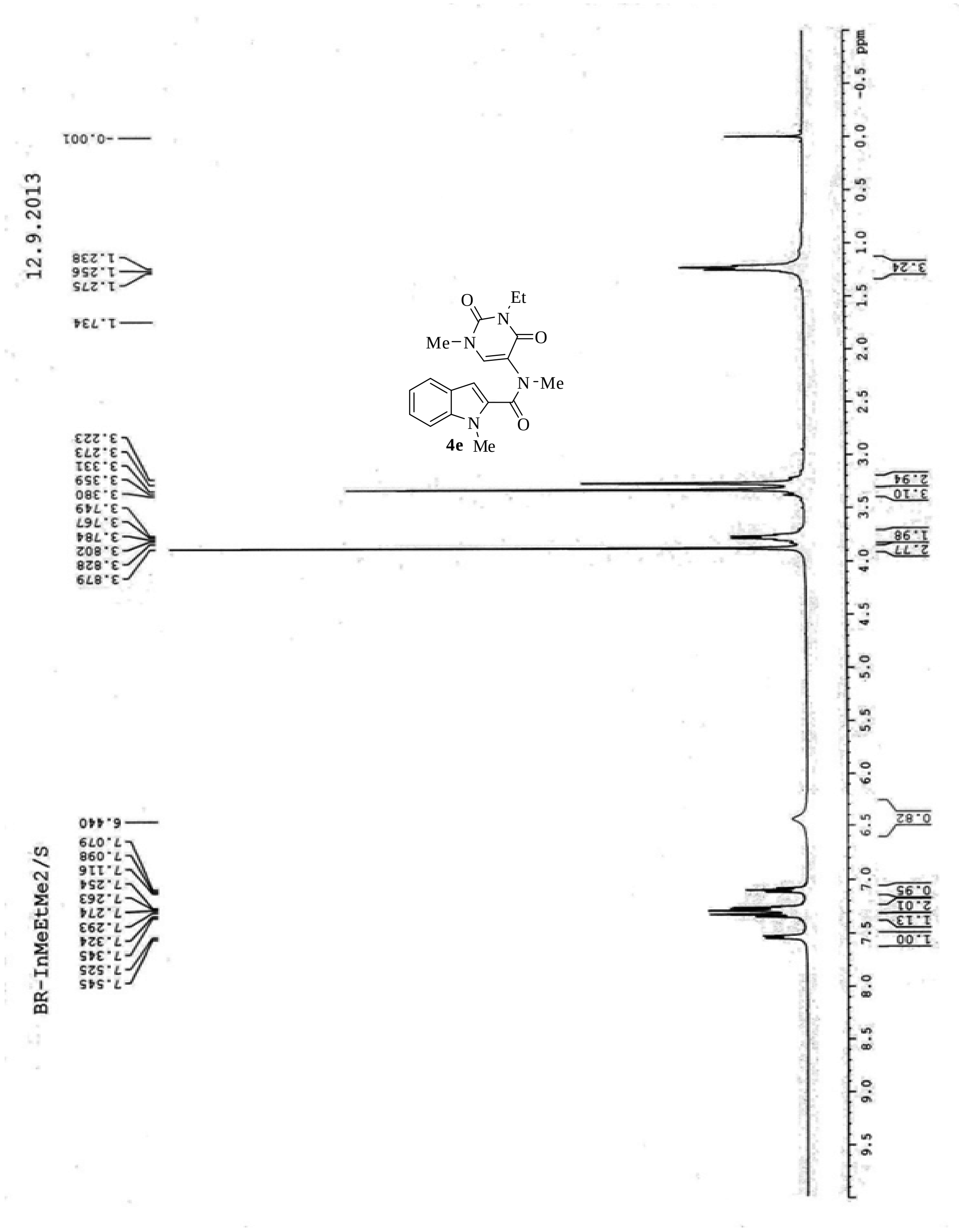

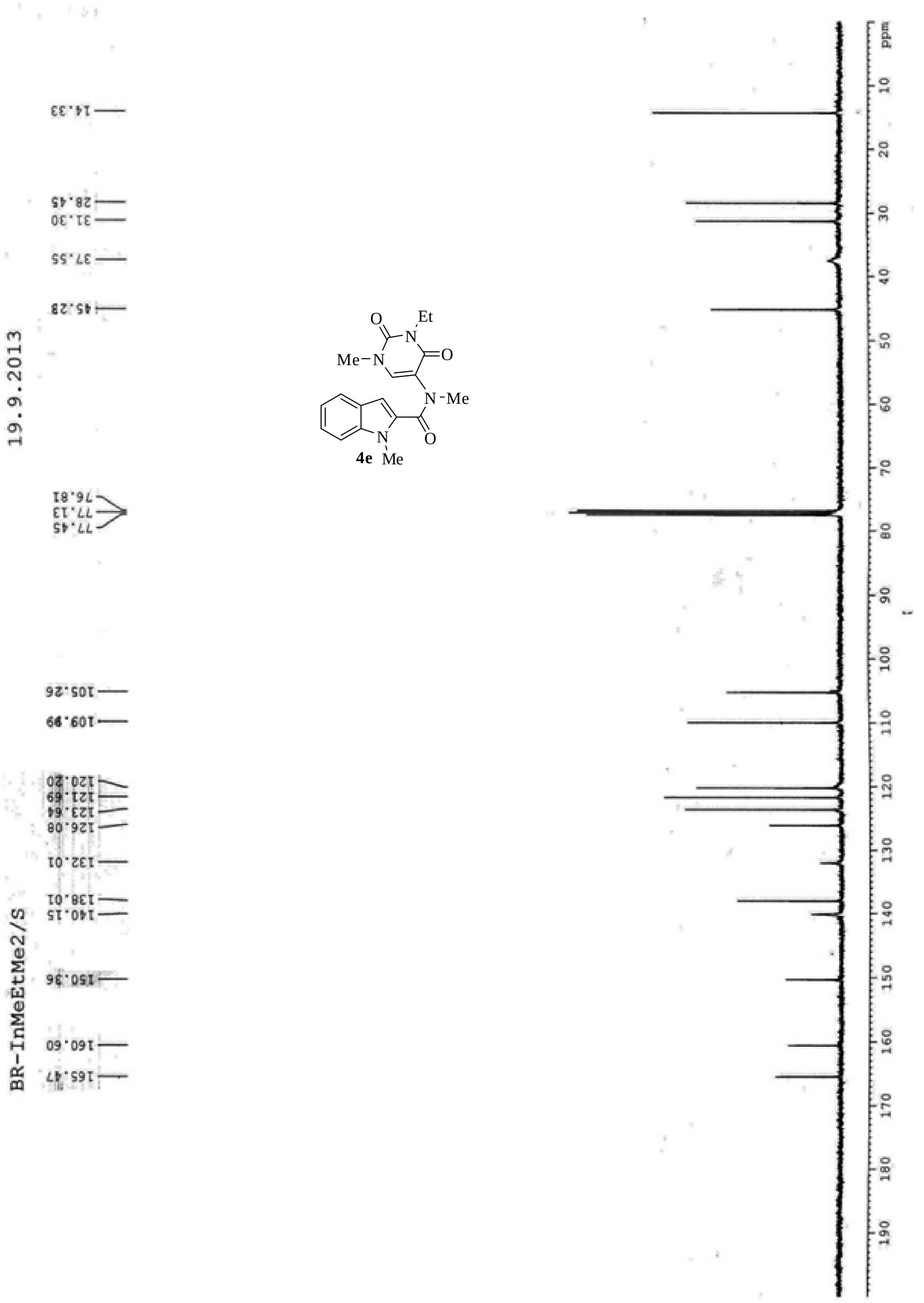

BR-InMeEtMe2/S
19.9.2013
14.33
28.45
31.30
37.55
45.28
76.81
77.13
77.45
105.26
109.99
120.20
121.69
123.64
126.08
132.01
138.01
140.15
150.36
160.60
165.47
4e
ppm

_N_-(1,3-Dimethyl-2,4-dioxo-1,2,3,4-tetrahydropyrimidin-5-yl)-1-ethyl-_N_-methyl-1_H_-indole-2-carboxamide (4f):

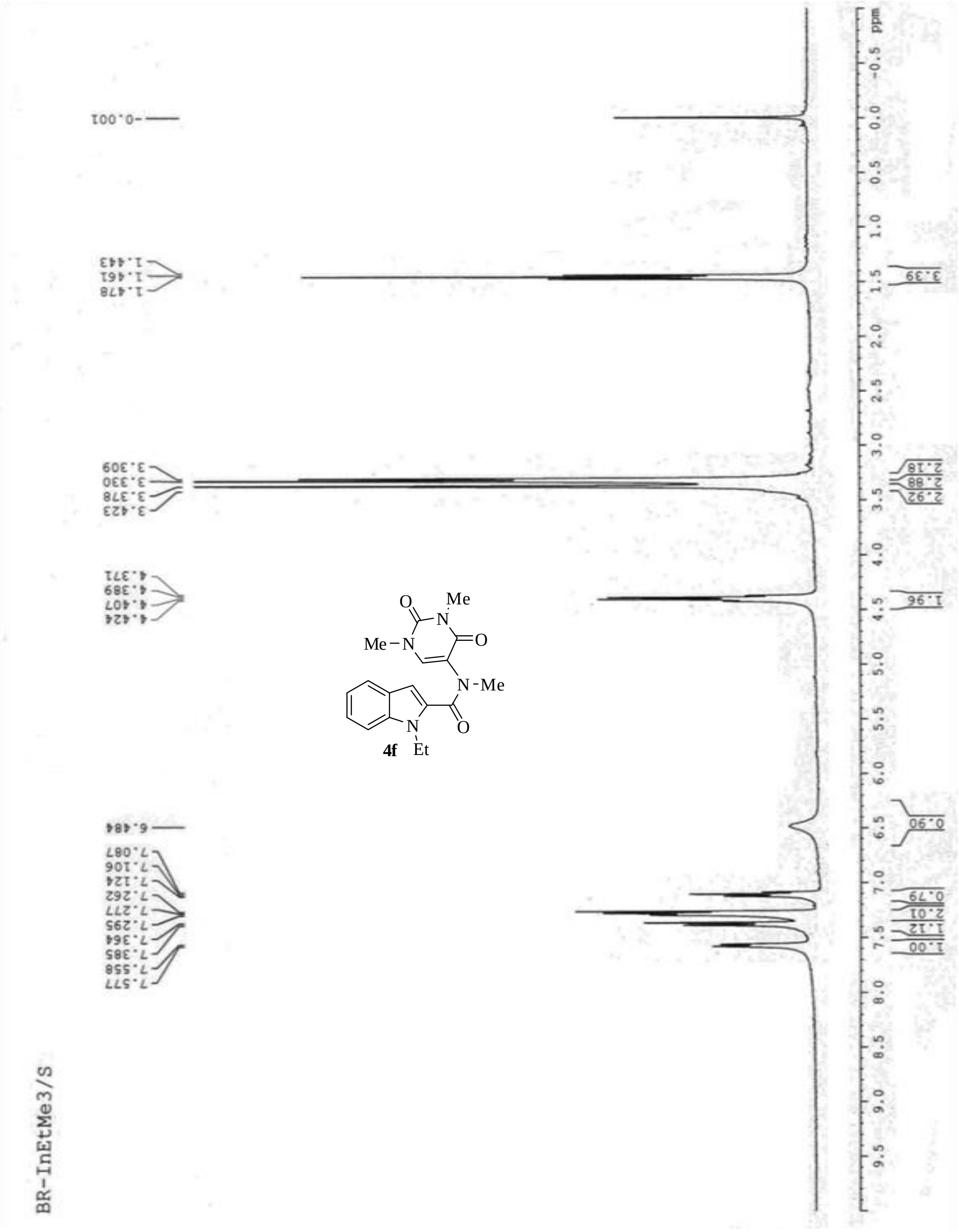

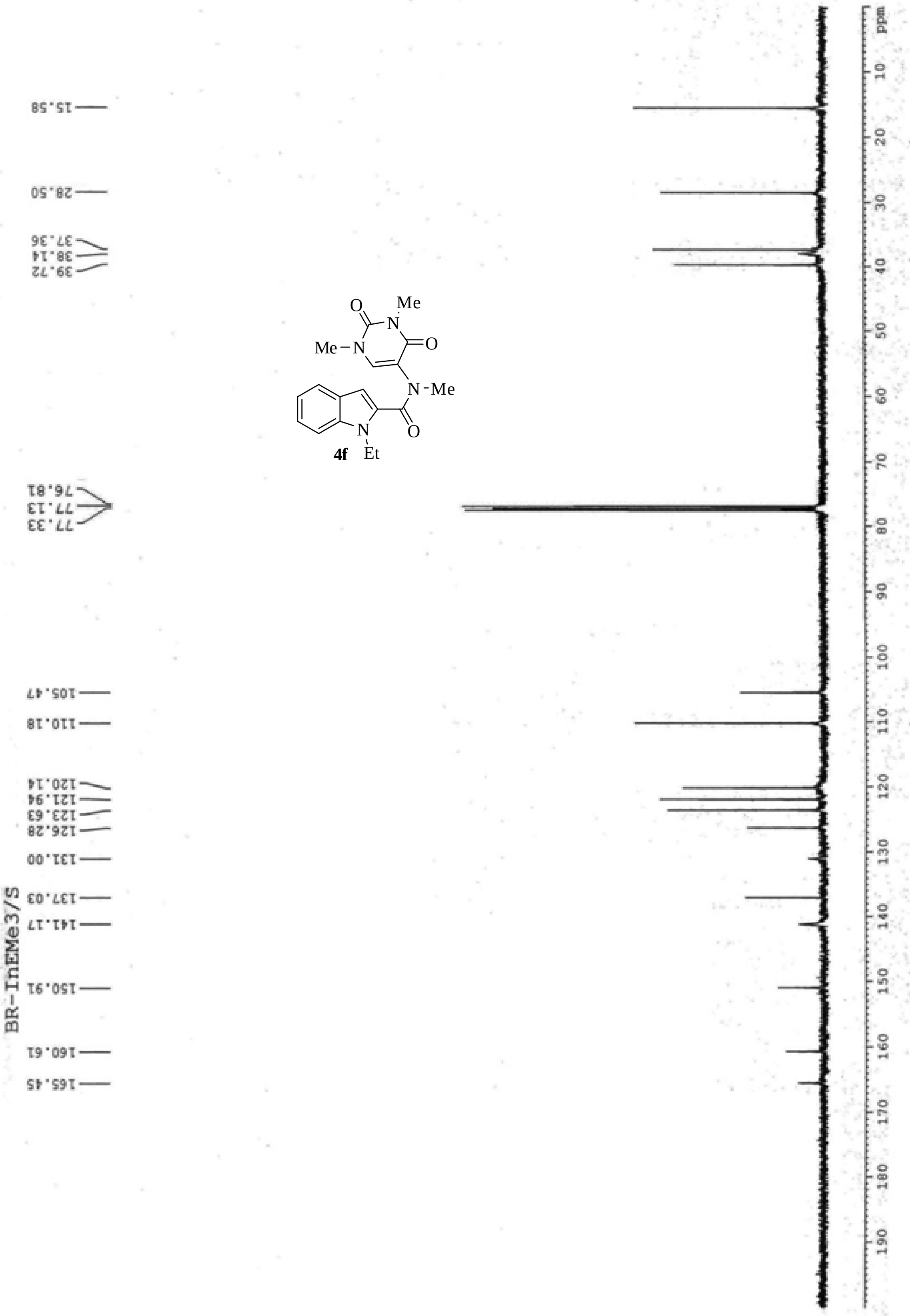
BR-InEMe3/S
15.58
28.50
37.36
38.14
39.72
76.81
77.13
77.33
105.47
110.18
120.14
121.94
123.63
126.28
131.00
137.03
141.17
150.91
160.61
165.45
ppm
Me
O
N
Me
N
O
N
Me
N
O
4f
Et

_N_-(1,3-Dimethyl-2,4-dioxo-1,2,3,4-tetrahydropyrimidin-5-yl)-_N_,1-diethyl-1_H_-indole-2-carboxamide (4g):

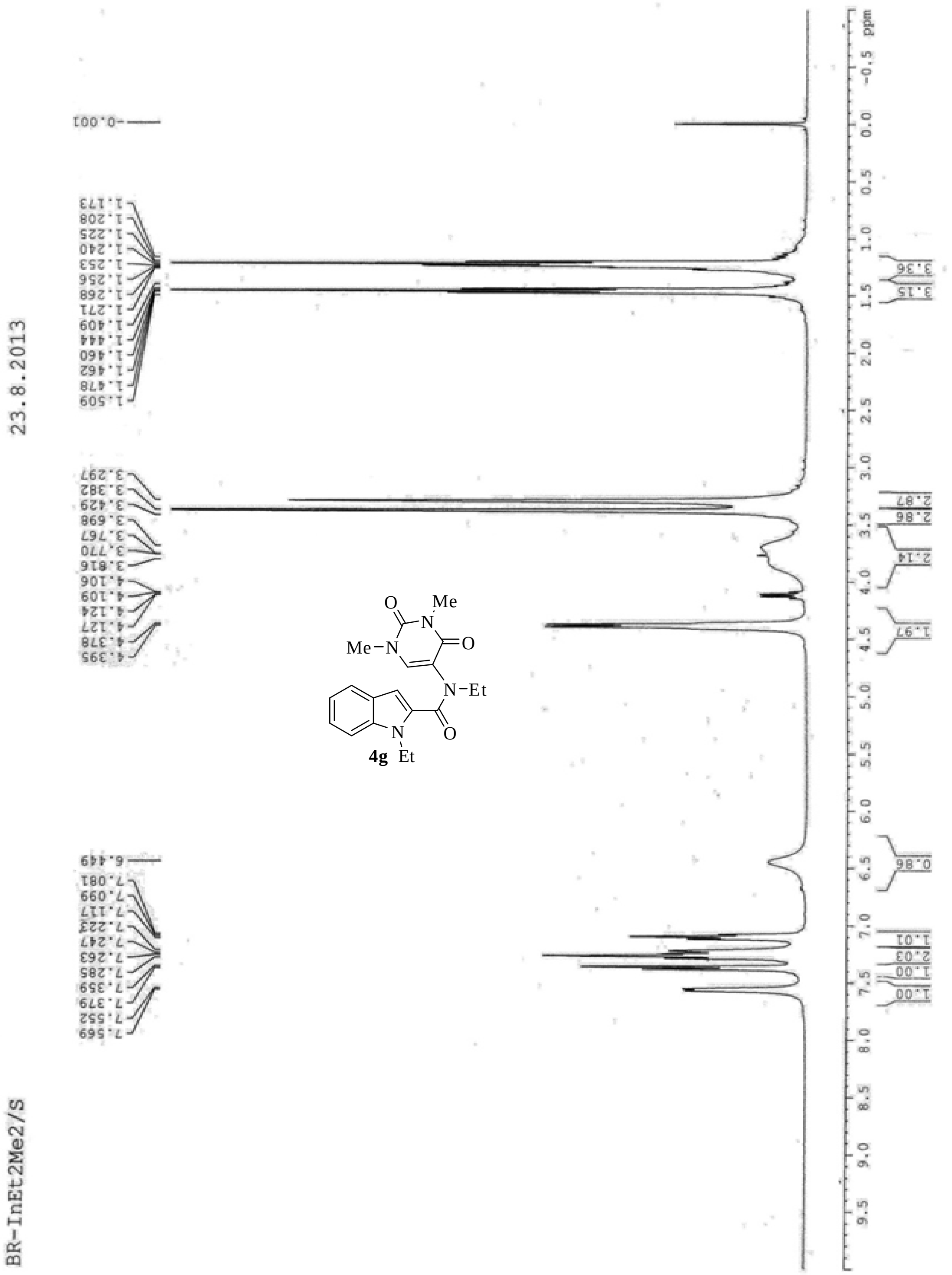

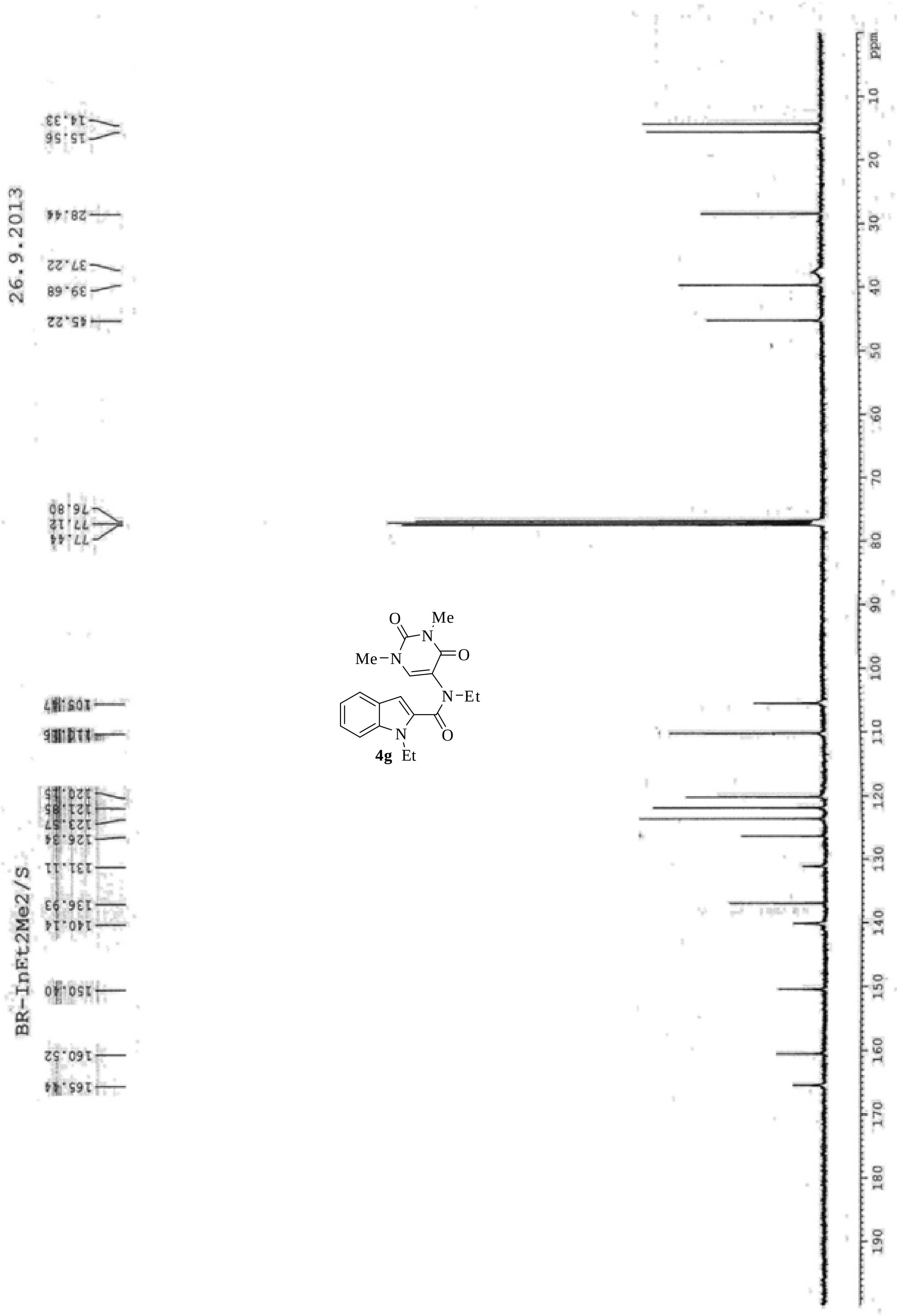

BR-InEt2Me2/S
26.9.2013
14.33
15.56
28.44
37.22
39.68
45.22
76.80
77.12
77.44
121.85
123.57
126.34
131.11
136.93
140.14
150.40
160.52
ppm
O
Me
N
Me−N
O
N−Et
N
O
Et

4g

***N*-(1,3-Diethyl-2,4-dioxo-1,2,3,4-tetrahydropyrimidin-5-yl)-1-ethyl-*N*-methyl-1*H*-indole-2-carboxamide (4h):**

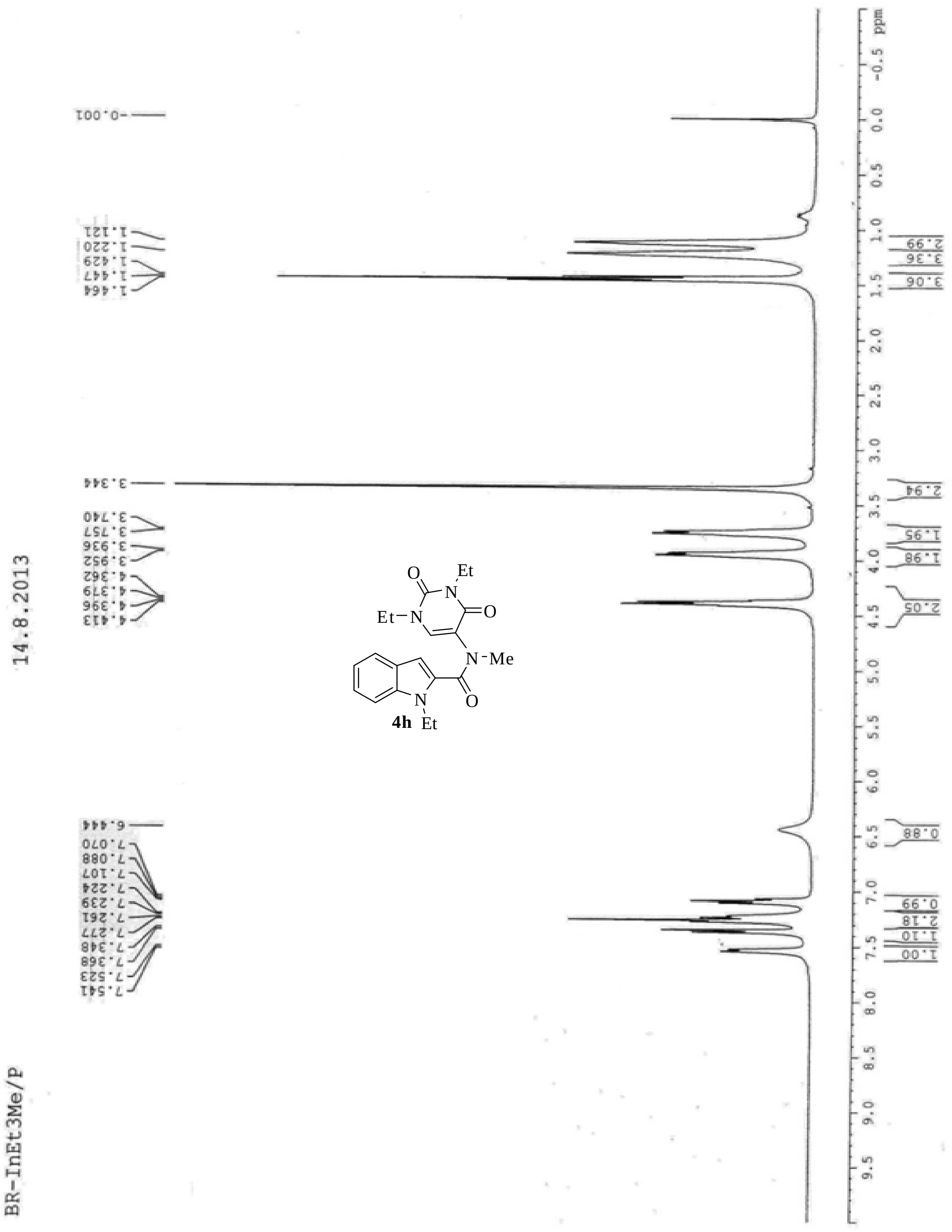

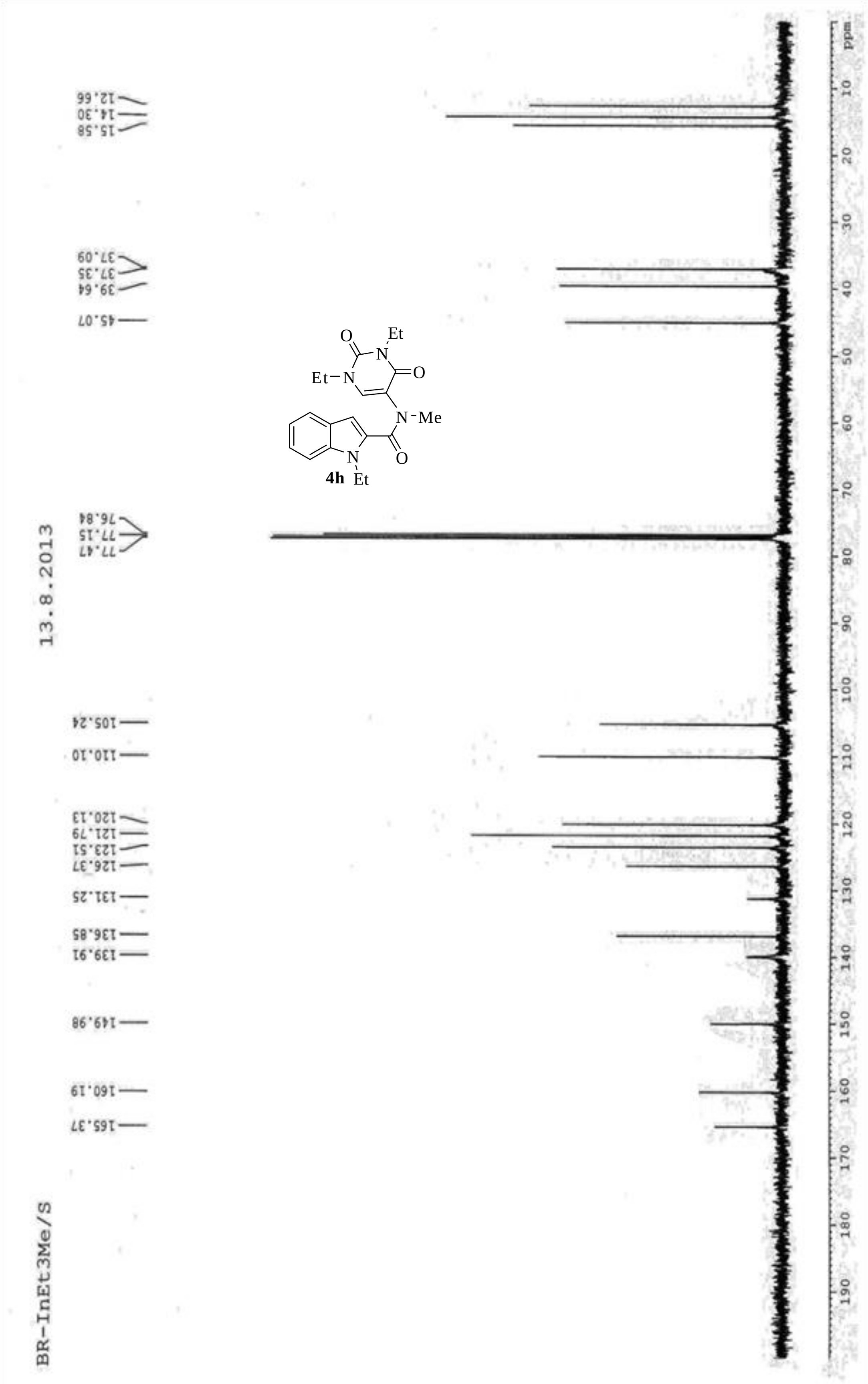

BR-InEt3Me/S
13.8.2013
12.66
14.30
15.58
37.09
37.35
39.64
45.07
76.84
77.15
77.47
105.24
110.10
120.13
121.79
123.51
126.37
131.25
136.85
139.91
149.98
160.19
165.37
Et
O
N
Et–N
O
N–Me
O
N
4h
Et
ppm

1-Benzyl-*N*-(1,3-diethyl-2,4-dioxo-1,2,3,4-tetrahydropyrimidin-5-yl)-*N*-ethyl-1*H*-indole-2-carboxamide (4i):

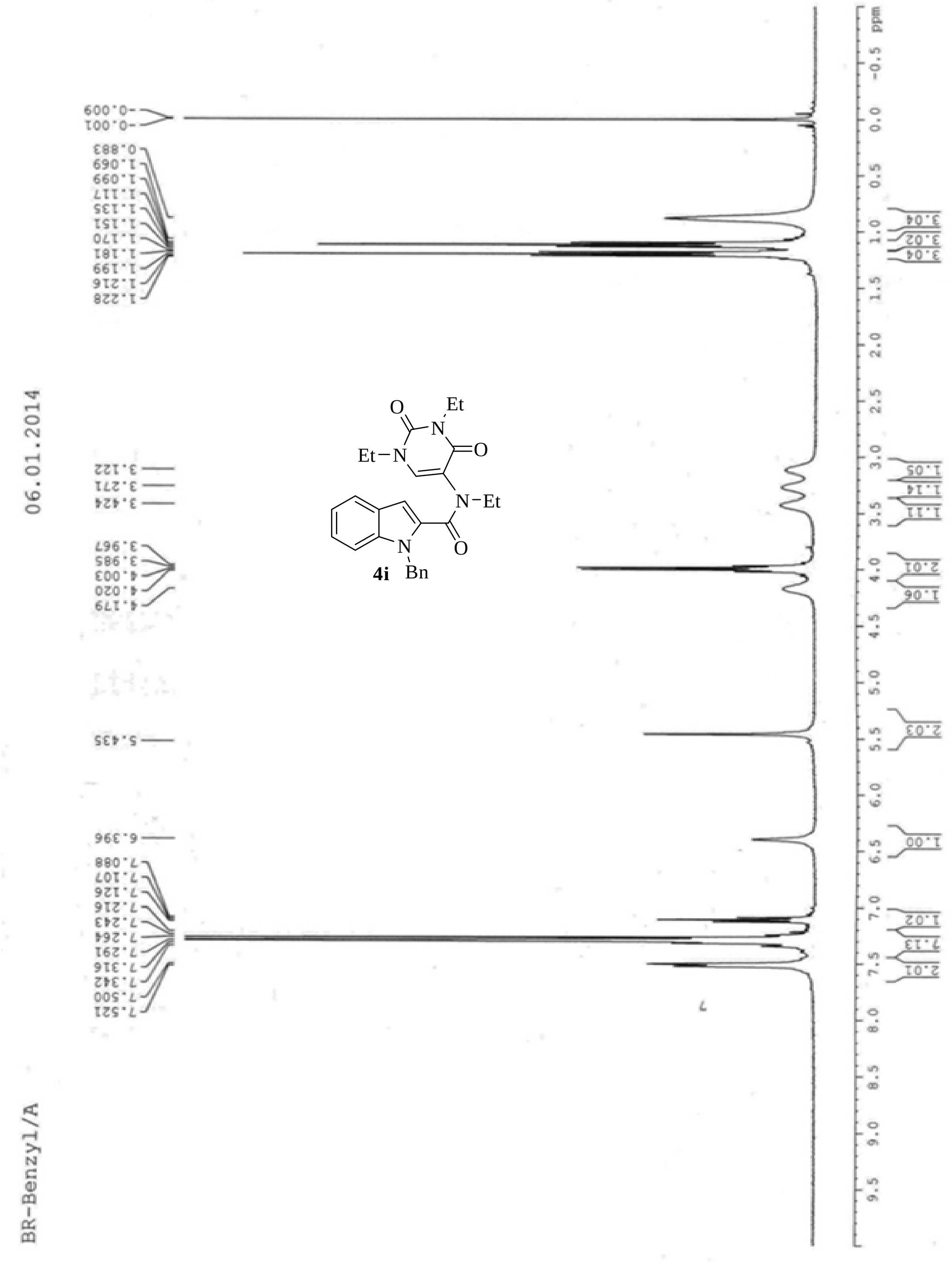

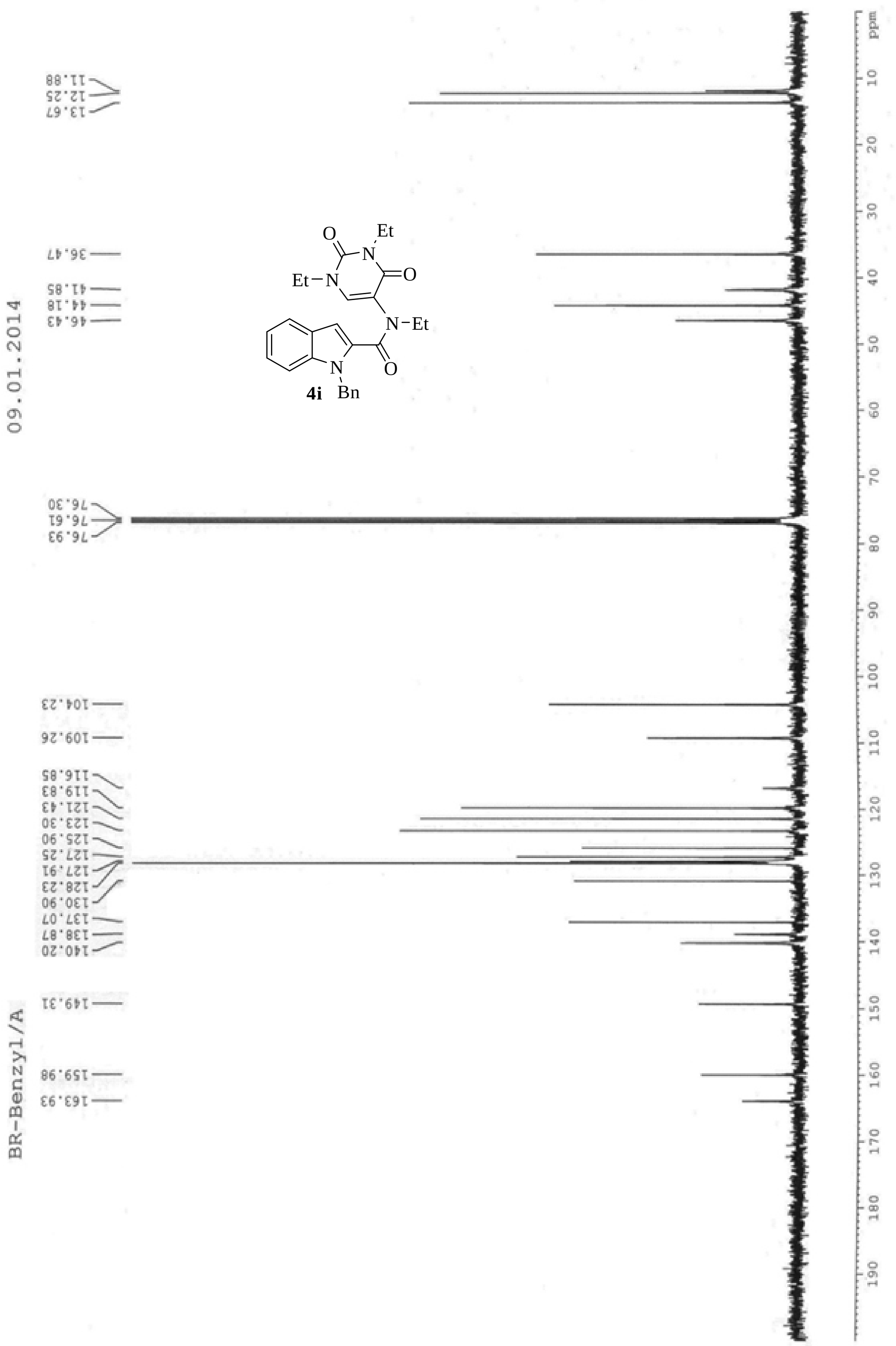

BR-Benzyl1/A
09.01.2014
163.93
159.98
149.31
140.20
138.87
137.07
130.90
128.23
127.91
127.25
125.90
123.30
121.43
119.83
116.85
109.26
104.23
76.93
76.61
76.30
46.43
44.18
41.85
36.47
13.67
12.25
11.88
ppm
4i
Bn
Et

1-Benzyl-*N*-(1,3-diethyl-2,4-dioxo-1,2,3,4-tetrahydropyrimidin-5-yl)-*N*-methyl-1*H*-indole-2-carboxamide (4j):

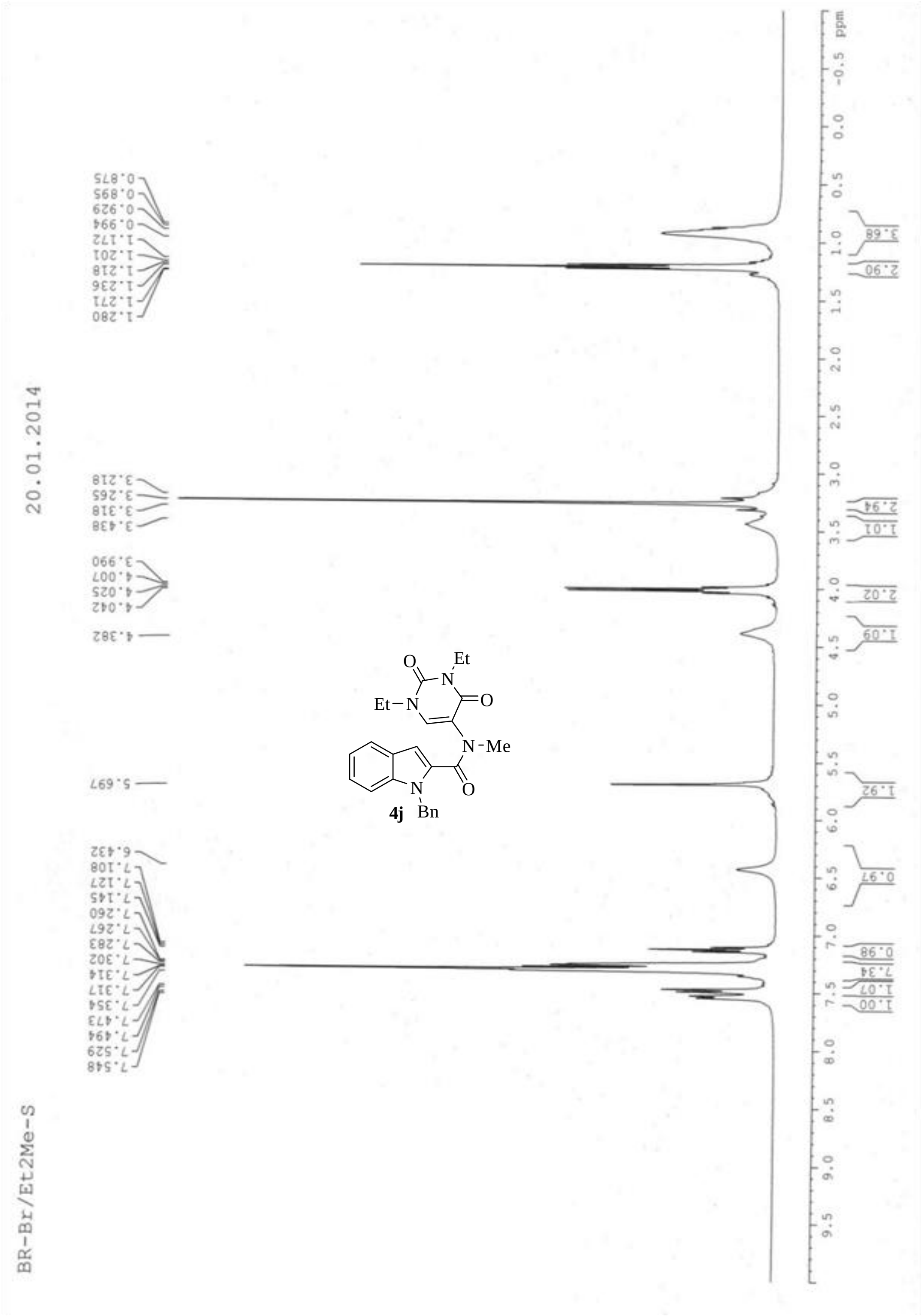

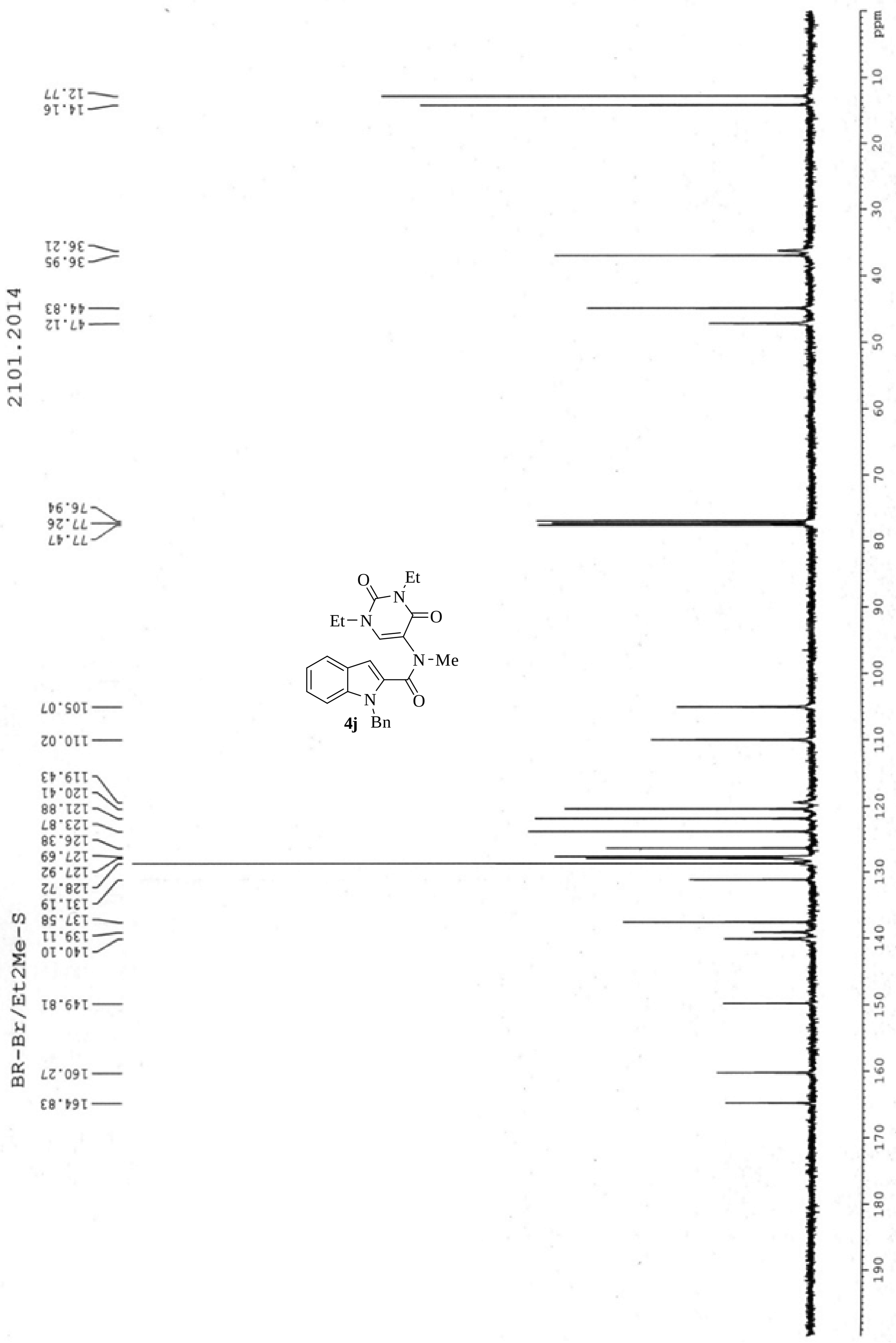
BR-Br/Et2Me-S
2101.2014
12.77
14.16
36.21
36.95
44.83
47.12
76.94
77.26
77.47
105.07
110.02
119.43
120.41
121.88
123.87
126.38
127.69
127.92
128.72
131.19
137.58
139.11
140.10
149.81
160.27
164.83
ppm
O
Et
N
Et
N
O
N
Me
N
O
4j
Bn

1-Butyl-*N*-(1,3-diethyl-2,4-dioxo-1,2,3,4-tetrahydropyrimidin-5-yl)-*N*-methyl-1*H*-indole-2-carboxamide (4k):

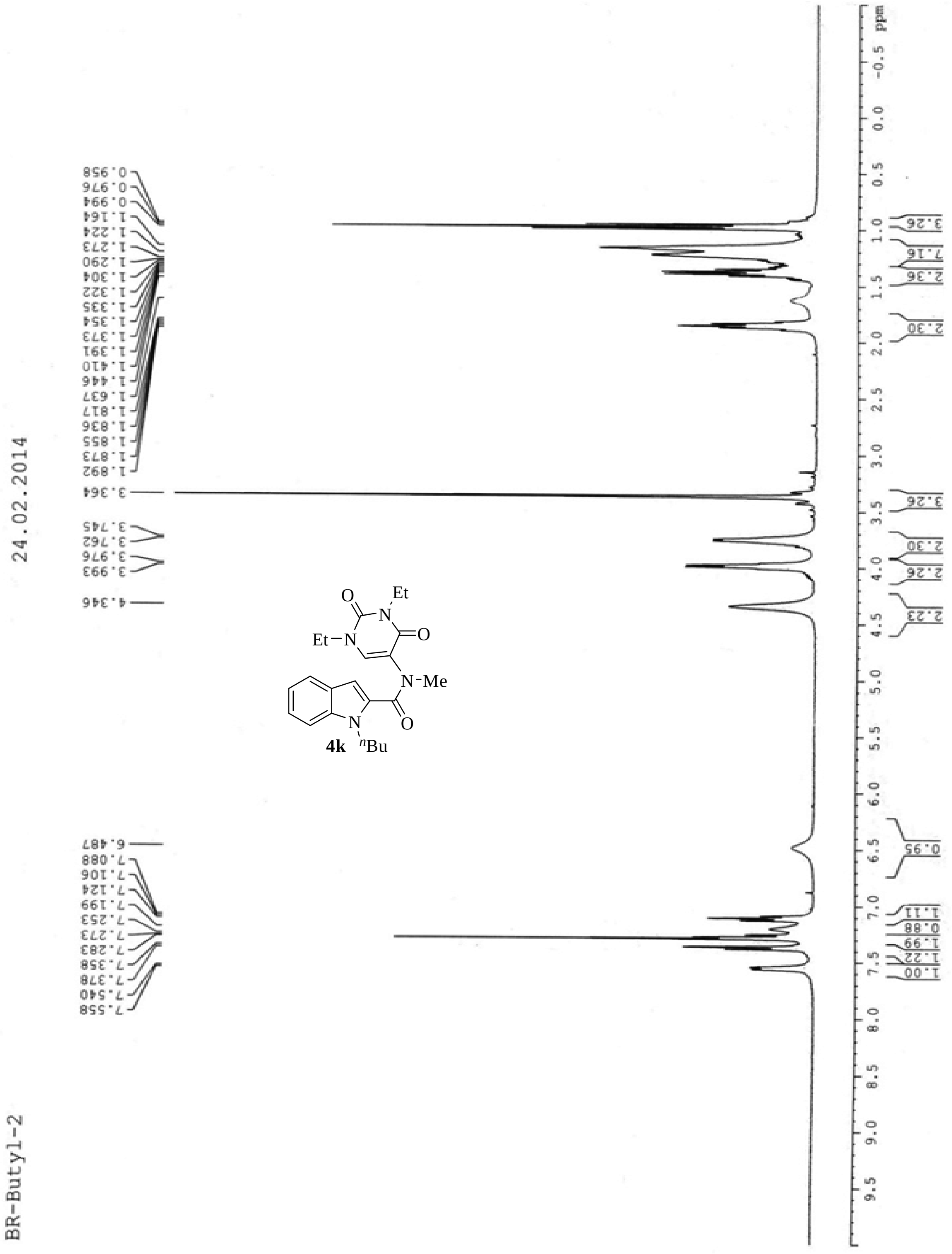

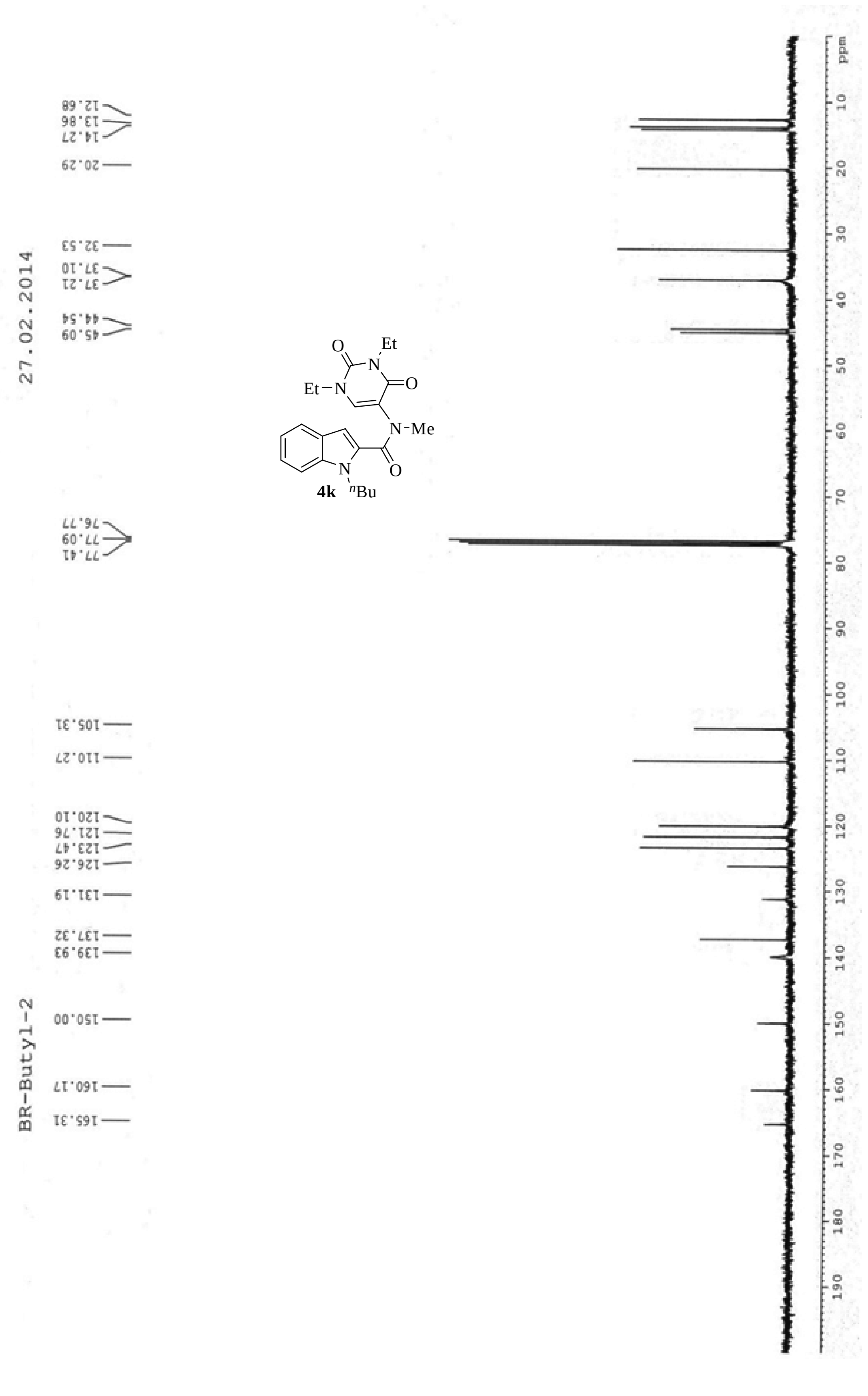

BR-Butyl-2
27.02.2014
12.68
13.86
14.27
20.29
32.53
37.10
37.21
44.54
45.09
76.77
77.09
77.41
105.31
110.27
120.10
121.76
123.47
126.26
131.19
137.32
139.93
150.00
160.17
165.31
4k
ppm

1-Butyl-*N*-(1,3-diethyl-2,4-dioxo-1,2,3,4-tetrahydropyrimidin-5-yl)-*N*-ethyl-1*H*-indole-2-carboxamide (4l):

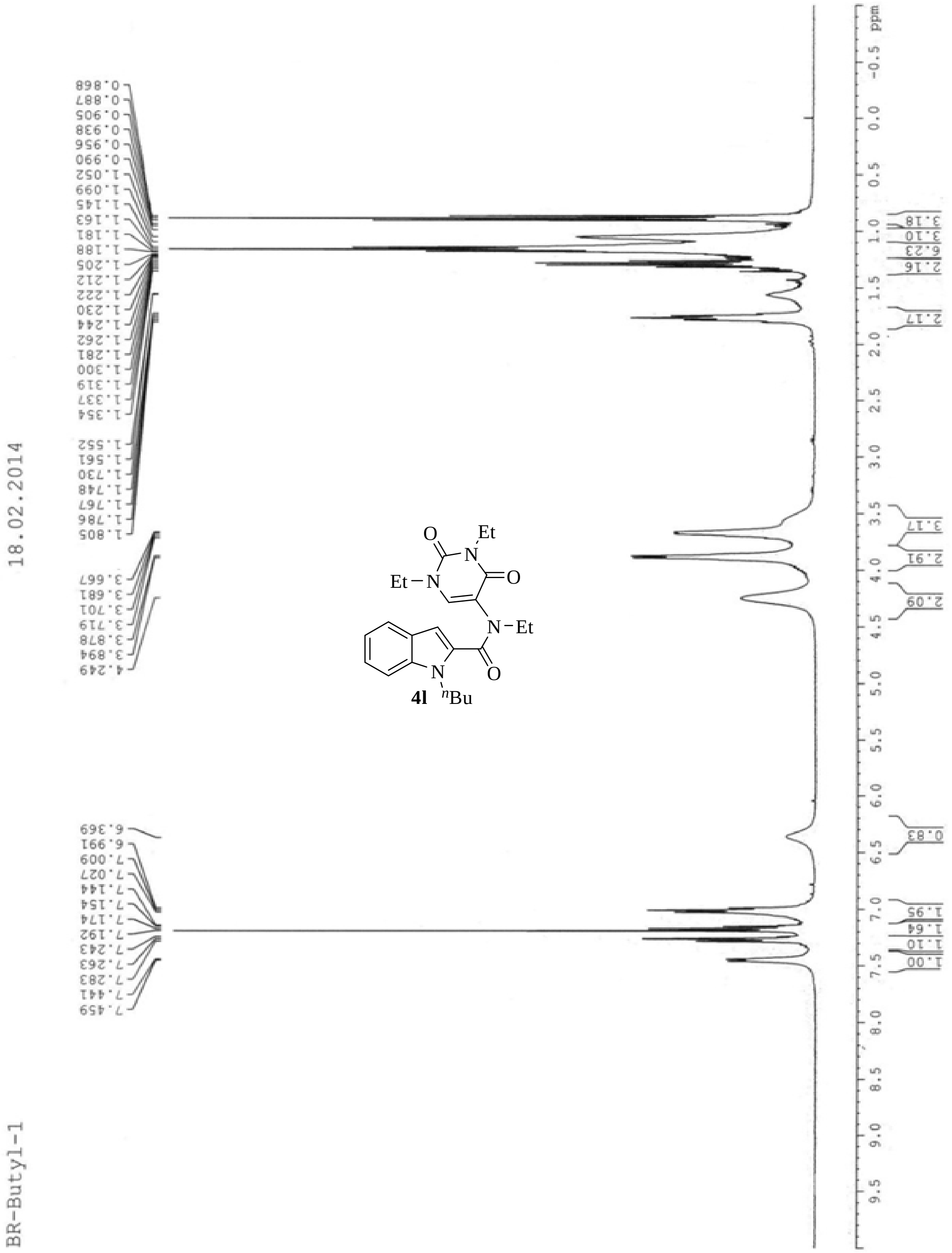

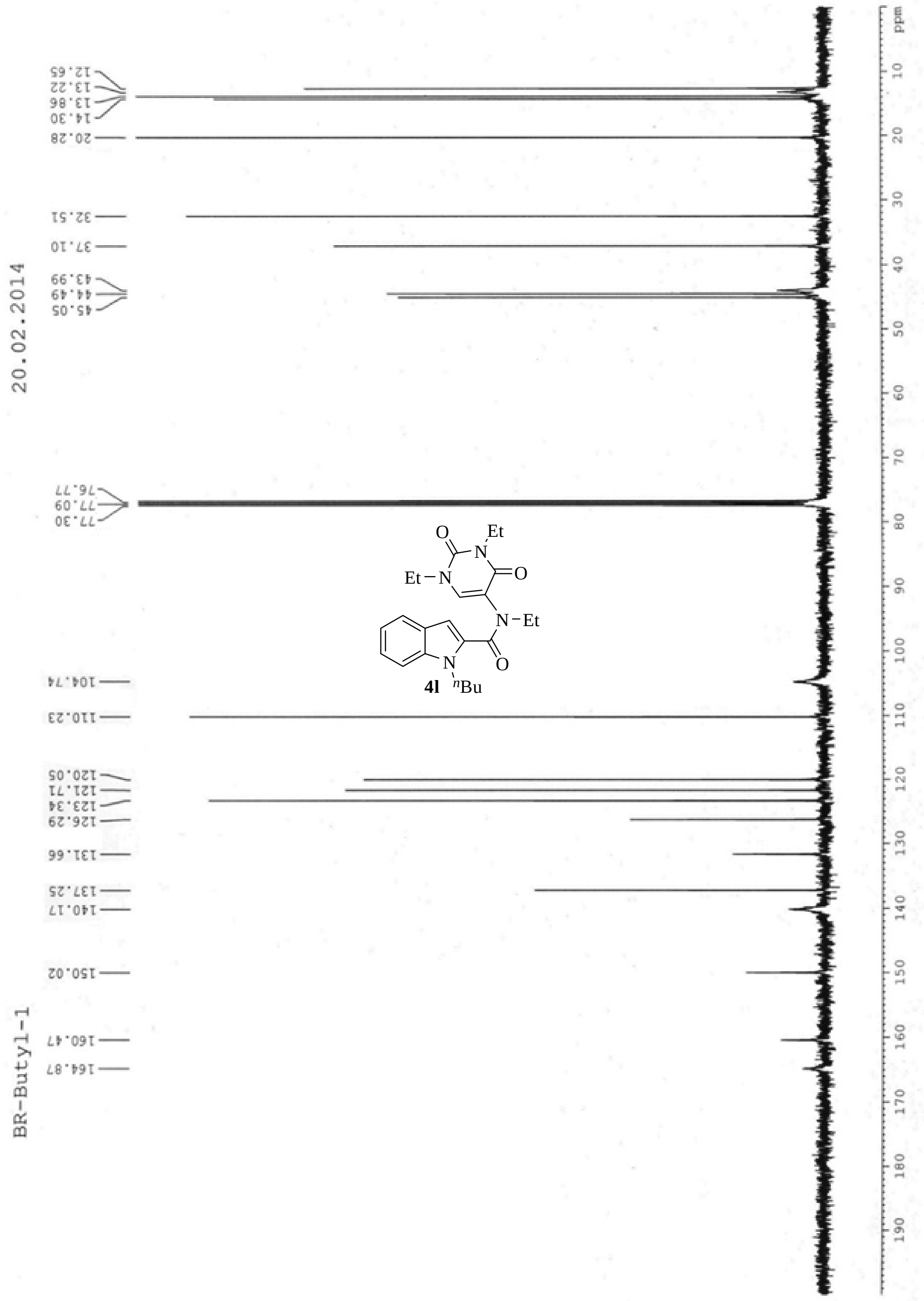
20.02.2014
BR-Butyl-1
12.65
13.22
13.86
14.30
20.28
32.51
37.10
43.99
44.49
45.05
76.77
77.09
77.30
104.74
110.23
120.05
121.71
123.34
126.29
131.66
137.25
140.17
150.02
160.47
164.87
ppm
4l

***N*-(1,3-Diethyl-2,4-dioxo-1,2,3,4-tetrahydropyrimidin-5-yl)-*N*-methyl-1*H*-indole-2-carboxamide (4m):**

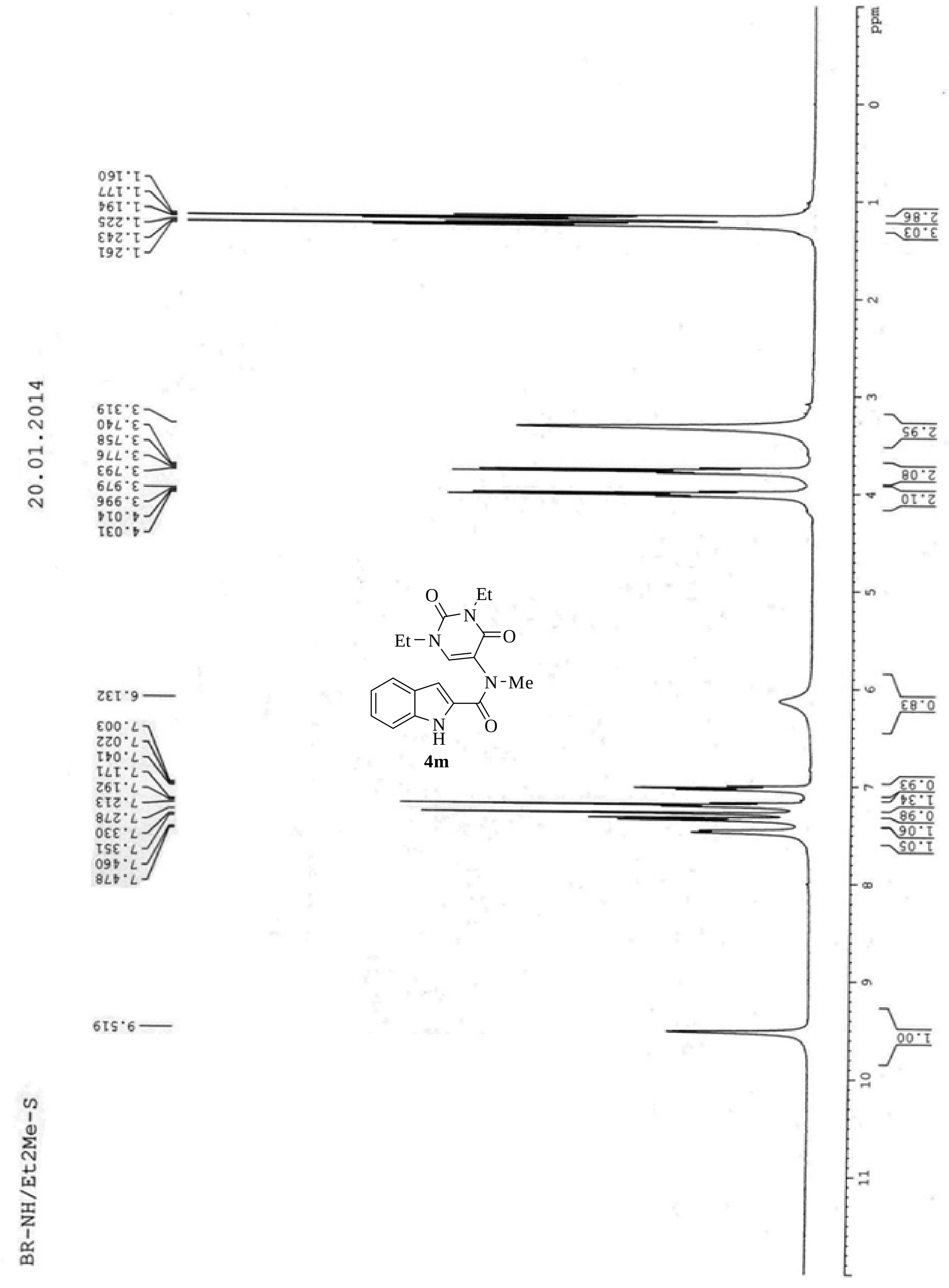

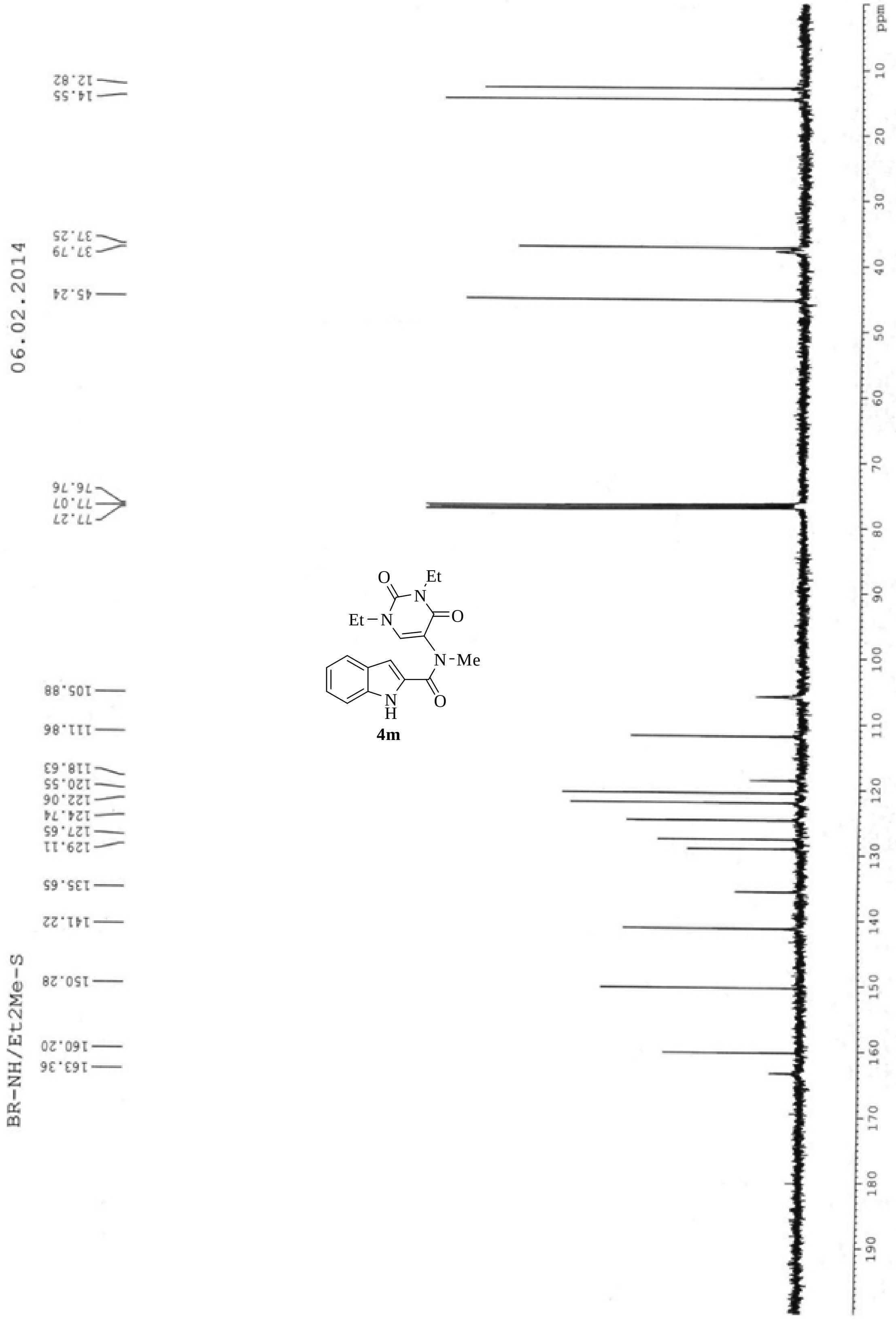

BR-NH/Et2Me-S
06.02.2014
12.82
14.55
37.25
37.79
45.24
76.76
77.07
77.27
105.88
111.86
118.63
120.55
122.06
124.74
127.65
129.11
135.65
141.22
150.28
160.20
163.36
ppm
O
Et
N
Et
N
O
N
Me
H
O

4m
